# Predicting the Willingness and Purchase of Travel Insurance During the COVID-19 Pandemic

**DOI:** 10.3389/fpubh.2022.907005

**Published:** 2022-07-04

**Authors:** Abdullah Al Mamun, Muhammad Khalilur Rahman, Qing Yang, Taslima Jannat, Anas A. Salameh, Syed Ali Fazal

**Affiliations:** ^1^UKM - Graduate School of Business, Universiti Kebangsaan Malaysia, Bangi, Malaysia; ^2^Faculty of Entrepreneurship and Business, Universiti Malaysia Kelantan, Kota Bharu, Malaysia; ^3^College of Business Administration, Prince Sattam Bin Abdulaziz University, Al-Kharj, Saudi Arabia; ^4^Faculty of Business Administration, University of Science and Technology Chittagong, Chittagong, Bangladesh

**Keywords:** travel insurance, theory of planned behavior, working adults, Malaysia, COVID-19

## Abstract

This study explored the willingness and purchase of travel insurance during the COVID-19 pandemic amongst working adults to ensure their safety and welfare through the lens of the theory of planned behavior. Primary data were gathered from 1,118 working adults across Malaysia and analyzed using the partial least squares structural equation modeling. The study outcomes revealed that attitude toward travel insurance was significantly influenced by insurance literacy, perceived health risk, and health consciousness. The willingness of working adults to purchase travel insurance was highly influenced by attitudes, subjective norms, and perceived behavioral controls but unaffected by perceived product risks. The purchase of travel insurance was positively influenced by the willingness to purchase travel insurance. In fact, travel insurance literacy and perceived health risk should be emphasized amongst working adults to encourage them to purchase travel insurance policies for traveling abroad.

## Introduction

Travel insurance endorses travel and healthcare costs incurred by tourists abroad. It organizes the aeromedical departure of tourists under circumstances determined by the travel insurance policy (1, 2). Travelers are always highly recommended to purchase travel insurance based on several essential requirements. Travelers need to read their insurance policies cautiously to perceive the covered aspects and to detect prohibitions, if any. It is also integral for those working abroad to purchase a travel insurance policy (2). Previous studies reported that 22–64% of global tourists fell ill during or after travel (3–5), but this information might be outdated and limited by poor generalisability. With the increasing number of international tourists, travelers may be at risk of catching infectious diseases (6). The risk of being infected with the deleterious virus is a petrifying thought, often, amongst those traveling overseas. Both perceived health consciousness (HC) and insurance literacy (IL) can mitigate such risk, yet only a handful of studies have explored the decision made by working adults to purchase travel insurance. As health risks (HR) may be sufficiently covered by travel insurance to protect travelers, travel agencies should regularly advise about travel insurance.

Risks always seem to be a travel companion (7) due to global healthcare outbreaks, international conflicts, and the recent spread of coronavirus. Consumers' willingness to purchase travel insurance (WTI) and purchase of travel insurance (PTI) policies are crucial in reducing the potential HR outcomes. Prior studies have investigated the uncertainty and risk reduction policies for traveling to different destinations (8, 9). Although a couple of studies on travel insurance have highlighted the healthcare implications (10–12), only a few studies have assessed travel insurance as IL, and particularly, HR and consciousness in determining consumers' willingness to and PTI. As risky circumstances denote undesirable attributes whilst traveling abroad (13), viable strategies are essential to minimize uncertainty, apart from ascertaining the safety and security of travelers (1). Insurers should also conduct marketing and communication campaigns to regain traveler' confidence during this COVID-19 pandemic (6, 14).

Although COVID-19 infected individuals over 50 years old have a higher hospitalization and mortality rate, the majority of those infected and hospitalized with COVID-19 are between the ages of 18–29 years, and a significant number of infected patients aged 1–49 years old have died of this virus until now (15). According to Mahmud et al. (16), the number of COVID-19 infection cases and deaths in the United States and other countries that use a 100% effective vaccination program will be significantly reduced by 2026 or else the virus will take longer to control. Malaysia and several other countries around the world used a combination of vaccines other than Pfizer to protect their citizens from the COVID-19 pandemic. According to a recent report, around 82% of the Malaysians are fully vaccinated, with 61% receiving Pfizer vaccines (17), indicating a lower likelihood of quickly controlling the virus because 39% of the people received other vaccines (16) that are not considered 100% effective. Therefore, travel insurance research is crucial during the COVID-19 pandemic, particularly in a tourism-friendly country like Malaysia.

Travel insurance refers to products and services that cover unexpected losses incurred whilst traveling abroad and local. Insurance agencies offer travel insurance to service companies, intermediary travel destinations, and individual travelers. From the lens of the theory of planned behavior (TPB) (18, 19), along with its additional themes and structural model analysis, this study looked into the attitude toward travel insurance (AT), willingness to, and PTI amongst Malaysian working adults, which dictated their decision when choosing a travel insurance plan. The TPB has strong face validity as it has been reckoned as a prominent theory in the area of the social science domain that operationalises one's behavior or personal construct toward their choice or willingness to purchase products and services. Personal constructs are significant as they are recommended to be the model that guides the behavior of consumers (7) toward travel insurance. In the context of travel insurance during the COVID-19 pandemic, this study (i) investigated IL, HR, and consciousness toward travel insurance; (ii) identifiedAT, subjective norm (SN), perceived product risk (PR), and behavioral control that reflected the consumers' WTI; and (iii) explored the consumers' intention that influenced the PTI. Researchers have argued in previous studies that policymakers should take a more disciplined action to combat the looming challenges associated with the COVID-19 infection (6, 20).

This study significantly contributes to both theory and practice. Based on the TPB (19), this study probed into an approach by which working adults were to expect the event of travel and how it encouraged their WTI. This study mainly focused on the attitude (IL, perceived risk, and consciousness) of working adults and the behavioral control that either encouraged or discouraged purchasing travel insurance.

### Study Context: Travel Insurance Industry in Malaysia

Malaysia has plenty of rivers, jungles, and wild rainforests, along with an abundance of picturesque beaches, national parks, sumptuous food, and idyllic islands. The country shares its border with Indonesia, Brunei, Thailand, and Singapore. The population of the country was over 32 million in 2019 and the climate is mostly tropical. Foreign nationals who visit Malaysia are required to have a valid national passport and adequate travel documents. The Malaysian Association of Tourist and Travel Agents (MATTA) has imposed travel insurance on its members, along with the provision of facilities and hotels for tourists (21). Travel agents offer travel insurance benefits to their clients for their travel safety and HR reduction (22). Malaysia has a Tourism Industry Compensation Fund that protects outbound travelers (23) through the PTI. The local outbound travel agency is required to have a minimum paid-up capital of RM 200,000 for locals/foreigners in the tour and travel agency business (24). For the protection of outbound travelers, the travel agency should purchase an insurance policy worth RM 100,000. Hasan and Abdullah (24) reported that the local inbound travel agency is required to pay a capital of RM50,000 and RM200,000 for rural and city tours, respectively.

## Literature Review

### Theoretical Foundation

The theory of planned behavior (TPB) has been widely applied to predict and explain human behavior (19, 25, 26). The TPB elaborates on factors linked with human characteristics that can lead to purchasing decisions amongst consumers (27, 28). From the lens of TPB, one's behavioral intentions are described as a consequence of attitudes, perceived behavioral control (PB), and SNssubjective norm. The TPB has been vastly applied in travel choice, healthcare behavior, commodity purchase, green purchase, and environmental behavior (25). Most of the studies reported that TPB has great explanatory power for consumers' purchase intentions. Thus, this study adopted the TPB to evaluate the behavior of working adults toward the PTI, apart from exploring the crucial influential factors. This study emphasized the success factors of purchasing travel insurance amongst working adults in Malaysia.

### Determinants of Attitude Toward Travel Insurance (AT)

#### Insurance Literacy (IL)

IL facilitates consumers in making the decision to purchase an insurance policy (29, 30). James et al. (31) asserted that health IL can influence healthcare application. IL is not well-established for travel insurance. As such, this study examined the impact of travel IL on the AT amongst working adults. Young adults tend to face financial barriers in light of healthcare services (31, 32); thus, they face difficulty when traveling abroad due to out-of-pocket costs for services. Given that most adults are interested in traveling abroad and they have health insurance, it is essential to address IL for their travel safety and security, which could influence their AT. Bartholomae et al. (33) reported that IL can affect both the knowledge and the ability to choose healthcare coverage. Working adults are likely to travel abroad for a range of purposes, including leisure and entertainment, thus making healthcare plans for travel insurance with crucial implications for their travel safety and welfare. However, one barrier to their decision is a limited understanding of travel insurance literature.

#### Perceived Health Risk (HR)

Typically, HR refers to adverse health consequences due to certain events, conditions, or diseases (34, 35). The HR is crucial in most of the health-related behavioral theories (36), including the health belief model (37) and the TPB (38). As for this study, the HR is theorized as a subjective evaluation of suffering healthcare events over a travel period abroad. Based on a meta-analysis, Brewer et al. (39) assessed the relationship between adult vaccinations and HR, which later identified a significant link between risk perception and intention of avoiding HR. Deng and Liu (40) claimed that improper use of health information may have HRs that could negatively influence consumers' attitudes or behaviors. Accordingly, this study assumed a significant relationship between HR and AT.

#### Health Consciousness (HC)

HC is the extent to which people tend to accept healthcare services (41, 42). Chen (43) reported that HC influences consumers' attitude. HC is a vital driver (44) that affects consumers' AT. Lee et al. (45) revealed the importance of HC to enhance the good health of young adults in the southwestern USA. The perception of young adults toward health may alter due to changes that take place in their personal life (i.e., their desire for a healthy lifestyle and preference for HC whilst traveling abroad). For this reason, providing information regarding travel insurance policy is crucial and travelers need to be aware of their health. Hoque et al. (42) and Chen and Li (46) discovered that the HC of consumers was positively related to attitude toward purchasing liquid milk and genetically modified foods.

### Determinants of Willingness to Purchase Travel Insurance (WTI)

#### Attitude Toward Travel Insurance

Attitude refers to one's psychological propensity that reflects customers' decision-making toward products and services. The TPB was employed in this study to explain the willingness of working adults to purchase travel insurance. Ajzen (19) used TPB to clarify one's intention in displaying a particular behavior. The TPB assumes that a strong attitude reflects a higher intention or willingness to purchase products and services. This study explained the attitude of working adults toward the WTI. A stronger attitude can better influence the tendency of consumers to visit travel destinations (47, 48). Salehzadeh and Pool (49) identified that consumer attitude was positively associated with perceived behavior. In light of TPB, attitude is a crucial predictor of consumers' willingness to execute purchase behavior. Liu et al. (50) reported on the interaction between attitude and travel intention. The more favorable one's AT, the higher is the WTI.

#### Perceived Product Risk (PR)

PR refers to one's perception of risk associated with the purchase of products and services. PR denotes risk with a stable impact on buyer's behavior (51–53). Perceived risk is related to loss of money and time; especially when expectations about the products and services are unmet (54). An insurance product is designed to cover costs and to reduce risks related to unexpected events during travel destinations. Previous studies explained that service quality minimizes the PR (55–57), which, in turn, may reflect consumers' WTI. Travel insurance can help consumers get a better sense of products and services whilst minimizing the risk when purchasing travel insurance. PR has an adverse impact on product perceptions (51), the attractiveness of online stores (52), consumers' trust (58), and product assessments (59). Nepomuceno et al. (53) discovered that PR has a strong negative relationship with customers' patronage intention or willingness to purchase a product. Hence, an empirical analysis was conducted in this study to shed light on the potential of travel insurance in reducing the PR of working adults when they are willing to purchase travel insurance.

#### Subjective Norm (SN)

An SN is a belief that one accepts and supports certain behavior. An SN is defined as a group of people or an individual's perception of social pressure to perform the behavior of interest (19, 60). Ham et al. (61) identified that the SN displayed a significant influence on consumers' intention or willingness to purchase green food. Kumar (62) assessed the strength of purchase intention and discovered that the SN was not significantly linked with purchase intention. Tarkiainen and Sundqvist (63) and Dalila et al. (64) revealed that the SN had directly affected the willingness of consumers to purchase products and services. Similarly, Kashif et al. (65) found that the SN strongly influenced the intention of customer service managers. From the lens of the TPB (38), this study measured the impact of the SN on the willingness of working adults to purchase travel insurance.

#### Perceived Behavioral Control (PB)

PB denotes the perceptions of an individual's ability and sense of control over a condition. According to Ajzen (66), PB is a belief about the combination of controls that one has on the outcomes in his life and about the ability to execute the task. Ajzen (18) explained that the intention of consumers is greatly affected by PB. In line with this, this study assumed that a stronger PB in working adults leads to a higher WTI. Based on TPB, consumers' PB denotes one's ability to conduct a specific behavior (18, 64, 67, 68), such as WTI. PB is an important determinant of consumers' willingness to purchase products and services (67, 69). Brahmana et al. (70) found that PB had a significant influence on the willingness of consumers to purchase health insurance.

#### WTI and PTI

Willingness to purchase refers to an individual's choice to purchase products and services (71). In this present study, the WTI is the monetary value that working adults are willing to compensate for some products or services. Despite the desire to own certain products, not all working adults are usually willing to purchase those desired products. Willingness is a realization that reflects one's willingness to purchase products or services (72, 73). Strong consumer willingness leads to higher purchase decisions of goods and services. WTI is, presumably, a better indicator than the intention to purchase goods or services, mainly because not all intention is transformed into the actual purchase decision. Song and Sun (74) revealed a significant relationship between traveler' willingness to purchase and the actual purchase of airline tickets. Hinnen et al. (75), who assessed consumers' willingness to pay for products in the air travel industry, reported that willingness to pay had a significant effect on the purchase of products. Hence, the following hypothesis is proposed:

### Moderating Effect of Income (INC)

A moderating construct displays the model complexity in providing a better perspective regarding the criterion variable of the study and is inconsistent in the literature for the relationship between determinant and criterion variable (72). Past studies have identified inconsistencies in constructs within the TPB related to consumers' behavior of willingness. Willingness, from the lens of TPB, refers to the cognitive predictor that involves consumers' behavior (76). This present study applied income as the moderator, because income is a fraction of the cognitive components (77). Income is gained by an individual or a group of people involved in personal business within the private or public segments. Sana et al. (78) claimed that those with higher income possess sufficient resources to purchase insurance schemes. Higher income can generate positive results for the relationship between willingness and purchase of products and services (74). Working adults earning higher income may have a stronger WTI products and services. In this case, one's higher income displays a stronger relationship between WTI and PTI.

### Mediating Effect of AT and WTI

Measuring the direct and indirect effects amongst the latent variables is to assess the causal relationship embedded in the theoretical model (42). The indirect effect implies the effect of exogenous constructs on endogenous indicators through the mediating construct (42, 79). In the travel insurance segment, consumers' attitudes can mediate the relationship between cues and willingness or purchase behavior. Attitude refers to a learned tendency to take action and may serve as an antecedent of consumers' willingness, intention, and behavior (80, 81). Ahmad et al. (82) revealed that attitude has a significant impact on purchase behavior amongst consumers. Harun et al. (80) reported that consumers' attitudes toward the purchase of products has either positively or negatively influenced their purchase behavior. This present study examined the effect of IL, HR, perceived HC, attitude, SN, PR, and PB on the willingness and purchase behavior. Willingness denotes a responsive situation to start the tendency to purchase goods and services (83). It reflects a psychological condition for people to develop their behavior. As consumers worldwide have diverse willingness in their purchase behavior (84) it is crucial to assess the effect of their willingness on purchase behavior.

**Figure 1:**
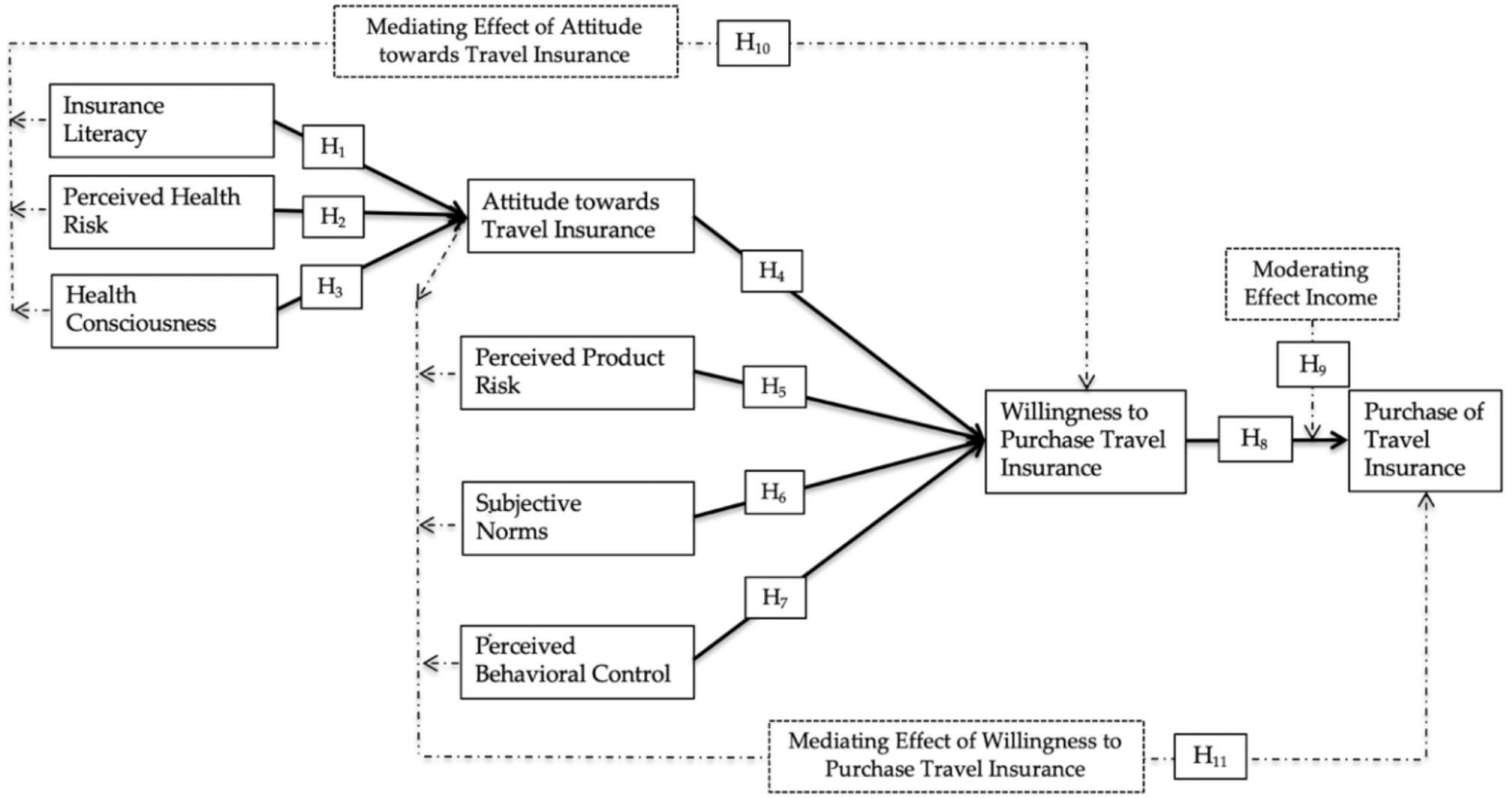
Research framework.

All associations hypothesized and examined are presented in Figure 1.

## Research Methodology

### Survey Design and Measurement

To determine the willingness and PTI during the COVID-19 pandemic, this study focused on the working adults in Malaysia. Due to the COVID-19 lockdown in Malaysia, this study adopted an online survey method using Google Forms. The online survey link and a cover letter explaining the purpose of the study are shared on social media platforms, including Facebook, Instagram, LinkedIn, WhatsApp groups, and through emails. This study also requested everyone who viewed the survey link to share it on their social media pages/groups as appropriate. The complete data were collected from 1,118 working adults across Malaysia between June and July 2020.

The questionnaire was composed of two sections. The first section gathered demographic information from the respondents, including gender, age, ethnicity, living area, marital status, education, income, and PTI. Meanwhile, the second section assessed the psychological-related factors that lead to respondents' WTI and PTI. Based on the TPB, latent variable items were used to evaluate the working adults' WTI. Each psychological-related factor was assessed by five items, except for IL and PR constructs that were examined based on a 5-point Likert scale (1 = strongly disagree and 5 = strongly agree). Amongst the constructs, IL consisted of four items adapted from Bartholomae et al. (33), whilst HR comprised five items modified from Brewer et al. (39) and Deng and Liu (40), and HC had five items retrieved from Chen and Li (46) and Hoque et al. (42). Attitude toward the PTI was composed of five items modified from Liu et al. (50), whereas PR comprised four items modified from Goedertier et al. (59) and Nepomuceno et al. (53). The SN had five items adapted from Dalila et al. (64) and Kashif et al. (65). PB consisted of five items modified from Xu et al. (67) and Elmorshidy (69). WTI and PTI had six items retrieved from Aziz et al. (85), Brahmana et al. (70), and Weedige et al. (86). Appendix 1 (see Supplementary Material - Data Sheet 2) lists all the items used to measure the study variables.

### Common Method Variance (CMV)

The single factor accounted for 31.52%, which is below the recommended threshold of 50% in Harman's one-factor test, thus approving the inconsequential influence of CMV on this study (87). Additionally, to establish the strength of the CMV evaluation, the correlations amongst the study's latent constructs were estimated, wherein a correlation that scores below 0.9 signifies the absence of CMV (87). Furthermore, this study evaluated the CMV by following Kock's (88) recommendation to test the full collinearity of all the constructs. All the study constructs retreated a common variable, and the variance inflation factor (VIF) value below the 3.3 value (see Table 1) designates the non-appearance of bias from the single-source data.

**Table 1:** Full collinearity test.

**IL**	**HR**	**HC**	**AT**	**PR**	**SN**	**PB**	**WTI**	**INC**	**PTI**
1.474	1.484	1.441	2.620	1.140	2.440	1.643	2.659	1.080	1.426

*IL, insurance literacy; HR, perceived health risk; HC, health consciousness; AT, attitude toward travel insurance; PR, perceived product risk; SN, subjective norms; PB, perceived behavioral control; WTI, willingness to purchase of travel insurance; INC, average monthly income; PTI, purchase of travel insurance*.

### Data Analysis Method

The multivariate normality test verified that the dataset was not normal, as Mardia's multivariate coefficient *p*-values were below 0.05 (89). Because of the presence of multivariate non-normality, this study used partial least squares structural equation modeling (PLS-SEM) to test the associations hypothesized (90). Moreover, artificial neural network analysis has been deployed for a model-free estimation using parallel, multilayer, and non-linear regression analyses. According to standard practices of performing dual-stage analysis, at first, PLS-SEM is used to determine the important exogenous factors, which are subsequently used as the input neurons for the artificial neural network (ANN) analysis to entirely appreciate the non-linearity amongst the endogenous and exogenous factors (91).

## Findings

### Demographic Characteristics

It was found that more than half of the respondents (52%) were women, whilst 48% were male respondents. The majority of the respondents (46.5%) were 21–25 years old, followed by 25.5% below 21 years, 9.4% were in the ages ranging 26–30 years, and 4.8% were 31–35 years old. According to a CDC report (15) the COVID-19 virus has hospitalized and killed a considerable number of individuals in these age groups until now. In terms of ethnicity, most of the respondents were Chinese (80.5%), others (12.8%), Malays (4.7%), and Indians (2.0%). Most of the respondents dwelled in urban areas (88.3%), whilst only 11.7% resided in rural areas. In this survey, 83.7% of the respondents were single, 15.1% were married, and 0.7% claimed to be divorcees. The education background showed that more than half of the respondents had Bachelor's degree (55.2%), followed by 20.8% with a diploma/technical school certificate, 19.7% with a secondary school certificate, 3.1% with a master's degre, and 1.3% with a doctoral degree. Most of the respondents earned a monthly income of below RM2,500 (57.5%), followed by 26.8% of RM2,501–RM5,000, 3.8% of RM5,001–RM7,500, 2.4% of above RM12,500, and 1.3% of RM10,000–RM12,500. Around 23.3% of the respondents had occasionally purchased travel insurance, whilst 21.6% had never purchased travel insurance, 21.3% had rarely purchased travel insurance, followed by 16.1% who very rarely, 11.1% who always, and 6.6% who very frequently purchased travel insurance.

### Reliability and Validity

This study estimated the mean score by measuring the central tendency in the form of an average value. Standard deviation estimates the span of observed values. The larger the standard deviation, the more spread out the observations. Cronbach's alpha (CA) and composite reliability (CR) were employed to evaluate the internal consistency of the items. The findings revealed that 0.756 was the lowest CA value, signifying that all alpha values exceeded the threshold value of 0.70 (92, 93). As the lowest Dillon-Goldstein's *rho* (DG *rho*) value was 0.766, it showed that all rho scores were greater than the recommended score of 0.70 (94), thus indicating the suitability of CR. The CR values of latent variables that exceeded the threshold value of 0.70 (92) signified the internal consistency of the variables. The average variance extracted (AVE) score of all latent variables was above 0.50, which demonstrated the convergent validity. The VIF scores, which were below 3 and lower than the threshold value recommended by Diamantopoulos and Siguaw (95), indicated the absence of multicollinearity issues in this study. The findings tabulated in Table 2 exemplify that the measurement model has good reliability and convergent validity.

**Table 2:** Reliability and validity.

**Variables**	**No. items**	**Mean**	**SD**	**CA**	**DG *rho***	**CR**	**AVE**	**VIF**
IL	4	3.894	0.682	0.762	0.766	0.849	0.586	1.362
HR	5	3.856	0.673	0.756	0.766	0.836	0.506	1.420
HC	5	3.856	0.687	0.828	0.831	0.879	0.592	1.269
AT	5	3.675	0.887	0.906	0.906	0.930	0.726	1.902
PR	4	3.670	0.849	0.853	0.886	0.900	0.693	1.039
SN	5	3.422	0.911	0.908	0.910	0.931	0.731	2.207
PB	5	3.414	0.913	0.880	0.885	0.913	0.679	1.456
WTI	5	3.004	1.193	0.903	0.920	0.929	0.724	1.011
INC	1	1.720	1.113	1.000	1.000	1.000	1.000	1.005
PTI	1	3.110	1.584	1.000	1.000	1.000	1.000	-

*IL, insurance literacy; HR, perceived health risk; HC, health consciousness; AT, attitude toward travel insurance; PR, perceived product risk; SN, subjective norms; PB, perceived behavioral control; WTI, willingness to purchase of travel insurance; INC, average monthly income; PTI, purchase travel insurance*.

The Fornel–Larcker criterion shows (presented in Appendix 2) that all the square root of each construct's (see Supplementary Material - Data Sheet 2) AVE is greater than the correlations with other latent constructs, confirming discriminant validity (96). Appendix 3 (see Supplementary Material - Data Sheet 2) shows that each construct has high loading on its construct, and low loading on the other constructs (97). As a result, all items on loading were high on their constructs, which reflected the suitability of discriminant validity. The heterotrait–monotrait (HTMT) ratio (presented in Appendix 2) that all the square root of each construct's (see Supplementary Material - Data Sheet 2) was lower than 0.90, which indicated that the construct was conceptually distinct and discriminate validity was achieved (98).

### Hypothesis Testing

The *r*^2^ value of 0.239 indicates that around 24% of the variation in AT can be explained by IL, HR, and perceived HC. The *f*^2^ values indicate that the effect of IL (0.064), HR (0.009), and perceived HC (0.070) on AT is relatively low. The *r*^2^ value of 0.617 indicates that around 62% of the variation in WTI can be explained by AT, PR, SN, and PB. The *f*^2^ values indicate that the effect of AT (0.312) is relatively high, whereas the effect of PR (0.001), SN (0.081), and PB (0.049) on WTI is relatively low. Finally, the *r*^2^ value of 0.239 indicates that around 24% of the variation in the PTI can be explained by WTI. The *f*^2^ value of 0.236 indicates a medium effect of WTI on the PTI amongst the working adults in Malaysia.

The structural model was applied to estimate the interrelations amongst the constructs embedded in the proposed research framework. Table 3 portrays the path coefficients and hypotheses results of the structural model. The supporting form of the hypothesis is that the *p*-value should be < 0.05. As a result, IL, HR, and perceived HC disclosed a significant effect on AT, which indicated the acceptance of hypotheses H_1_, H_2_, and H_3_. WTI was attributed to AT, SN, and PB, which supported hypotheses H_4_, H_6_, and H_7_. However, PR had no significant effect on WTI, which was inconsistent with hypothesis H_5_. Therefore, PR was not a significant predictor of WTI in the model. WTI exerted a significantly positive impact on the actual PTI, which supported hypothesis H_8_.

**Table 3:** Path coefficients.

**Hypo**		**Beta**	**CI - Min**	**CI - Max**	***t***	***p***	***r* ^2^**	***f^**2**^***	**Q^**2**^**	**Decision**
*Determinants of AT*				
H_1_	IL → AT	0.257	0.202	0.319	7.365	0.000		0.064		Accept
H_2_	HR → AT	0.099	0.047	0.156	2.859	0.002	0.239	0.009	0.172	Accept
H_3_	HC → AT	0.260	0.207	0.316	7.758	0.000		0.070		Accept
*Determinants of WTI*				
H_4_	AT → WTI	0.477	0.419	0.527	14.434	0.000		0.312		Accept
H_5_	PR → WTI	0.019	−0.015	0.055	0.922	0.178	0.617	0.001	0.438	Reject
H_6_	SN → WTI	0.261	0.200	0.320	7.165	0.000		0.081		Accept
H_7_	PB → WTI	0.166	0.122	0.217	5.611	0.000		0.049		Accept
*Determinants of PTI*				
H_8_	WTI → PTI	0.444	0.403	0.480	19.000	0.000	0.239	0.257	0.236	Accept

*IL, insurance literacy; HR, perceived health risk; HC, health consciousness; AT, attitude toward travel insurance; PR, perceived product risk; SN, subjective norms; PB, perceived behavioral control; WTI, willingness to purchase travel insurance; INC, average monthly income; PTI, purchase of travel insurance*.

### Moderation and Mediation

Table 4 presents the summarized results of moderating and mediating effects. The outcomes revealed that monthly income moderated the effect of WTI on the actual PTI policy amongst working adults in Malaysia, which supports hypothesis H_9_. Hence, monthly income displayed a moderation effect in the proposed model. Next, AT mediated the effect of IL, HR, and perceived HC on WTI, thus supporting hypotheses H_10A_, H_10B_, and H_10C_. WTI mediated the effect of AT, SN, and PB on PTI but did not display any mediation effect on the relationship between PR and PTI. Thus, hypotheses H_11A_, H_11C_, and H_11D_ are supported, whilst hypothesis H_11B_ is rejected.

**Table 4:** Moderating and mediating effects.

**Hypo**	**Associations**	**Beta**	**CI - Min**	**CI - Max**	***t***	***p***	**Decision**
*Moderating Effect of Income*
	INC → PTI	0.185	0.138	0.226	6.715	0.000	Moderation
H_9_	INC*WTI → PHI	−0.151	−0.117	0.202	4.036	0.000	
*Mediating Effect of AT*
H_10A_	IL → AT → WTI	0.123	0.123	0.020	6.213	0.000	Accept
H_10B_	HR → AT → WTI	0.047	0.048	0.017	2.854	0.002	Accept
H_10C_	HC → AT → WTI	0.124	0.124	0.018	6.820	0.000	Accept
*Mediating Effect of WTI*
H_11A_	AT → WTI → PTI	0.212	0.210	0.019	11.145	0.000	Accept
H_11B_	PR → WTI → PTI	0.009	0.009	0.009	0.925	0.178	Reject
H_11C_	SN → WTI → PTI	0.116	0.116	0.017	6.862	0.000	Accept
H_11D_	PB → WTI → PTI	0.074	0.075	0.014	5.223	0.000	Accept

*IL, insurance literacy; HR, perceived health risk; HC, health consciousness; AT, attitude toward travel insurance; PR, perceived product risk; SN, subjective norms; PB, perceived behavioral control; WTI, willingness to purchase travel insurance; INC, average monthly income; PTI, purchase of travel insurance*.

#### Importance–Performance Matrix Analysis (IPMA)

**Figure 2:**
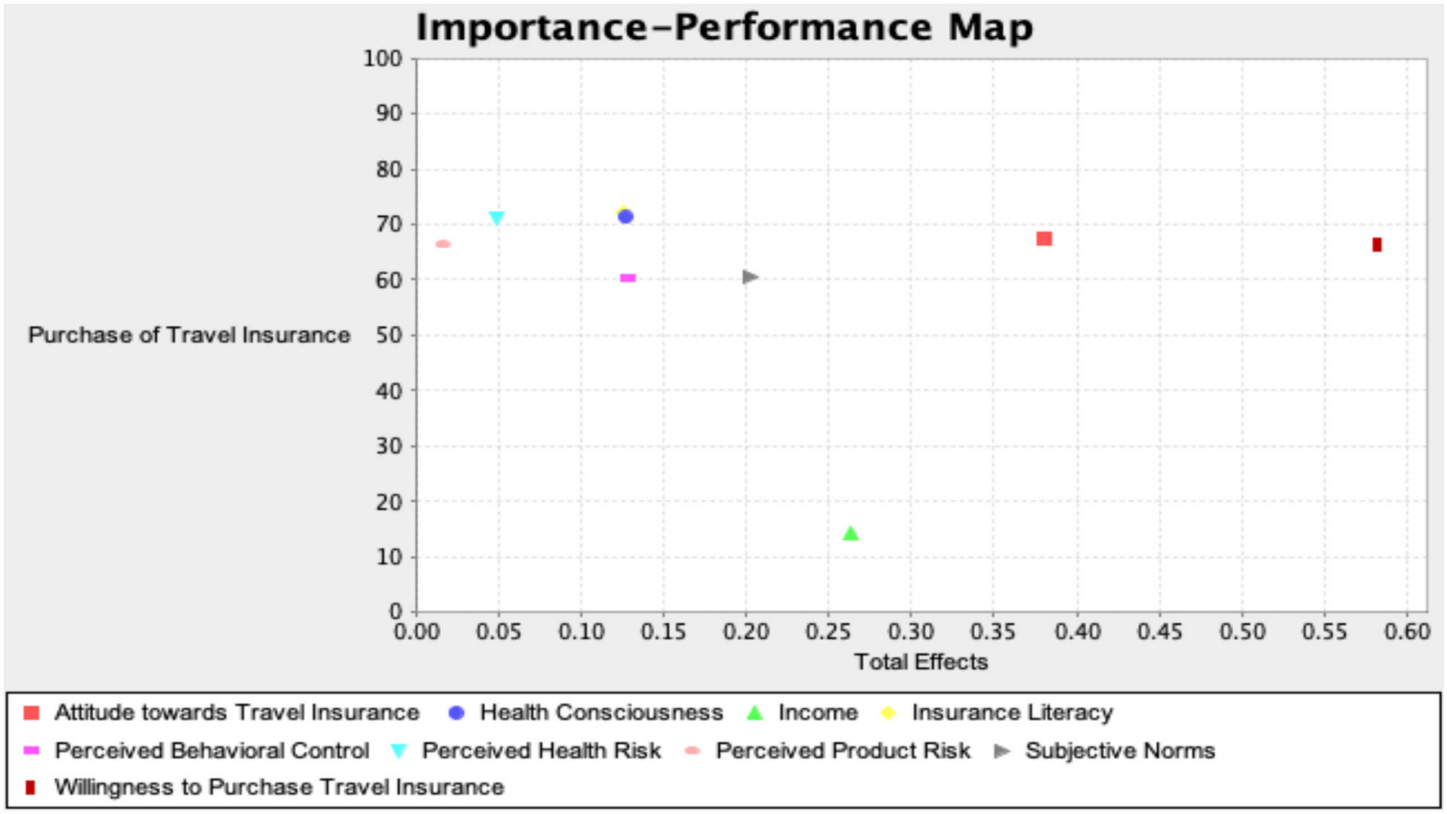
Importance–performance matrix analysis.

The importance–performance matrix analysis (IPMA) extends the results of PLS-SEM by also taking the performance of each construct into account. As a result, conclusions can be drawn on two dimensions (i.e., both importance and performance), which is particularly important to prioritize managerial actions (99–101). Hence, the IPMA was performed *via* PLS to determine the robustness of the study outcomes by accounting for the performance of each component actions (101). The results (as shown in Figure 2) revealed that the WTI has the highest effect on the purchase of travel insurance, followed by AT, average monthly income, SN, PB, and HC.

## Discussion and Conclusion

This study looked into the willingness of Malaysian working adults to purchase travel insurance and the key influential factors of PTI schemes within the Malaysian context. Essentially, this study has extended the TPB model by including the psychological dimensions of IL, HR, PR, and HC. This study has broadened the TPB application in the travel insurance research domain. The TPB, despite its vast application in human behavior studies, is scarcely applied in the travel insurance context. The extended TPB model applied in this study effectively clarified the working adults' WTI policy and the actual purchase. Overall, the findings suggest that most of the working adults in Malaysia displayed a positive attitude and willingness toward purchasing travel insurance. The finding of hypothesis H_1_ indicates that IL emerged as a significant influential factor for AT schemes. Similarly, James et al. (31), who had explored health IL amongst college students, reported that higher IL led to a strong attitude toward PTI.

HR and perceived HC are integral components that influence the attitude of Malaysian working adults toward travel insurance. The findings of hypothesis H_2_ support the result reported by early scholars [e.g., (102)], who found that HR had a positive influence on behavioral intention. They further indicated that the low-income index was associated with HR. Next, hypothesis H_3_ supports the notion that the working adults' HC positively influenced their AT. This finding is in agreement with Hoque et al. (42), who reported that HC had positively signified consumers' beliefs. Working adults with greater HC demonstrated a stronger AT. This exemplified that the consumers' AT was predicted by HC. Hence, an attitude toward purchasing e-travel insurance is highly required to increase a consumer's HR and perceived HC.

WTI is an extensive process influenced by the AT (hypothesis H_4_), SN (hypothesis H_6_), and PB (hypothesis H_7_). These findings are in line with those reported in the previous studies [see (72, 103)] on the perceived behavior of consumers. This demonstrated that the willingness of working adults to purchase travel insurance should emphasize on AT, SN, and PB. PR displayed an insignificant relationship with WTI (hypothesis H_5_). This is ascribed to the fact that Malaysian working adults are more conscious of HR and PB, rather than the PR of purchasing travel insurance schemes (104). In a similar vein, Bonnin (52) identified the relationship between the PR of purchasing a product and the attractiveness of online stores. Meanwhile, Suki and Suki (51) revealed that PR had a negative influence on perceptions. Hypothesis H_8_, which denotes the WTI, exhibited a significant influence on the PTI amongst working adults in Malaysia. This finding is in agreement with that reported by Masud et al. (105), who explained that one's willingness to purchase is an effective component that can be used to predict the actual purchase behavior of products and services.

This study reports that monthly income moderated the relationship between WTI and the actual purchase amongst Malaysian working adults. This exemplified that those with higher income had a better understanding of their WTI. Turning to contributions, this study has unraveled the significant impacts of AT and WTI amongst working adults in Malaysia. Attitudes toward travel insurance mediated the influence of IL, HR, and HC on WTI. On the other hand, WTI mediated the influence of attitude, SN, and PB on the actual PTI. These crucial findings portray that, if working adults have travel IL, HR, and HC, they are more willing to purchase travel insurance products and services. Similarly, AT, SN, and PB emerged as the important factors for working adults to be willing to purchase travel insurance. These outcomes are in line with those reported in prior studies on the perceived risk and willingness to pay the premium price by Casidy and Wymer (54) and Chen (43) about the need to account for perceived HC upon assessing the influence of WTI. The results showed that WTI did not mediate the correlation between PR and PTI. It established that WTI did not influence PR. This is attributed to the fact that working adults in Malaysia are more conscious about their health (106) rather than PR of travel insurance.

This study offers an in-depth understanding of the working adults' behavior in their travel insurance purchase decision, thus contributing to the TPB and PTI policy domains. This study extends the theoretical contributions of IL and perceived health-related risk-reduction strategy. Apart from minimizing the HR, purchasing travel insurance displays escalated competence. The study outcomes are valuable for those involved in sales of travel insurance, mainly because the attitude of working adults toward travel insurance and their WTI aids the decision-making process. This can help marketers to better understand the effects of IL and HR on AT, which leads to consequential behavior and PTI schemes. The model of this study can be implemented in both developed and developing countries. This study argues that, although income level plays a crucial role in influencing travel insurance purchase behavior, more research is required to determine if the travel insurance purchase behavior of individuals differs in other countries, particularly in low- and middle-income countries.

This study sheds light on the travel insurance purchase behavior and the role that it has in the HR strategies. Nevertheless, this study poses several limitations regarding the generalisability of the outcomes due to the nature of the quantitative study. Future investigations may adopt both qualitative and quantitative methods to assess respondents from different backgrounds to verify the findings reported in this present study. This study had only concentrated on the health-related risk perceptions and TPB to measure the willingness of Malaysian working adults to purchase travel insurance. Further study may explore other human psychological related attributes, HR reduction, personal values, self-respect, and benefits of travel insurance to determine if these factors are in line with different demographic variables. Besides, the majority of the participants in this study are Chinese, which could raise the representative issues in the study. However, the Commercial Divisions of Malaysia Airports implemented some new strategic initiatives in 2016, including a campaign to attract more Chinese tourists, which could be one of the reasons for the high number of Chinese respondents in this study. However, policymakers should carefully interpret the findings, and future research should examine if other ethnic groups react similarly to travel insurance purchasing behavior.

## Data Availability Statement

The original contributions presented in the study are included in the article/Supplementary Material, further inquiries can be directed to the corresponding author/s.

## Ethics Statement

Ethical review and approval was not required for the study on human participants in accordance with the local legislation and institutional requirements. The patients/participants provided their written informed consent to participate in this study.

## Author Contributions

MR, QY, AS, and SF: conceptualization, instrument, data collection, and writing-original draft. AA and TJ: conceptualization, formal analysis, and writing–revisions. All authors contributed to the article and approved the submitted version.

## Conflict of Interest

The authors declare that the research was conducted in the absence of any commercial or financial relationships that could be construed as a potential conflict of interest.

## Publisher's Note

All claims expressed in this article are solely those of the authors and do not necessarily represent those of their affiliated organizations, or those of the publisher, the editors and the reviewers. Any product that may be evaluated in this article, or claim that may be made by its manufacturer, is not guaranteed or endorsed by the publisher.
