# Supplementary material for: Predicting the Willingness and Purchase of Travel Insurance During the COVID-19 Pandemic
Source: Front Public Health. 2022 Jul 4;10:907005. doi: 10.3389/fpubh.2022.907005 (PMC9291636; doi:10.3389/fpubh.2022.907005)
Supplement: Supplementary file 1 [file Data_Sheet_1.PDF]

DATA - Travel Insurance

Gender	Age	Ethnicity	Marital_S	Education	Income	Employment	Living_A	IL1	IL2	IL3	IL4	HR1	HR2	HR3	HR4	HR5	HC1	HC2	HC3	HC4	HC5	AT1	AT2	AT3	AT4	AT5	PR1	PR2	PR3	PR4	PR5	SN1	SN2	SN3	SN4	SN5	PB1	PB2	PB3	PB4	PB5	WHI1	WHI2	WHI3	WHI4	WHI6	PTI
1	2	2	1	3	1	1	1	3	4	4	3	4	4	4	4	3	4	5	4	4	4	3	4	4	4	3	1	4	2	4	1	4	3	4	2	4	3	4	3	4	1	3	3	5	4	5	2
2	2	4	1	1	1	1	1	3	3	5	3	3	4	5	2	3	4	5	3	3	3	5	5	5	3	5	1	3	3	4	1	1	3	3	1	1	2	1	1	1	1	6	7	4	7	7	1
1	9	2	2	4	4	2	1	5	5	5	5	5	4	5	5	5	4	5	5	4	5	1	1	1	1	1	1	3	3	3	1	1	1	1	1	1	1	5	5	5	5	3	4	3	3	3	1
2	2	4	1	3	1	1	1	3	2	2	2	2	5	5	5	5	5	5	4	4	4	2	5	4	5	5	1	1	5	1	1	5	4	2	4	4	3	3	4	4	3	5	4	2	3	2	3
1	1	2	1	1	1	1	1	5	5	5	5	5	5	5	5	5	5	5	5	5	5	5	5	5	5	5	1	1	1	1	1	4	5	4	5	4	5	5	5	5	5	7	7	7	7	7	6
1	2	2	1	3	1	3	1	5	5	5	5	5	5	3	2	1	5	5	5	3	5	5	5	5	5	5	1	1	1	1	1	3	3	5	5	5	3	1	3	3	5	7	7	4	6	6	6
2	2	1	1	3	1	2	1	4	5	3	5	5	5	4	2	4	5	5	5	5	5	4	4	5	3	4	1	1	1	1	1	5	5	5	4	4	3	3	3	5	5	7	7	5	7	7	4
1	8	4	2	1	3	3	1	3	5	4	4	4	3	4	4	4	2	4	4	5	5	5	5	5	5	5	1	1	1	1	1	5	5	5	5	5	5	5	5	5	5	7	7	7	7	7	6
1	2	4	1	3	3	2	1	5	3	5	2	2	3	4	5	4	5	5	5	4	3	4	4	3	4	4	1	1	1	1	1	5	5	5	4	1	3	3	3	2	1	3	2	6	7	6	3
1	1	2	1	3	1	1	1	3	2	4	2	3	2	4	3	2	5	1	3	1	1	2	1	2	3	1	1	1	1	1	2	1	1	1	1	1	5	5	5	5	5	1	1	1	1	1	1
2	2	2	1	3	1	2	1	5	4	5	3	4	4	5	3	4	4	4	5	3	4	3	3	3	2	3	1	2	2	4	1	3	3	3	2	2	4	2	2	2	3	4	3	3	4	3	3
1	2	2	1	1	1	1	1	5	5	5	3	2	5	5	2	2	2	1	2	1	2	2	2	2	2	2	1	1	2	5	1	2	1	1	1	1	1	1	1	4	1	3	2	1	2	3	2
2	2	2	1	3	1	1	1	5	5	5	5	5	5	5	1	1	5	5	5	1	5	3	3	3	3	3	1	1	1	1	1	1	1	1	1	1	1	1	1	1	5	1	7	1	1	1	1
1	2	2	1	3	1	1	2	5	5	5	4	4	5	5	3	2	4	4	5	4	5	4	4	4	4	5	1	2	2	4	1	2	3	3	3	3	3	5	5	5	5	6	6	6	6	3	4
2	1	2	1	3	1	2	1	5	5	5	5	5	5	5	5	5	5	5	5	5	5	5	5	5	5	5	1	2	2	1	1	2	5	5	5	5	5	5	5	5	5	7	5	7	7	7	6
1	2	4	1	1	3	3	1	4	3	2	4	5	4	4	2	4	2	3	4	4	4	2	3	3	2	2	1	4	3	3	1	1	2	2	5	5	2	1	1	1	1	5	7	5	5	5	1
2	2	3	1	3	1	1	1	5	5	3	5	5	5	5	3	4	1	3	3	5	3	5	5	5	5	5	1	5	4	5	1	2	4	5	4	3	3	3	3	3	4	3	4	6	4	5	3
2	1	2	1	1	1	3	1	4	2	4	3	4	5	5	5	5	4	4	5	3	4	4	4	4	5	5	1	1	1	3	1	3	3	4	4	4	4	2	3	3	3	6	6	6	6	6	6
1	1	2	1	3	1	3	1	5	5	4	3	5	5	5	5	5	4	3	4	3	3	4	4	4	3	2	1	1	1	1	1	4	4	4	4	4	4	5	4	5	4	5	7	5	6	6	4
2	2	2	1	2	1	2	1	5	5	5	5	5	5	5	5	5	5	5	5	3	5	5	5	5	5	5	1	1	1	1	1	5	5	5	5	5	1	5	5	5	5	7	7	7	7	7	6
1	4	2	1	2	6	2	1	5	5	5	5	5	5	5	5	5	3	5	4	4	5	5	5	5	5	5	1	1	1	1	2	5	5	5	5	5	1	5	5	5	5	7	7	7	7	7	6
2	3	2	1	3	3	3	1	3	4	5	3	5	5	5	1	5	4	4	5	3	5	5	5	5	5	5	1	1	1	1	2	5	5	5	3	3	5	5	5	5	5	7	7	7	7	7	6
2	1	4	1	3	1	1	1	5	5	2	3	5	5	5	4	3	2	2	2	1	3	4	3	2	1	1	1	2	1	5	2	1	1	1	4	4	1	1	1	1	1	2	7	3	1	1	1
2	2	3	1	2	1	1	1	4	4	5	3	2	2	2	3	3	4	5	5	3	5	3	3	3	3	3	2	5	5	4	2	1	3	3	1	1	2	3	2	4	5	5	7	5	5	5	1
2	2	2	1	3	1	3	1	3	5	3	4	4	4	2	4	4	5	5	5	4	5	2	2	2	1	2	2	4	5	3	2	1	1	1	1	1	1	1	1	1	4	2	6	1	1	1	1
1	1	2	1	1	1	1	1	3	4	3	3	2	4	2	3	1	5	2	3	1	3	5	5	4	3	3	2	1	5	2	1	3	2	5	4	4	2	2	3	4	2	6	4	3	4	3	4
2	1	2	1	2	3	3	1	4	3	5	4	2	5	5	2	3	4	4	2	5	2	5	5	5	4	4	2	5	3	4	1	5	4	5	5	4	4	4	3	4	3	4	7	4	5	5	3
2	2	2	1	1	3	4	1	3	5	5	4	4	3	4	1	3	3	4	3	3	4	4	4	3	3	3	2	1	1	2	2	3	3	3	2	2	3	3	2	4	2	4	6	3	4	4	4
1	2	2	1	1	1	1	1	3	4	5	3	5	3	4	5	3	3	3	4	5	5	4	2	4	3	3	2	1	2	2	2	4	5	4	4	3	2	4	4	5	4	4	6	4	6	4	6
2	1	2	1	1	1	3	1	4	4	2	4	3	3	4	3	3	5	4	5	5	5	5	5	5	4	3	2	1	2	2	2	4	4	4	3	3	2	3	5	3	3	7	6	7	7	7	4
2	6	4	3	3	2	3	1	5	5	5	2	5	3	5	5	5	5	4	5	5	5	5	5	5	5	5	2	1	2	2	2	5	5	5	4	4	4	4	4	4	4	7	7	5	7	7	6
1	2	2	1	3	1	1	1	4	4	5	4	5	5	5	5	3	3	3	4	3	2	4	4	4	3	3	2	1	2	2	2	3	5	5	1	1	1	1	1	2	2	2	7	5	5	5	3
1	1	2	1	2	1	3	2	5	4	3	5	4	4	4	2	3	5	3	5	3	5	5	5	5	5	5	2	1	2	2	2	5	5	5	5	4	3	3	3	3	3	7	7	7	7	7	5
1	2	2	1	3	1	2	1	1	1	1	1	3	4	4	5	2	1	2	5	3	4	1	1	1	1	1	2	1	2	2	2	3	3	2	1	1	5	5	5	5	4	1	1	1	1	1	2
2	2	2	1	3	1	3	1	2	2	2	2	1	3	5	4	3	4	4	5	1	5	1	1	1	1	3	2	1	2	2	2	2	1	1	1	1	3	3	1	1	4	1	2	1	1	1	1
2	2	4	1	2	4	3	1	5	5	5	1	5	5	5	3	3	2	4	3	4	2	4	4	4	1	1	2	1	2	2	2	1	1	1	1	1	1	2	1	2	4	1	1	1	1	1	1
1	6	4	2	3	6	2	2	5	5	5	5	5	5	5	5	1	5	5	5	5	5	5	5	5	1	1	2	1	2	2	2	1	1	1	1	1	5	1	5	5	5	7	7	7	7	7	6
2	2	2	1	3	1	1	2	4	4	3	4	4	4	4	2	2	3	3	3	4	4	3	3	3	1	1	2	1	2	2	2	2	2	2	3	3	2	1	2	3	2	4	4	3	3	3	1
1	2	3	1	3	1	1	1	1	1	1	1	1	1	1	1	1	1	2	1	1	2	1	1	2	1	1	2	1	2	2	2	1	1	1	1	1	1	1	2	1	2	1	2	1	2	2	1
2	1	4	1	3	2	3	1	4	3	3	3	4	4	5	4	4	4	2	3	3	4	4	4	4	3	3	2	2	4	4	2	3	4	2	2	3	3	3	3	3	4	5	4	5	4	4	4
2	3	2	1	3	2	3	1	3	3	3	3	4	4	3	3	3	4	4	4	3	4	3	3	3	2	2	2	2	4	4	2	2	2	2	2	2	3	4	3	3	3	4	3	3	3	3	2
1	2	2	1	3	2	3	1	2	3	5	3	4	5	4	3	4	3	3	3	3	3	3	2	3	2	2	2	2	4	2	2	2	2	2	1	1	5	5	4	5	5	3	2	3	2	2	2
2	1	2	1	3	1	1	1	4	5	3	4	5	5	3	3	2	4	4	4	4	4	4	4	4	4	4	2	2	3	4	2	2	3	3	2	2	1	1	2	2	4	5	7	6	6	6	1
1	2	2	1	3	1	1	1	5	4	5	5	4	4	3	3	5	3	3	4	3	3	3	3	3	3	3	2	2	3	3	2	3	3	3	3	3	4	3	3	3	3	4	5	5	5	5	1
2	3	2	1	3	2	3	1	4	3	5	4	5	5	4	4	4	4	4	4	3	3	4	4	4	3	2	2	2	2	2	2	2	2	2	3	3	4	5	4	4	4	3	2	2	2	2	4
2	1	2	1	3	1	1	2	3	3	3	3	4	3	3	2	2	5	5	4	5	4	5	3	3	4	2	2	3	4	5	2	2	2	4	2	2	3	1	1	4	5	6	6	3	4	5	3
2	7	4	3	1	3	2	1	5	5	4	2	2	4	2	1	3	4	2	4	3	2	2	2	2	1	1	2	3	4	3	2	4	3	3	2	2	3	4	4	4	4	3	4	6	3	3	4
1	2	2	1	3	1	1	1	5	5	3	3	3	2	5	4	4	4	4	4	4	3	4	4	4	3	3	2	3	3	3	2	2	2	2	3	3	2	2	2	2	2	4	4	4	4	5	3
1	1	4	1	1	1	1	1	4	3	5	4	3	3	3	2	2	2	3	4	3	2	2	4	4	2	3	2	3	3	3	2	1	1	1	1	1	1	1	1	1	1	3	5	3	3	3	4
1	2	2	1	3	1	1	2	4	4	4	4	4	4	3	2	2	3	4	4	3	4	2	2	3	3	2	2	3	3	2	2	2	2	2	2	2	2	2	2	2	2	2	3	2	2	2	2
1	2	2	1	2	1	3	1	4	3	4	3	2	4	4	2	3	3	4	2	2	3	4	3	3	4	4	2	3	3	2	2	3	3	2	2	3	2	2	3	2	2	5	4	4	4	5	6
2	1	2	1	3	1	5	1	5	5	5	5	5	5	5	5	5	4	3	3	1	1	3	2	5	3	4	2	4	5	4	2	2	5	4	1	4	2	5	2	4	5	1	2	3	2	3	1
1	4	2	2	3	3	3	1	4	3	5	4	3	4	5	3	4	3	4	3	4	5	3	4	5	4	3	2	4	3	4	2	3	4	5	4	3	5	4	4	3	5	6	5	6	4	5	4
2	1	2	1	3	1	4	1	5	5	5	4	4	5	5	4	4	4	4	4	4	4	5	4	5	4	2	2	4	2	3	2	4	2	4	2	5	2	4	2	4	2	6	4	3	5	5	2
2	2	2	1	3	1	3	1	2	1	1	5	5	5	5	2	5	4	2	5	2	5	2	4	2	2	4	2	5	5	5	2	2	2	4	2	2	4	1	1	5	3	4	5	2	5	2	4
1	2	2	1	2	1	2	1	4	5	3	5	2	3	2	1	4	5	5	5	3	4	4	3	3	5	4	2	5	4	4	2	4	2	3	3	2	2	4	3	5	4	4	5	4	3	5	1
1	3	1	1	5	6	4	1	5	5	5	3	3	4	4	3	3	1	4	3	3	4	4	4	5	4	5	2	2	5	4	1	5	1	1	4	3	3	5	2	2	3	1	3	7	6	1	6
1	3	2	1	3	3	3	1	2	4	4	4	4	4	4	5	5	3	2	3	4	4	2	2	3	1	2	2	2	5	4	1	3	2	2	3	3	2	4	2	3	2	3	5	3	3	3	2
1	3	2	1	3	2	3	1	5	5	5	4	5	5	4	4	4	4	4	3	4	3	4	3	4	3	3	2	2	4	5	1	3	3	3	3	3	3	3	4	4	4	5	7	5	5	5	1
2	2	2	1	3	1	1	1	5	5	3	3	5	5	3	2	3	4	4	4	3	4	5	5	5	3	2	2	2	4	5	1	3	3	3	3	3	1	1	1	3	1	6	5	2	6	6	2
2	3	2	1	3	2	3	1	4	3	3	4	4	4	4	2	2	4	4	3	2	4	4	4	4	4	4	2	2	4	4	1	3	3	4	2	2	2	2	2	3	4	5	5	3	5	5	2
2	1	2	1	3	1	1	1	4	3	4	5	5	4	5	5	5	4	3	5	4	4	5	5	5	3	4	2	2	4	4	1	3	4	5	3	3	4	4	4	4	3	5	4	5	7	6	3
1	2	2	1	3	2	3	1	4	4	4	4	3	3	4	4	3	4	3	4	3	3	4	3	3	3	2	2	2	3	4	1	3	4	3	3	4	3	3	3	3	3	5	3	3	3	3	1
2	2	2	1	3	1	1	1	4	4	4	5	4	4	5	3	3	4	3	3	3	3	3	3	3	3	4	2	2	3	4	1	3	3	2	2	2	2	2	2	2	3	4	5	4	5	5	2
1	2	2	1	3	2	3	1	5	5	4	5	4	4	5	4	4	5	5	5	4	4	4	4	4	3	3	2	2	3	3	1	3	3	3	3	3	4	3	3	3	4	5	5	4	4	5	3
1	9	2	2	4	5	3	1	5	5	5	5	5	5	5	4	4	4	4	5	5	5	4	4	4	3	3	2	2	3	3	1	3	4	4	4	4	5	5	5	5	5	7	6	6	7	7	6
2	2	4	1	3	1	1	2	4	4	5	4	5	5	3	4	3	3	3	4	3	3	4	4	4	3	4	2	2	3	3	2	3	3	3	3	3	2	2	2	2	2	4	5	4	3	3	1
2	2	2	1	3	1	4	1	5	4	5	3	5	4	4	2	3	3	4	5	3	3	5	5	5	3	5	2	2	3	2	2	3	3	4	4	4	5	4	4	4	4	6	5	5	5	5	4
2	2	2	1	3	1	4	1	5	5	4	5	5	5	5	5	5	4	4	4	4	4	3	3	4	2	3	2	2	2	4	2	3	3	3	3	3	2	3	2	3	4	4	3	3	4	3	2
2	2	2	1	3	6	3	1	4	4	4	4	3	3	4	2	2	2	3	3	3	3	4	4	4	3	4	2	2	2	4	2	3	3	3	3	3	3	3	3	3	3	6	6	6	6	6	4
2	3	2	1	3	2	1	1	4	3	2	4	4	4	4	4	4	3	3	4	2	4	3	2	2	2	2	2	2	2	3	2	2	4	3	2	3	4	3	2	4	3	4	5	6	4	3	2
1	2	2	1	3	2	3	1	5	5	5	5	5	5	5	5	5	4	4	4	3	4	4	4	4	3	4	2	2	2	2	1	3	3	3	3	3	3	4	4	4	4	5	5	6	6	6	3
2	1	2	1	3	1	1	1	4	4	5	4	5	5	5	3	3	5	5	3	5	3	5	5	5	3	3	2	2	2	2	1	3	2	2	2	2	4	3	3	2	3	6	6	6	7	7	5
1	2	2	1	5	1	4	1	4	4	4	3	4	4	4	3	2	3	4	4	4	4	5	4	5	4	5	2	2	2	2	1	4	4	4	3	3	4	3	5	3	2	6	5	6	6	6	5
2	8	2	2	2	1	3	1	4	4	4	3	5	5	5	5	5	4	3	4	3	3	4	3	4	3	2	2	2	2	2	1	3	3	3	3	3	3	3	2	2	4	4	7	5	4	4	3
1	3	2	1	2	2	3	1	3	4	4	3	4	4	4	4	4	4	4	4	4	4	3	3	3	3	3	2	2	2	2	1	3	3	3	3	3	3	3	3	3	3	4	5	4	5	5	4
2	5	2	2	2	1	4	1	3	3	4	3	2	4	2	2	4	3	3	2	3	3	2	4	3	4	3	2	2	2	2	1	2	2	2	4	4	4	3	3	2	3	5	4	3	3	5	3
2	1	2	1	3	1	1	1	5	5	4	3	4	5	4	2	2	3	3	3	3	3	5	5	5	5	5	2	2	1	3	1	4	4	5	5	5	3	1	4	2	2	7	7	5	6	6	4
1	4	2	1	3	4	2	1	1	1	1	3	4	4	3	4	2	4	3	5	4	4	3	3	3	3	3	2	2	1	2	1	1	2	2	2	2	5	5	5	5	5	5	6	6	5	3	3
1	1	1	1	2	1	4	2	4	4	2	5	2	5	4	5	4	3	3	4	4	5	2	3	5	3	4	2	3	5	3	1	4	3	5	5	5	4	3	1	5	4	6	6	4	7	7	1
2	1	1	1	3	1	1	1	4	4	5	5	5	5	5	3	4	4	4	5	2	5	5	5	5	4	3	2	3	4	5	1	4	4	4	5	5	3	5	4	5	5	7	7	3	7	7	4
1	1	2	1	3	2	3	1	4	3	4	3	5	3	3	4	3	3	3	5	4	5	4	3	3	4	3	2	3	4	5	2	3	3	4	2	3	4	3	2	4	3	5	6	6	7	5	4
2	2	4	1	1	1	1	1	5	4	5	3	5	3	5	4	4	3	3	4	3	4	5	5	5	2	5	2	3	4	4	2	4	4	4	4	4	3	3	2	3	4	7	7	5	7	7	4
2	2	2	1	3	1	1	1	3	4	3	5	4	4	4	4	3	4	3	3	3	3	4	3	4	3	3	2	3	4	4	2	3	4	3	2	2	3	3	3	3	3	4	6	5	5	5	2
2	2	2	1	3	2	3	1	3	3	5	4	3	3	2	3	3	3	2	3	3	3	5	5	5	5	5	2	3	4	3	2	3	3	3	3	3	3	3	3	3	3	4	4	4	5	5	4
2	1	2	1	1	1	4	1	3	4	3	2	3	4	4	3	3	4	3	4	5	3	3	2	3	2	2	2	3	3	4	2	2	2	2	1	1	3	2	3	2	2	4	5	4	3	4	4
1	2	2	1	2	1	3	1	4	4	5	3	4	3	3	2	3	4	5	4	3	4	3	3	3	3	3	2	3	3	3	1	3	2	2	2	2	3	3	3	3	5	4	4	4	3	4	1
2	1	2	1	3	1	1	1	3	4	4	4	4	4	4	4	3	4	4	4	4	4	4	2	3	2	2	2	3	3	3	1	3	4	4	3	4	3	3	3	4	3	4	4	3	3	4	1
1	1	1	1	2	1	1	2	5	3	3	4	5	5	5	4	3	3	3	5	3	4	4	5	4	3	3	2	3	3	3	1	3	3	3	3	3	3	3	3	3	4	4	7	4	4	4	1
1	3	2	1	4	5	2	1	3	3	3	4	3	3	4	2	4	3	4	3	3	3	3	3	2	4	4	2	3	3	3	1	3	3	3	3	3	3	3	4	5	3	5	4	3	6	4	3
2	2	2	1	2	2	3	1	3	3	4	3	5	5	5	5	5	5	5	5	5	4	3	3	3	2	2	2	3	3	3	1	3	3	3	3	3	2	4	3	3	3	4	5	4	5	5	2
2	1	2	1	1	1	4	2	3	5	3	4	2	3	5	4	4	4	3	3	4	4	3	4	4	2	4	2	3	2	4	1	4	2	2	4	4	2	2	2	4	4	5	4	6	6	5	3
2	2	2	1	3	1	4	1	4	4	3	4	5	5	5	3	5	4	4	4	4	4	3	3	3	3	2	2	3	2	4	1	3	3	3	4	4	3	4	3	4	3	4	6	5	5	5	1
2	1	2	1	3	1	1	1	4	4	4	4	3	4	4	3	3	4	4	4	5	4	5	5	5	5	5	2	3	2	4	1	3	3	3	3	3	4	2	3	3	3	4	4	5	4	4	4
1	2	2	1	3	1	1	1	5	5	5	5	5	5	5	5	5	5	4	5	5	5	5	5	5	5	5	2	3	2	2	1	4	2	3	4	4	5	2	2	2	2	6	6	6	6	7	4
2	5	2	2	1	1	3	2	4	2	3	3	4	4	5	2	4	4	3	4	5	4	4	4	3	1	2	2	3	1	2	1	4	4	4	2	4	2	1	2	5	5	6	5	5	4	5	3
2	2	2	1	3	1	1	1	5	4	5	5	3	4	5	4	5	5	5	5	3	5	5	5	5	5	5	2	4	4	5	2	2	4	5	5	3	4	2	5	2	2	5	5	7	6	6	4
1	2	2	1	3	2	3	1	5	4	4	5	3	4	4	4	4	4	4	4	4	4	3	3	3	3	3	2	4	4	4	2	4	4	4	3	4	4	2	2	3	4	5	5	5	4	4	3
2	2	2	1	1	1	3	1	3	4	5	4	3	2	2	4	3	5	4	3	2	4	4	4	3	5	3	2	5	5	5	2	5	3	2	4	4	3	3	4	3	5	4	3	5	7	2	3
1	3	4	1	1	1	1	1	4	5	4	3	4	3	4	2	4	2	3	4	2	3	5	2	3	5	5	2	5	2	5	2	5	2	5	4	5	4	4	2	5	5	5	6	6	5	5	1
1	1	2	1	3	1	4	1	5	5	5	3	5	3	3	3	4	3	5	5	3	3	4	4	4	3	3	2	2	4	4	2	5	5	4	3	3	4	3	3	3	3	6	6	5	5	6	2
1	4	2	1	5	4	3	1	4	4	4	3	4	5	4	3	3	4	4	4	4	4	4	4	4	3	4	2	2	4	4	1	4	4	4	3	3	4	4	4	4	4	5	5	4	4	5	4
2	3	2	2	2	2	3	1	5	3	2	4	5	5	4	3	3	3	3	4	3	3	5	4	4	5	3	2	2	4	4	1	4	5	5	5	5	3	4	3	3	4	6	6	5	6	6	4
2	2	2	1	2	1	3	1	4	4	4	5	4	5	5	3	2	4	4	5	5	4	5	5	5	5	4	2	2	4	3	1	3	3	5	4	4	2	2	2	2	5	6	5	5	6	6	3
1	3	2	1	3	2	3	1	4	3	4	4	4	4	3	3	3	4	3	4	2	4	4	4	4	4	4	2	2	4	2	1	4	4	4	4	4	4	3	4	3	3	6	5	5	6	6	2
2	2	2	1	3	1	3	1	4	4	5	3	5	4	4	4	3	4	4	4	4	3	4	4	4	3	4	2	2	3	4	1	4	4	4	4	4	4	3	3	4	4	5	6	5	6	6	3
1	1	2	1	1	1	4	2	5	5	3	4	4	5	4	4	3	4	5	3	3	4	5	5	5	3	5	2	2	3	3	1	3	3	4	4	4	2	3	2	2	3	3	6	2	6	6	1
1	2	4	1	3	1	4	1	4	5	5	4	4	4	4	4	4	5	5	4	4	5	4	4	4	3	4	2	2	3	3	1	4	3	3	4	4	3	4	4	5	4	6	6	5	6	5	5
2	2	2	1	3	2	3	1	4	5	5	4	4	5	5	5	3	3	3	3	3	3	5	5	5	5	5	2	2	3	3	1	4	4	4	4	4	4	5	5	5	5	7	5	7	7	7	4
2	7	2	2	3	4	3	1	4	3	3	4	4	5	3	4	3	4	4	4	4	4	3	4	3	3	4	2	2	3	3	1	4	4	4	4	4	4	3	3	4	4	5	5	5	5	5	5
1	2	2	1	3	2	3	1	5	5	5	4	5	5	4	4	4	4	4	4	3	3	4	4	4	4	4	2	2	3	2	1	5	5	5	3	3	2	2	5	2	4	7	5	5	7	7	4
2	7	4	2	3	4	3	1	5	5	5	3	5	3	4	4	4	5	5	5	5	4	5	5	5	5	5	2	2	3	2	2	5	4	5	4	4	4	4	4	4	4	6	7	5	6	6	6
1	3	4	2	1	4	3	1	5	5	4	2	3	4	5	5	3	5	5	5	5	4	5	5	5	4	5	2	2	3	2	2	4	5	5	5	5	4	4	2	3	4	7	7	7	7	6	1
1	2	2	1	3	2	3	1	4	4	4	3	2	3	3	4	4	4	3	4	3	3	4	4	4	4	4	2	2	3	2	2	4	3	3	3	3	4	4	3	3	4	3	4	3	3	3	1
2	10	2	2	1	1	5	1	5	5	4	5	5	5	3	3	3	5	5	5	5	5	5	5	5	5	5	2	2	2	4	2	5	5	5	3	3	3	5	3	5	5	7	7	5	7	7	4
2	3	2	1	4	2	3	1	4	4	5	4	5	5	4	3	4	5	5	4	4	4	4	4	5	4	4	2	2	2	4	2	4	4	4	3	3	3	2	2	3	3	6	5	5	5	5	4
2	1	4	1	1	2	4	1	4	4	3	4	4	5	4	2	1	4	4	4	2	3	4	4	4	2	3	2	2	2	4	1	4	4	4	4	3	4	2	3	3	3	5	5	5	5	6	1

2	4	2	1	3	3	3	1	4	3	3	3	5	5	4	4	4	4	4	4	3	3	4	4	4	3	3	2	2	2	4	1	4	4	4	4	4	4	4	4	4	5	6	6	6	7	7	6
1	7	4	2	2	3	3	2	5	5	5	5	5	5	5	5	5	5	5	5	4	4	4	4	4	3	3	2	2	2	3	1	3	4	3	4	3	3	3	3	4	3	5	5	6	5	4	4
2	4	2	1	1	5	3	1	5	4	4	4	4	5	4	3	3	3	4	3	3	3	4	4	4	4	4	2	2	2	2	1	4	4	4	4	4	4	4	4	4	4	5	5	5	5	5	5
1	2	2	1	1	1	2	1	4	4	4	4	4	4	4	4	4	4	4	4	4	4	4	4	4	4	5	2	2	2	2	1	4	4	4	4	4	4	2	4	3	4	6	6	6	5	5	4
1	1	2	1	3	1	1	1	4	4	4	3	5	5	4	4	4	4	4	5	4	4	4	4	4	4	4	2	2	2	2	1	4	4	4	4	4	2	2	2	2	2	6	6	6	6	6	3
1	2	2	1	2	1	1	1	3	4	3	4	5	5	4	3	3	4	3	4	3	3	4	4	4	3	2	2	2	1	2	1	4	4	4	3	3	2	2	2	2	2	4	6	4	4	5	3
2	3	4	2	2	3	3	1	4	4	5	3	5	5	4	4	3	4	4	4	4	4	4	5	4	4	5	2	1	1	1	1	4	4	4	4	4	3	3	3	3	2	5	6	4	5	5	1
2	1	2	1	3	1	1	1	4	3	4	4	2	3	4	4	2	2	2	3	2	2	3	4	4	3	3	2	1	1	1	1	4	4	3	3	3	4	4	3	4	4	5	5	4	5	5	6
1	1	2	1	3	1	1	1	3	3	3	3	4	3	4	3	4	3	4	3	3	4	3	4	3	4	4	2	1	1	1	1	3	3	4	4	4	3	3	3	3	3	5	6	3	5	6	6
1	7	2	2	2	2	3	1	5	3	3	1	4	3	2	2	1	3	4	5	3	5	2	4	5	3	5	2	1	1	1	2	2	4	2	5	4	3	2	3	3	2	6	7	2	4	2	1
2	5	2	1	3	5	3	1	5	3	5	3	4	3	3	3	3	3	4	4	3	3	5	5	5	4	4	2	1	1	1	2	3	3	3	3	3	4	4	4	4	4	6	6	6	6	6	5
2	1	4	1	1	1	1	1	5	5	5	5	5	5	5	4	4	4	4	4	2	3	3	3	3	2	2	2	1	1	1	2	2	2	2	2	2	3	3	3	2	2	2	2	3	3	3	1
2	3	2	1	3	2	3	1	4	3	4	2	5	4	4	3	4	4	4	4	4	4	4	4	4	4	4	2	1	1	1	2	4	4	4	4	4	4	4	4	4	4	6	5	5	5	5	4
2	2	2	1	3	1	1	1	2	2	2	2	3	4	4	3	3	3	3	3	4	2	4	2	3	3	3	2	1	1	1	2	4	3	4	3	3	3	3	3	3	3	4	4	4	3	3	1
2	7	2	2	2	3	3	2	5	4	5	3	4	5	4	5	3	4	2	3	3	4	5	4	2	3	4	2	1	1	1	1	5	3	4	2	4	3	4	3	5	2	4	6	5	3	4	2
1	2	2	1	3	2	2	1	4	5	4	2	5	5	2	1	2	5	5	4	5	5	4	4	4	5	5	2	1	2	1	1	1	5	5	4	4	4	5	5	5	5	6	6	7	6	7	4
2	1	2	1	3	1	1	1	5	5	5	5	2	3	4	2	1	4	4	4	3	4	4	4	4	4	4	2	2	2	2	1	5	5	5	3	3	4	4	5	5	3	7	7	7	7	7	4
2	2	2	1	3	1	1	1	4	5	4	3	5	4	4	4	4	3	3	5	5	3	5	5	5	5	5	2	2	2	2	1	5	5	5	5	5	4	4	4	4	4	6	7	6	7	7	6
1	4	2	1	3	2	3	1	5	4	5	5	2	4	1	2	2	4	5	5	4	5	4	4	4	2	3	2	2	2	2	1	5	4	5	4	4	5	4	3	4	5	6	6	5	6	6	5
1	2	2	1	2	1	3	1	5	4	5	4	5	5	2	4	2	4	5	5	4	4	4	4	4	4	4	2	2	2	2	1	4	4	4	4	4	4	5	4	4	4	6	7	5	6	6	4
2	1	1	1	2	1	4	1	5	4	5	4	5	5	4	3	3	4	4	4	4	3	5	4	5	5	5	2	2	3	2	1	4	5	5	5	5	4	3	3	3	4	7	6	5	5	5	1
1	3	2	2	3	2	3	1	5	4	5	4	5	5	5	5	5	3	3	4	3	4	5	5	5	5	5	2	2	3	4	1	5	4	5	5	5	4	4	4	4	4	6	6	6	6	7	6
2	1	2	1	3	1	1	1	5	4	5	5	5	5	4	5	4	4	4	5	5	4	4	4	4	4	5	2	2	3	2	1	5	5	4	4	4	5	4	5	4	5	6	6	5	6	6	1
2	2	2	1	2	1	3	1	5	5	5	5	5	5	5	2	4	5	4	4	4	5	5	5	5	5	5	2	2	2	5	1	5	5	5	5	5	2	1	1	1	2	7	5	5	7	7	4
2	3	2	2	1	2	3	1	3	3	4	5	5	5	5	2	2	3	2	2	3	3	5	4	5	5	1	2	2	2	5	2	5	5	5	5	5	4	4	5	5	5	7	6	1	6	7	6
1	8	2	2	1	1	2	2	5	5	5	3	1	1	1	1	1	5	5	5	5	5	5	5	5	5	5	2	2	2	4	2	5	5	5	5	5	4	1	5	5	4	7	7	7	7	7	1
1	7	2	2	2	3	3	1	5	5	5	5	4	5	5	3	3	4	4	4	4	4	5	5	5	5	5	2	2	2	4	2	5	5	5	5	5	5	5	5	5	5	7	7	7	7	7	4
1	2	2	1	3	1	2	1	4	4	4	5	3	4	5	5	5	3	4	5	4	5	5	5	5	5	5	2	2	2	4	2	5	5	5	5	5	5	5	5	5	5	7	7	7	7	7	6
1	3	2	1	3	2	2	1	5	5	5	5	5	5	5	5	5	5	5	5	5	5	5	5	5	5	5	2	2	1	5	2	5	5	5	5	5	5	5	5	5	5	7	7	7	7	7	6
2	2	2	1	3	2	3	1	4	3	3	3	5	5	2	2	2	2	1	4	3	4	5	5	5	5	5	2	2	1	4	5	5	4	5	5	5	4	5	5	5	3	5	5	6	6	6	4
1	6	2	2	2	3	3	1	4	4	4	4	4	5	4	4	4	3	3	3	3	3	5	5	5	5	5	2	2	1	2	5	5	5	5	5	5	5	5	4	5	5	7	7	7	7	7	4
2	4	2	2	3	2	4	2	4	3	2	4	5	4	3	3	2	5	5	5	3	3	4	5	5	3	4	2	3	4	2	5	4	2	1	5	5	3	3	2	2	4	5	3	3	6	5	3
1	1	2	1	2	1	3	1	5	5	5	5	5	5	5	5	5	5	5	5	5	5	5	5	5	2	2	2	3	3	4	5	4	5	5	5	5	5	5	5	5	5	7	7	7	7	7	1
2	1	2	1	3	1	3	1	1	1	2	2	4	4	4	3	4	4	5	5	3	5	5	5	5	5	5	2	3	3	3	5	4	5	5	5	5	5	5	5	5	4	7	7	7	7	7	4
2	10	2	2	2	2	5	1	5	5	5	5	5	5	5	5	5	5	4	4	4	4	5	5	5	5	5	2	3	2	3	5	5	5	5	5	5	5	5	5	5	5	7	7	7	7	7	6
1	2	2	1	2	1	3	1	4	4	3	3	3	3	4	2	3	5	5	4	3	4	1	1	2	3	2	2	4	4	3	5	4	3	5	2	4	3	3	5	3	4	4	6	5	6	5	3
2	4	4	2	4	2	4	1	4	4	3	4	4	4	5	2	3	3	5	3	3	4	3	3	4	1	2	3	3	2	4	1	1	1	1	3	3	1	1	1	1	2	3	5	2	3	2	1
1	1	2	1	1	1	4	1	4	3	4	3	3	4	4	3	3	2	2	4	3	4	4	4	3	4	4	3	2	4	2	2	3	2	4	3	3	2	2	3	2	3	6	6	4	6	5	3
2	2	2	1	3	1	4	1	3	3	3	2	3	3	4	3	3	4	4	5	3	4	4	4	4	4	3	3	2	3	3	2	3	2	3	3	3	3	3	3	4	3	4	4	3	3	3	3
2	2	2	1	3	1	1	1	4	4	5	3	2	4	4	3	3	4	4	5	4	2	5	4	4	3	3	3	2	2	4	2	2	2	2	1	1	3	1	1	1	4	6	6	5	6	6	1
2	2	2	1	3	2	3	1	5	3	5	4	4	4	3	5	3	4	5	4	4	5	5	5	4	4	3	3	2	2	4	2	2	3	2	3	3	3	1	3	3	3	7	6	5	6	6	2
1	4	2	2	3	3	2	1	4	4	3	3	3	4	4	4	3	4	3	4	3	3	4	3	4	3	3	3	3	1	5	2	2	2	3	3	3	2	3	3	3	2	5	4	6	4	5	2
1	1	4	1	3	1	1	1	3	3	4	3	3	4	3	4	4	3	5	4	4	4	2	2	2	1	3	3	3	1	5	2	1	1	1	1	1	4	5	5	5	5	3	4	4	5	4	1
2	2	2	1	3	1	1	1	4	4	3	3	5	5	4	3	4	4	5	3	3	3	4	4	4	3	4	3	3	1	5	2	3	3	3	2	2	3	4	3	3	4	4	3	5	4	3	4
2	2	2	1	2	1	4	1	4	4	3	3	1	4	4	3	4	3	3	4	3	3	3	3	3	2	2	3	3	1	5	2	2	2	2	2	2	1	1	2	2	2	4	3	3	4	4	1
1	2	2	1	3	1	1	1	3	2	3	3	5	4	5	4	4	3	3	3	4	3	3	3	3	2	1	3	3	1	5	2	1	3	2	3	3	1	3	1	2	3	4	4	3	4	4	1
2	4	2	2	3	2	3	2	4	4	4	4	5	3	3	2	4	3	4	4	4	4	2	2	1	1	2	3	3	1	4	2	2	2	2	1	1	2	2	2	3	3	2	1	2	2	3	1
2	7	2	2	1	2	2	1	4	4	3	4	3	3	5	4	4	4	5	5	5	4	3	3	3	1	2	3	3	1	4	2	2	2	3	2	2	1	1	2	2	2	4	3	3	3	3	1
2	2	4	1	3	2	1	1	4	3	4	3	5	4	4	2	3	3	2	3	3	3	3	3	3	3	3	3	3	1	4	2	2	2	2	2	2	1	2	2	3	3	4	4	4	4	4	1
1	1	4	1	1	1	1	1	4	3	5	3	4	4	5	3	3	4	3	4	5	3	3	3	3	2	2	3	3	1	4	2	2	3	3	2	2	3	2	4	2	3	4	5	4	5	4	3
2	1	2	1	3	1	4	1	3	3	3	3	4	3	4	3	4	3	3	3	3	3	4	3	4	2	3	3	3	1	4	2	2	3	2	2	2	2	1	2	3	3	4	3	4	3	4	1
2	3	2	1	3	3	3	1	2	2	3	2	4	4	3	3	3	4	4	4	3	3	3	2	2	2	2	3	3	2	4	2	2	2	2	3	2	3	2	2	2	3	3	4	4	4	4	3
1	1	2	1	3	1	1	1	4	4	3	4	4	4	4	3	2	4	4	4	4	4	3	3	3	3	3	3	3	2	3	2	2	2	2	2	2	2	2	2	2	2	4	7	5	5	4	1
1	1	4	1	1	6	1	1	3	4	3	4	5	5	5	4	5	3	3	3	3	3	2	2	2	2	2	3	3	2	3	2	2	2	2	2	2	1	1	1	1	1	3	2	1	1	1	1
2	3	2	2	3	3	3	2	4	3	4	3	5	5	5	4	4	4	3	3	3	5	4	5	5	5	4	3	3	2	3	2	2	5	3	3	3	5	2	5	4	3	6	5	5	6	5	5
1	2	2	1	3	2	3	1	4	3	4	5	4	4	5	4	3	4	3	4	3	3	3	3	3	3	3	3	3	2	3	2	2	3	3	3	3	3	2	2	2	3	4	4	3	4	4	1
2	2	2	1	1	2	3	1	3	4	3	4	4	4	3	3	3	3	4	4	3	4	4	3	4	3	4	3	3	4	1	2	2	4	3	3	4	3	1	3	1	5	3	7	7	3	4	2
1	2	2	1	3	2	3	1	3	4	5	4	5	5	2	5	3	4	4	5	4	4	3	3	3	3	2	3	3	3	5	2	2	2	2	2	2	4	4	2	4	2	3	5	3	4	2	3
2	8	2	2	2	4	2	1	5	5	5	4	4	4	5	5	5	4	5	5	5	5	3	2	3	1	2	3	3	3	4	2	1	1	1	1	1	3	4	3	4	4	3	5	4	3	3	3
2	2	2	1	3	1	1	1	4	4	5	4	4	5	4	4	3	3	4	3	3	4	5	4	5	3	4	3	3	3	4	2	3	3	3	3	4	3	4	4	4	4	5	6	3	5	5	2
1	2	2	1	3	2	3	1	4	4	4	4	5	5	5	5	5	3	3	3	3	3	3	3	3	3	3	3	3	3	3	2	2	2	2	2	2	2	2	2	2	3	3	4	3	4	4	3
2	1	2	1	2	1	1	1	4	4	4	3	5	5	4	4	4	4	5	4	5	3	4	4	3	2	2	3	3	3	3	2	3	3	3	3	2	2	3	2	2	2	5	6	4	3	3	1
2	2	2	1	3	2	3	1	4	4	4	5	5	5	5	5	5	4	4	4	5	5	3	3	3	2	2	3	3	3	3	2	2	2	2	2	2	3	3	3	3	4	3	4	3	3	3	2
1	4	2	1	1	2	3	1	3	4	3	4	4	4	1	2	3	3	2	3	4	3	5	5	4	2	3	3	3	3	3	2	4	3	4	2	4	3	3	3	4	3	5	5	5	4	5	3
1	2	2	1	2	2	3	1	5	3	5	4	3	3	4	4	3	4	3	3	3	4	3	3	3	2	2	3	3	3	3	2	2	2	2	2	2	3	2	2	2	3	3	4	4	4	3	1
2	8	2	2	2	2	3	1	3	3	4	3	5	5	5	5	4	3	3	3	3	3	2	2	2	2	2	3	3	3	3	2	2	2	2	2	2	2	2	2	2	2	2	2	2	2	2	2
1	1	2	1	1	1	4	1	3	3	4	2	2	4	2	3	4	3	5	5	5	4	2	2	2	2	1	3	3	3	3	2	2	2	2	1	2	3	3	3	3	4	3	3	3	3	3	3
1	1	4	1	1	1	1	1	3	3	3	3	2	4	3	3	3	5	4	4	4	4	3	3	4	4	4	3	3	3	3	2	2	4	3	3	3	3	4	3	3	3	4	4	4	5	6	1
2	1	2	1	1	1	1	1	2	2	2	2	2	3	4	3	3	4	4	4	4	4	3	3	2	2	2	3	3	3	3	2	3	3	3	2	2	2	2	2	2	2	4	6	4	3	2	4
1	7	2	2	4	4	2	1	5	4	4	4	5	5	5	5	4	3	4	5	5	5	1	1	1	1	2	3	3	3	2	2	3	3	3	3	2	2	2	3	2	3	3	5	4	3	4	2
1	3	2	2	2	4	3	1	4	4	5	4	4	3	4	5	2	4	4	4	4	5	3	1	2	1	3	3	3	3	2	2	1	1	1	2	2	4	4	4	4	4	4	5	5	3	3	3
2	1	2	1	1	1	1	1	3	4	1	3	1	2	4	1	2	5	2	1	4	3	2	1	3	3	1	3	3	3	2	2	4	4	3	2	3	4	4	3	3	2	4	4	5	2	6	3
1	8	2	2	2	2	3	1	4	5	3	4	5	4	4	5	5	4	4	5	5	5	5	5	5	5	5	3	3	2	3	2	4	5	4	2	3	4	4	4	5	4	7	7	6	5	5	3
2	6	2	2	2	2	2	1	5	4	4	2	5	4	3	3	3	4	4	4	4	4	3	3	3	3	3	3	3	2	3	2	2	3	3	3	3	3	2	3	3	3	4	4	3	4	4	2
2	1	4	1	1	3	3	1	4	4	3	4	2	3	3	3	3	5	3	4	3	4	4	3	4	2	1	3	3	2	3	2	2	2	2	3	3	3	3	3	3	2	3	7	7	4	3	1
2	1	2	1	3	1	1	1	4	4	3	4	5	4	3	4	4	5	4	3	2	2	1	1	2	1	1	3	3	2	3	2	3	3	2	2	2	3	4	4	4	3	3	5	4	6	6	2
1	2	2	1	3	2	3	1	5	5	5	4	5	5	4	3	4	4	3	5	4	3	4	3	4	3	3	3	3	2	2	2	2	2	2	3	3	5	4	4	5	4	5	7	4	5	5	2
2	2	4	2	3	4	3	1	3	3	4	2	4	3	3	4	3	3	3	4	3	3	3	3	3	3	3	3	3	1	3	2	3	3	3	2	2	1	1	1	1	2	4	7	2	4	4	1
1	2	3	1	3	1	1	1	5	4	5	4	5	5	2	2	2	4	3	5	5	5	5	4	5	1	3	3	3	1	2	2	2	2	3	1	1	1	1	1	1	4	4	7	4	4	4	1
1	2	2	1	3	1	1	1	4	5	4	5	5	5	4	5	5	3	1	2	3	3	5	5	5	5	5	3	4	5	1	2	3	4	3	3	3	2	2	3	4	4	5	4	6	6	7	6
2	1	2	1	3	1	2	1	4	5	4	3	4	5	4	2	2	5	4	3	3	4	2	2	2	2	2	3	4	4	4	2	2	2	2	2	2	2	2	2	2	3	2	5	2	2	2	1
1	1	1	1	1	1	3	1	4	4	4	3	5	5	5	4	4	4	4	5	3	4	4	4	4	4	5	3	4	4	4	2	4	3	3	4	3	2	2	3	3	4	5	5	4	5	4	1
1	2	2	1	2	1	1	2	3	4	3	2	3	4	4	2	3	4	3	4	3	3	2	3	3	2	3	3	4	3	4	2	3	4	3	3	4	3	3	4	3	4	4	4	3	4	5	3
1	2	2	1	2	2	3	1	3	2	4	3	4	3	3	3	4	3	3	3	3	4	4	3	3	4	3	3	4	3	4	2	3	3	3	2	2	2	2	2	3	3	3	5	4	3	4	3
2	4	2	1	1	2	2	1	5	4	5	3	4	4	4	5	3	4	4	3	3	4	5	4	5	4	3	3	4	3	3	2	2	2	2	2	2	4	3	3	4	4	7	7	6	7	7	4
1	2	2	1	3	2	3	1	2	1	1	2	5	4	4	4	5	5	4	4	4	5	1	1	1	1	1	3	5	5	5	2	1	2	4	4	4	4	4	4	4	5	5	2	2	2	5	1
1	2	2	1	3	1	1	1	4	3	5	4	2	4	3	2	3	4	4	4	2	4	5	5	5	4	4	3	2	4	4	3	4	4	4	3	3	4	4	4	3	4	5	7	6	6	6	4
1	1	2	1	1	2	3	1	3	3	3	3	3	3	3	3	3	3	3	3	3	3	3	3	3	3	3	3	2	4	3	3	3	3	4	4	4	3	4	3	4	3	4	3	5	5	4	3
2	6	3	2	2	2	3	1	3	3	5	3	3	3	3	3	2	4	3	3	4	3	4	3	3	3	3	3	2	4	3	3	2	3	3	3	3	2	3	3	3	3	3	5	3	4	3	2
1	1	1	1	3	1	1	1	4	3	4	3	4	4	4	5	4	4	4	4	4	4	4	4	5	3	4	3	2	2	3	3	3	4	4	4	4	3	5	4	5	4	5	6	6	6	7	4
1	6	4	2	4	2	3	1	4	4	4	4	4	5	4	5	4	4	4	4	2	2	4	4	4	2	4	3	2	2	2	3	2	3	3	4	4	4	4	4	4	5	6	6	6	7	6	1
2	7	1	2	4	6	3	1	5	5	5	1	4	4	5	3	4	3	3	3	4	4	5	3	4	3	4	3	3	5	5	3	2	3	4	1	1	1	1	3	2	3	5	7	4	5	5	4
2	6	2	2	1	1	4	2	5	4	5	3	3	4	5	5	4	3	5	5	3	4	4	4	5	5	3	3	3	5	5	3	4	5	5	3	5	4	5	5	3	3	7	7	3	7	5	3
1	2	2	1	1	2	4	1	5	4	4	4	3	3	4	4	3	5	5	5	5	4	5	5	5	3	3	3	3	5	5	3	3	3	3	3	3	4	4	5	5	5	7	7	5	7	7	3
2	3	2	1	2	2	3	1	5	4	4	4	5	5	4	5	3	5	4	5	3	3	3	3	3	3	3	3	3	5	5	3	3	3	3	3	3	2	2	2	2	5	4	3	3	3	3	3
1	2	2	1	3	2	3	1	4	4	4	3	5	5	3	2	2	4	4	3	3	5	4	4	4	3	3	3	3	5	5	3	2	3	3	3	3	4	4	4	4	2	6	4	4	6	6	4
1	2	2	1	3	1	1	1	3	4	3	4	4	4	5	3	2	4	3	4	4	3	5	5	5	5	5	3	3	5	5	3	3	3	3	3	3	2	2	2	2	3	6	4	6	6	6	1
1	2	1	1	1	1	1	1	3	3	3	3	4	4	5	4	5	3	4	3	3	3	3	3	3	3	3	3	3	5	5	3	3	3	3	3	3	3	3	3	3	3	3	7	3	3	3	1
1	7	2	2	3	2	3	2	5	5	5	4	5	5	3	2	1	3	4	3	4	3	5	5	5	4	4	3	3	5	4	3	4	4	5	4	4	2	2	2	2	4	5	6	6	6	6	4
1	3	2	1	3	2	3	2	4	4	5	3	3	5	4	2	2	3	4	3	3	4	3	3	3	2	1	3	3	5	4	3	3	3	2	3	3	2	1	2	2	3	3	2	1	3	2	3
1	2	2	1	3	2	4	1	3	3	3	3	3	3	4	4	4	5	5	5	5	5	3	3	3	3	3	3	3	5	4	3	3	3	3	3	3	3	4	3	4	3	4	4	4	4	4	4
2	7	1	4	4	6	3	1	5	4	4	3	3	5	4	4	4	5	5	5	4	4	4	4	4	5	5	3	3	5	3	3	3	3	3	3	3	5	5	5	3	5	5	5	5	5	5	4
2	1	2	1	3	1	1	1	4	5	4	3	5	4	4	4	3	4	4	4	3	3	4	4	4	1	1	3	3	4	5	3	2	2	4	2	2	2	1	2	2	1	5	7	4	5	5	1
2	2	2	1	2	1	3	1	3	5	1	4	3	5	5	1	1	3	3	1	3	3	5	5	5	3	3	3	3	4	5	3	3	3	3	1	1	1	1	1	1	2	2	2	2	3	4	3
2	1	2	1	3	1	3	1	4	4	3	4	5	4	5	5	5	4	4	4	4	4	3	3	4	3	3	3	3	4	5	3	2	2	2	2	2	2	2	2	2	3	4	4	4	4	4	2
1	2	1	1	3	2	3	1	3	2	5	3	5	5	3	4	5	2	5	5	3	3	5	5	5	3	4	3	3	4	5	3	2	1	2	3	3	2	2	1	3	3	6	7	6	7	6	1
2	6	2	2	3	4	3	1	5	5	5	5	4	5	4	4	4	3	5	4	4	4	3	2	2	1	1	3	3	4	4	3	3	3	3	3	2	3	3	3	4	4	4	3	4	4	4	2
1	3	2	1	3	2	3	1	4	5	3	4	4	4	4	4	4	3	3	5	3	3	4	4	4	2	2	3	3	4	4	3	2	3	3	3	3	2	4	3	3	3	5	5	7	5	4	4
1	3	4	1	3	1	1	1	5	4	3	3	4	4	4	4	4	4	4	4	3	3	4	4	4	2	3	3	3	4	4	3	3	3	3	3	3	3	3	3	3	3	5	5	4	6	6	2
2	8	2	1	1	1	3	2	4	4	3	2	5	5	5	5	4	4	5	4	4	4	3	3	3	3	3	3	3	4	4	3	3	3	2	2	2	2	3	3	3	3	2	1	1	1	1	2
2	2	2	1	3	1	1	2	4	4	4	3	4	4	4	3	3	4	4	4	3	3	4	4	4	3	3	3	3	4	4	3	4	4	4	3	3	3	3	3	3	3	5	6	4	5	5	3
1	1	2	1	2	1	1	2	4	4	3	4	4	3	4	3	4	5	4	4	4	5	3	3	3	3	3	3	3	4	4	3	3	3	3	3	3	4	4	3	4	3	6	5	5	4	6	3
2	3	1	1	3	2	3	1	4	4	4	4	5	5	3	3	3	5	5	5	5	5	4	4	5	4	5	3	3	4	4	3	5	5	5	3	3	4	5	2	4	4	6	6	6	7	7	4
1	2	2	1	3	1	3	1	4	4	4	4	5	5	5	5	5	5	5	5	4	5	4	4	4	3	3	3	3	4	4	3	3	3	3	3	3	4	3	3	4	3	4	5	5	4	4	2
1	1	2	1	2	1	4	1	4	4	3	4	4	4	4	3	4	4	4	4	4	4	4	4	4	4	4	3	3	4	4	3	2	3	5	5	5	3	2	3	5	4	6	6	7	6	6	6
2	5	2	2	5	2	2	1	4	4	4	3	4	4	4	2	2	4	4	4	4	4	4	4	4	4	4	3	3	4	4	3	3	3	3	3	3	4	4	4	4	4	5	5	5	5	5	5
2	2	2	1	3	1	2	1	3	4	4	4	5	5	3	3	3	5	5	5	5	5	5	5	5	5	5	3	3	4	4	3	5	4	3	4	4	3	3	4	5	4	7	3	6	6	6	4
2	2	2	1	3	1	4	1	3	4	3	3	3	4	4	4	3	3	3	3	3	3	3	3	3	4	4	3	3	4	4	3	3	3	3	3	3	3	3	3	3	4	5	4	4	5	5	2
1	2	4	1	2	1	1	1	3	4	5	4	5	5	5	3	3	4	4	3	3	3	4	4	4	4	4	3	3	4	4	3	3	3	4	4	4	3	2	1	1	3	6	6	6	6	6	1

1	3	2	1	2	2	3	1	5	3	5	5	5	5	4	4	3	3	3	3	3	4	4	4	4	4	4	3	3	4	4	3	3	4	4	3	3	4	5	5	5	4	7	4	5	5	5	3
1	4	2	1	1	2	3	1	5	3	3	5	3	3	5	2	2	4	4	4	3	3	4	4	4	1	2	3	3	4	4	3	1	2	4	2	2	4	4	2	4	3	4	4	2	3	3	4
2	7	2	1	2	2	3	2	4	3	4	3	5	5	4	3	3	4	4	4	4	4	3	3	3	3	3	3	3	4	4	3	3	3	3	3	3	2	2	2	2	3	3	3	2	3	3	2
1	2	4	1	2	2	1	1	4	3	5	3	5	4	4	4	4	5	4	5	5	4	5	5	5	2	4	3	3	4	4	3	3	3	3	3	3	4	2	2	2	4	6	7	4	5	5	1
2	3	2	1	3	2	3	1	4	3	3	4	2	3	4	2	3	3	4	3	3	3	2	2	2	1	2	3	3	4	4	3	2	1	1	1	1	3	3	3	5	5	3	4	4	4	4	2
2	2	4	2	3	3	3	1	4	3	5	4	2	5	3	3	2	4	4	5	2	4	4	3	4	3	4	3	3	4	4	3	3	4	4	4	4	3	2	4	2	2	5	6	5	6	6	4
2	3	2	1	3	2	2	1	4	4	4	4	5	5	5	3	3	4	4	5	4	5	4	2	2	2	3	3	3	4	3	3	2	3	2	2	3	3	3	3	4	3	5	5	4	5	5	3
1	1	2	1	3	1	1	1	4	4	4	3	3	4	3	3	2	4	4	4	4	4	4	3	3	4	4	3	3	4	3	3	3	3	3	3	3	2	2	2	2	3	5	5	4	5	5	3
2	2	2	1	3	1	4	1	4	4	3	4	4	4	5	4	5	4	5	4	4	5	5	5	5	4	4	3	3	4	3	3	3	4	4	3	3	2	3	3	4	3	5	5	6	6	5	3
1	2	1	1	3	2	2	1	4	3	4	3	5	4	4	2	4	3	4	4	4	4	3	3	4	2	2	3	3	4	3	3	3	4	4	3	3	2	3	3	2	4	6	4	4	5	4	1
1	2	2	1	1	1	1	2	3	3	4	3	2	2	3	3	3	3	3	3	3	2	2	2	3	3	3	3	3	4	3	3	3	3	3	3	3	3	3	3	3	3	5	4	5	5	5	1
2	1	2	1	3	1	4	1	3	3	3	3	3	4	4	2	2	4	4	5	4	4	3	3	3	3	3	3	3	4	3	3	2	3	2	2	2	3	3	3	3	3	3	3	3	4	3	1
2	1	4	1	1	1	3	1	3	3	3	3	3	2	3	4	3	4	3	3	3	3	3	4	4	3	3	3	3	4	3	3	3	5	1	4	4	4	5	5	5	5	7	3	5	7	6	6
2	1	1	1	1	1	1	1	3	3	3	4	3	4	3	3	3	3	3	4	3	4	4	3	2	2	3	3	3	4	3	3	3	3	3	3	3	3	3	3	3	3	4	4	4	4	4	1
1	1	2	1	5	1	4	1	3	3	3	3	2	3	3	3	3	2	2	2	2	2	4	4	4	2	3	3	3	4	3	3	3	3	3	3	3	2	3	2	3	3	4	4	4	4	4	4
1	2	2	1	1	1	4	2	4	4	3	4	4	4	5	4	4	4	5	3	3	3	4	4	3	4	3	3	3	4	2	3	4	3	4	3	4	4	4	3	3	3	6	6	6	5	5	4
1	2	2	1	1	1	4	2	4	4	4	4	4	4	4	4	4	5	5	5	5	5	3	3	3	3	3	3	3	3	4	3	1	1	1	1	1	3	2	2	2	2	4	5	4	4	4	1
1	3	1	1	1	2	3	1	4	4	5	3	3	3	4	3	4	3	3	5	3	2	3	3	3	3	3	3	3	3	4	3	3	2	3	3	3	3	4	3	2	5	4	4	7	3	4	1
1	5	4	2	3	4	3	1	5	4	5	5	4	5	4	4	2	5	5	5	5	5	3	3	3	1	3	3	3	3	5	3	2	3	3	4	4	5	5	5	5	4	4	6	4	4	4	3
2	2	2	1	3	1	1	1	5	5	5	4	4	5	5	5	4	5	5	5	5	5	3	3	4	3	3	3	3	3	4	3	4	3	3	4	4	3	3	4	3	3	4	4	5	4	4	2
1	2	4	1	3	1	1	1	5	4	4	4	5	4	4	3	4	4	4	4	4	5	3	3	3	2	3	3	3	3	4	3	5	3	3	3	3	2	2	2	2	2	4	7	4	5	5	1
2	1	2	1	2	1	4	1	4	4	4	4	5	5	5	4	4	5	5	5	5	5	4	4	4	4	3	3	3	3	4	3	4	4	3	3	3	3	3	3	4	3	4	4	4	4	5	4
1	1	2	1	3	1	4	1	3	4	4	4	4	4	4	4	4	4	4	4	4	4	4	4	4	3	3	3	3	3	4	3	3	3	3	3	3	3	2	2	2	2	5	7	5	5	5	4
1	1	2	1	3	1	5	1	4	3	5	3	3	4	2	2	3	2	3	4	2	2	5	5	4	4	4	3	3	3	4	3	3	3	3	3	3	4	2	3	2	4	6	5	6	5	6	1
2	8	2	2	1	2	2	2	3	3	4	4	4	3	4	5	4	4	4	4	4	4	4	3	3	3	3	3	3	3	4	3	4	3	4	3	4	3	4	3	4	3	5	4	5	4	5	3
2	1	2	1	2	2	3	1	3	3	3	3	3	4	4	3	3	5	4	5	3	5	4	4	4	4	4	3	3	3	4	3	4	3	4	3	3	3	2	2	2	3	5	7	4	6	6	2
1	4	2	1	4	1	1	1	3	3	3	3	3	3	3	3	3	3	3	3	3	3	3	3	3	3	3	3	3	3	4	3	3	4	3	3	3	3	3	3	3	3	5	4	4	5	4	3
2	5	2	2	1	2	3	1	3	3	5	5	5	5	4	3	3	5	5	4	3	4	3	4	3	3	3	3	3	3	4	3	3	3	3	3	3	3	3	3	4	4	3	3	3	3	3	3
1	6	2	2	1	2	3	1	3	3	5	5	5	5	4	3	3	5	5	4	3	4	3	3	3	3	3	3	3	3	4	3	3	3	3	3	3	3	3	3	3	4	4	3	3	3	3	3
2	7	2	2	1	1	4	1	3	3	3	3	3	3	3	3	3	4	4	4	3	4	3	3	3	3	3	3	3	3	4	3	3	3	3	3	3	3	2	3	2	3	4	4	4	4	4	1
1	2	1	1	3	2	2	1	3	2	4	5	5	5	5	2	3	5	5	5	5	5	5	5	5	3	3	3	3	3	4	3	3	3	3	4	4	2	1	2	1	4	3	7	3	3	2	1
2	8	2	1	2	2	3	2	5	5	5	3	3	3	3	3	3	3	3	3	3	3	3	3	3	3	3	3	3	3	3	3	3	3	3	3	3	3	3	3	3	3	4	4	4	4	4	4
2	2	2	1	3	1	1	1	5	5	5	5	5	5	5	5	5	5	5	3	3	5	1	3	3	3	3	3	3	3	3	3	3	3	3	3	3	3	3	3	3	3	4	4	4	4	4	3
1	3	2	2	1	1	3	1	5	5	5	3	5	5	5	3	3	5	4	4	4	4	3	3	3	3	3	3	3	3	3	3	3	3	3	3	4	4	4	4	4	4	4	4	4	4	4	3
1	2	2	1	3	2	2	1	5	4	3	4	5	5	3	3	3	5	4	5	3	4	5	5	5	5	5	3	3	3	3	3	3	3	3	5	5	3	2	2	2	4	7	7	4	7	7	4
2	1	4	1	1	1	1	1	5	4	5	3	3	4	4	2	3	3	3	3	3	3	5	5	5	5	5	3	3	3	3	3	3	3	4	3	3	1	1	1	1	1	7	5	5	7	7	1
2	2	2	1	2	2	3	1	4	4	4	5	3	3	3	4	3	3	3	3	3	3	3	3	3	3	3	3	3	3	3	3	3	3	3	3	3	3	3	3	3	3	4	3	3	3	3	4
2	2	2	1	3	1	4	1	4	4	3	4	3	4	4	2	3	3	3	3	3	3	4	4	4	2	3	3	3	3	3	3	2	3	3	3	3	3	2	3	3	3	3	3	3	2	3	3
2	2	2	1	3	1	3	1	4	4	4	3	3	3	4	3	3	3	3	4	3	3	3	4	4	3	3	3	3	3	3	3	3	4	3	3	4	3	3	3	3	3	6	6	6	6	4	2
1	8	2	1	4	3	5	1	4	4	4	4	4	4	3	3	3	4	4	4	4	4	4	4	4	4	4	3	3	3	3	3	3	3	4	3	3	3	3	3	3	3	5	5	5	5	5	1
1	6	2	2	1	4	2	1	4	4	4	4	4	4	4	4	4	4	4	4	3	4	3	3	3	2	2	3	3	3	3	3	3	3	3	3	3	3	3	3	3	3	4	4	4	4	4	4
1	2	2	1	1	1	1	1	4	4	4	4	4	4	4	4	4	4	3	3	3	3	3	3	3	3	3	3	3	3	3	3	3	3	3	3	3	3	3	3	3	3	4	4	4	5	4	3
2	2	2	1	3	1	4	1	4	4	4	4	4	4	4	4	4	4	4	5	4	4	4	4	4	4	4	3	3	3	3	3	3	3	3	3	3	3	3	3	3	4	5	5	5	5	5	3
1	2	2	1	3	1	3	1	4	4	3	3	3	3	3	3	3	3	3	3	3	3	3	3	3	3	3	3	3	3	3	3	3	3	3	3	3	3	3	3	3	3	4	5	4	4	4	1
2	2	2	1	3	2	3	1	4	4	4	4	4	4	4	4	4	3	3	3	3	3	5	5	5	5	5	3	3	3	3	3	4	4	4	4	4	4	5	4	5	5	6	6	6	6	6	3
2	3	2	1	3	3	3	1	4	4	4	4	4	5	4	4	4	3	3	3	3	3	5	5	5	5	5	3	3	3	3	3	3	4	5	4	4	5	5	5	5	5	7	6	6	6	7	4
2	4	2	2	3	3	3	1	4	4	4	4	4	4	4	4	4	3	3	3	3	3	5	4	5	4	5	3	3	3	3	3	3	3	4	4	4	5	5	5	5	5	7	6	6	7	7	3
1	4	2	1	2	1	3	2	3	4	4	3	3	3	3	3	3	3	2	3	3	3	3	3	3	4	5	3	3	3	3	3	3	3	3	3	3	3	3	3	3	3	4	6	4	4	4	1
1	2	2	1	2	1	3	1	3	4	3	3	3	3	3	3	3	3	3	3	3	3	3	3	3	3	3	3	3	3	3	3	3	3	3	3	3	3	3	3	3	3	4	4	4	4	4	3
1	6	2	2	3	3	2	1	3	4	5	3	5	5	5	3	3	4	4	4	4	4	4	4	4	3	3	3	3	3	3	3	3	4	4	3	3	4	3	4	4	4	6	4	4	6	5	4
1	2	2	1	2	1	1	1	3	4	4	3	2	4	3	4	3	3	3	5	2	5	4	4	4	5	4	3	3	3	3	3	3	3	3	3	3	3	4	4	4	4	6	5	6	6	6	3
2	2	2	1	2	2	3	1	3	4	3	4	4	3	3	3	3	4	3	3	3	4	3	3	3	3	3	3	3	3	3	3	3	3	3	3	3	3	3	3	3	3	3	5	3	3	3	2
2	2	2	1	3	1	1	1	3	4	3	4	3	3	3	4	4	5	5	4	4	4	4	4	4	3	4	3	3	3	3	3	3	3	3	4	4	4	3	3	4	3	4	4	5	5	5	2
1	6	3	2	2	2	3	2	4	3	4	4	3	4	4	3	4	5	3	4	4	5	4	4	3	4	2	3	3	3	3	3	3	2	4	4	3	2	3	4	4	3	5	4	6	5	4	2
2	2	2	1	3	1	1	2	4	3	3	4	3	4	4	4	4	4	5	5	5	5	4	4	4	3	3	3	3	3	3	3	3	3	3	3	4	3	2	3	3	4	4	6	3	4	4	1
2	2	2	1	3	1	1	1	4	3	3	3	3	3	3	3	3	3	3	3	3	3	3	3	3	3	3	3	3	3	3	3	4	3	3	3	3	3	3	3	3	3	4	4	4	4	4	4
2	2	2	1	3	1	2	1	4	3	3	3	3	3	3	3	3	3	3	3	3	5	4	3	3	3	3	3	3	3	3	3	3	3	3	3	3	3	3	3	3	3	4	4	4	4	4	4
2	1	2	1	3	3	3	1	4	3	3	3	5	4	5	4	3	5	5	4	4	4	3	3	3	1	2	3	3	3	3	3	3	3	3	3	3	3	3	3	4	3	4	4	3	3	3	2
1	2	2	1	3	1	1	1	4	3	3	4	3	3	3	3	3	3	3	3	3	3	3	3	3	3	3	3	3	3	3	3	3	3	3	3	3	3	3	3	3	3	4	4	4	4	4	4
2	1	2	1	3	1	1	1	4	3	4	4	3	4	4	5	5	5	5	4	4	4	3	3	4	5	4	3	3	3	3	3	2	3	3	4	4	3	2	2	3	2	5	5	4	5	6	2
2	2	2	1	3	1	1	1	4	3	4	4	3	4	4	3	3	3	3	3	3	3	4	4	4	4	4	3	3	3	3	3	3	3	3	3	3	3	3	3	3	3	5	5	5	6	6	6
2	2	4	1	3	3	3	1	4	3	4	3	5	5	3	3	3	3	2	3	3	3	3	3	3	3	3	3	3	3	3	3	3	3	3	3	3	3	3	3	3	3	4	3	1	3	2	1
1	10	4	1	5	1	1	2	3	3	3	3	3	4	3	3	3	3	3	3	4	3	3	3	3	3	3	3	3	3	3	3	3	3	3	3	3	3	3	3	3	3	4	4	4	4	4	6
2	3	2	1	3	1	3	2	3	3	3	3	3	3	3	3	3	3	3	4	4	4	4	3	3	3	3	3	3	3	3	3	3	3	3	3	3	3	3	3	3	3	4	4	4	4	4	2
2	2	2	1	3	1	1	2	3	3	3	4	3	3	3	3	4	3	3	3	3	3	4	4	4	4	3	3	3	3	3	3	4	3	3	3	3	3	3	3	3	3	4	4	4	4	4	2
2	2	2	1	2	1	1	2	3	3	3	3	5	3	4	4	4	3	4	4	3	2	4	4	4	4	4	3	3	3	3	3	3	3	3	3	3	3	4	4	4	4	4	4	4	4	4	2
1	5	3	2	1	2	4	2	3	3	4	3	4	4	3	3	3	4	3	4	3	4	3	3	3	3	3	3	3	3	3	3	3	3	3	3	3	3	3	3	3	3	4	4	5	5	5	2
2	2	2	1	3	1	1	2	3	3	4	4	3	4	3	3	3	3	3	3	3	3	3	3	3	3	3	3	3	3	3	3	3	3	3	3	3	3	3	3	3	3	4	4	4	4	4	6
2	1	2	1	1	2	3	2	3	3	3	3	3	3	3	3	3	3	3	3	3	3	3	3	3	3	3	3	3	3	3	3	3	3	3	3	3	3	3	3	3	3	4	4	4	4	4	3
2	1	2	1	2	1	1	1	3	3	3	4	3	4	3	3	3	3	3	3	3	3	3	3	3	3	3	3	3	3	3	3	3	3	3	3	3	3	2	2	2	2	4	4	5	4	5	2
2	2	2	1	2	1	3	1	3	3	3	3	3	3	3	3	3	3	3	4	3	3	5	4	4	3	3	3	3	3	3	3	3	3	3	3	3	3	2	2	3	3	4	4	3	5	4	3
2	1	2	1	2	1	1	1	3	3	3	3	3	3	3	3	3	3	3	3	3	3	3	3	3	3	3	3	3	3	3	3	3	3	3	3	3	3	3	3	3	3	4	4	4	4	4	3
1	1	2	1	1	1	1	1	3	3	3	3	3	3	3	3	3	3	3	3	3	3	3	3	3	3	3	3	3	3	3	3	3	3	3	3	3	3	3	3	3	3	4	4	4	4	4	1
2	1	2	1	3	1	1	1	3	3	3	3	4	3	3	3	3	5	5	5	4	4	4	4	4	4	4	3	3	3	3	3	3	3	3	3	3	3	3	3	3	3	4	4	3	4	4	1
2	1	2	1	1	1	1	1	3	3	3	3	3	3	3	3	3	3	3	3	3	3	3	3	3	3	3	3	3	3	3	3	3	3	3	3	3	3	3	3	3	3	4	4	4	4	4	6
1	1	2	1	1	2	2	1	3	3	3	3	3	3	3	3	3	3	3	3	3	3	3	3	3	3	3	3	3	3	3	3	3	3	3	3	3	3	3	3	3	3	4	4	4	4	4	1
1	2	2	1	1	1	5	1	3	3	3	3	3	3	3	3	3	3	3	3	3	3	3	3	3	3	3	3	3	3	3	3	3	3	3	3	3	3	3	3	3	3	3	4	4	3	3	1
2	2	2	1	3	1	4	1	3	3	3	3	3	2	3	3	3	4	4	4	4	3	3	3	3	3	3	3	3	3	3	3	3	3	3	3	3	3	2	3	3	3	4	4	3	4	4	3
1	9	2	2	1	2	2	1	3	3	3	3	3	3	3	3	3	3	3	3	3	3	3	3	3	3	3	3	3	3	3	3	3	3	3	3	3	3	3	3	3	3	4	4	4	4	4	3
2	6	2	1	1	1	3	1	3	3	3	3	3	3	3	3	3	3	3	3	3	3	3	3	3	3	3	3	3	3	3	3	3	3	3	3	3	3	3	3	3	3	4	4	4	4	4	3
2	5	2	2	2	1	3	1	3	3	3	3	3	3	3	3	3	5	4	4	5	4	3	2	3	2	3	3	3	3	3	3	3	3	3	3	2	2	1	2	3	3	3	3	3	3	3	2
2	2	2	1	1	1	3	1	3	3	3	3	3	3	3	3	3	3	3	3	3	3	3	3	3	3	3	3	3	3	3	3	3	3	3	3	3	3	3	3	3	3	4	4	4	4	4	1
1	4	2	1	1	1	1	1	3	3	3	3	3	3	3	3	3	3	3	3	3	3	3	3	3	3	3	3	3	3	3	3	3	3	3	3	3	3	3	3	3	3	4	4	4	4	4	2
1	2	1	1	2	1	1	1	3	3	3	3	3	3	3	3	3	3	3	3	3	3	3	3	3	3	3	3	3	3	3	3	3	3	3	3	3	3	3	3	3	3	4	4	4	4	4	2
2	1	2	1	1	1	1	1	3	3	3	3	3	3	3	3	3	3	3	3	3	3	3	3	3	3	3	3	3	3	3	3	3	3	3	4	3	3	3	3	3	3	4	4	4	4	5	1
2	1	2	1	2	1	1	1	3	3	3	3	3	3	3	3	3	3	3	4	3	3	4	4	4	4	4	3	3	3	3	3	3	3	3	3	3	3	3	3	3	3	4	4	4	4	4	4
1	5	2	2	1	2	3	1	3	3	3	3	3	3	3	3	3	3	3	3	3	3	3	3	3	3	3	3	3	3	3	3	3	3	3	3	3	3	3	3	3	3	5	5	3	3	4	3
1	1	2	1	1	1	3	1	3	3	3	3	3	3	3	3	3	3	3	3	3	3	3	3	3	3	3	3	3	3	3	3	3	3	3	3	2	3	3	3	3	3	5	6	6	5	5	4
1	1	2	1	1	1	1	1	3	3	3	3	3	3	3	3	3	3	3	3	3	3	3	3	3	3	3	3	3	3	3	3	3	3	3	3	3	3	3	3	3	3	4	5	4	4	3	4
1	1	2	4	1	6	1	1	3	3	3	3	3	3	3	3	3	3	3	3	3	3	3	3	3	3	3	3	3	3	3	3	3	3	3	3	3	3	3	3	3	3	4	4	4	4	4	5
1	2	2	1	3	1	1	1	3	3	3	3	5	5	5	4	3	4	4	4	3	3	3	3	3	3	3	3	3	3	3	3	3	3	3	3	3	3	3	3	3	3	4	4	4	4	4	3
1	2	2	1	1	1	3	1	3	3	4	3	4	3	3	3	3	3	3	3	3	3	3	3	3	3	3	3	3	3	3	3	3	3	3	3	3	3	3	3	3	3	4	4	4	4	4	3
1	3	2	1	2	2	3	1	3	3	3	3	3	3	3	3	3	3	3	3	3	2	2	2	2	2	2	3	3	3	3	3	3	3	3	3	3	3	3	3	3	3	4	4	4	3	3	4
2	1	2	1	1	1	1	1	3	3	3	3	3	4	3	3	3	3	3	3	3	3	3	3	3	3	3	3	3	3	3	3	3	3	3	3	3	3	3	3	3	3	4	4	4	4	4	3
2	1	2	1	1	1	2	1	3	3	3	3	3	3	3	3	3	3	3	3	3	3	3	3	3	3	3	3	3	3	3	3	3	3	3	3	3	3	3	3	3	3	4	4	4	4	4	6
1	1	2	1	3	2	1	1	3	3	5	3	3	3	3	3	3	3	3	3	3	3	3	3	3	3	3	3	3	3	3	3	3	3	3	3	3	3	3	3	3	3	4	4	4	4	4	5
1	1	2	1	1	1	4	1	3	3	3	3	4	3	3	3	3	3	4	4	4	4	3	3	3	3	3	3	3	3	3	3	3	3	3	3	3	3	3	3	3	3	5	5	5	5	5	1
2	1	2	1	2	1	2	1	3	3	3	3	3	3	4	4	3	3	3	5	4	5	2	3	3	3	3	3	3	3	3	3	3	3	3	2	2	3	3	3	3	3	4	4	4	4	4	3
1	2	4	1	3	1	1	1	3	3	3	3	3	3	3	3	3	3	3	3	3	3	3	3	3	3	3	3	3	3	3	3	3	3	3	3	3	2	2	2	2	3	4	4	4	4	4	3
2	2	2	1	3	2	2	1	3	3	3	3	3	3	3	3	3	3	3	3	3	3	3	3	3	3	3	3	3	3	3	3	3	3	3	3	3	3	3	3	3	3	4	4	4	4	4	3
1	2	2	1	1	1	1	1	3	3	3	3	3	3	3	3	3	3	3	3	3	3	4	3	3	3	3	3	3	3	3	3	3	3	3	3	3	3	3	3	3	3	4	4	5	5	4	2
2	1	2	1	1	1	3	1	3	3	3	3	3	3	3	3	3	3	3	3	3	3	3	3	3	3	3	3	3	3	3	3	3	4	4	3	3	3	3	3	3	3	4	5	4	4	4	6
2	2	2	1	2	1	3	1	3	3	3	3	3	3	3	3	3	3	3	3	3	3	3	3	3	3	3	3	3	3	3	3	3	3	3	3	3	3	3	3	3	3	4	4	4	4	4	4
1	2	2	1	2	1	3	1	3	3	3	3	3	3	3	3	4	4	4	4	4	4	3	3	3	3	3	3	3	3	3	3	3	3	2	3	3	3	3	3	3	3	4	4	4	4	4	4
1	1	2	1	3	1	1	1	3	3	3	3	3	3	3	3	3	3	3	3	3	3	3	3	3	3	3	3	3	3	3	3	3	3	3	3	3	3	3	3	3	3	4	4	4	4	5	3
2	2	2	1	1	1	3	1	3	3	3	3	4	3	3	3	3	3	4	4	3	3	3	3	3	3	3	3	3	3	3	3	3	3	3	3	3	3	3	3	3	3	4	4	4	4	4	3
2	2	2	1	2	2	2	1	3	3	3	3	3	3	3	3	3	3	3	3	3	3	3	3	3	3	3	3	3	3	3	3	3	3	3	3	3	3	3	3	3	3	4	4	4	4	4	2
1	3	2	1	3	2	3	1	3	3	4	3	5	4	3	2	3	3	3	3	3	3	3	3	3	2	3	3	3	3	3	3	3	3	3	3	3	2	2	3	4	3	3	3	3	3	3	4
2	1	1	1	2	1	1	1	3	3	3	3	3	3	3	3	3	3	3	3	3	3	3	4	4	3	3	3	3	3	3	3	3	3	3	3	3	3	4	3	3	3	4	4	4	4	4	1
2	2	2	1	2	1	2	1	2	3	2	2	4	4	4	3	4	3	3	3	3	3	3	3	3	3	3	3	3	3	3	3	3	3	3	3	3	3	3	3	3	3	4	4	4	4	4	3
2	5	2	2	2	2	3	1	3	4	3	3	3	3	3	3	3	3	3	3	3	3	3	3	4	3	3	3	3	3	2	3	3	3	3	3	3	3	3	4	3	3	4	3	4	4	4	3
1	4	2	1	3	2	3	1	5	5	4	4	5	4	5	5	4	4	5	4	5	5	3	3	2	2	3	3	3	2	4	3	4	3	2	3	3	3	4	3	3	4	3	3	4	4	3	2
2	1	2	1	3	1	1	1	3	4	5	4	4	4	4	2	3	4	4	4	4	4	4	4	4	3	3	3	3	2	4	3	3	3	3	3	3	3	2	2	3	3	5	6	4	5	6	1
1	2	2	1	3	1	1	1	5	4	3	4	3	3	4	4	4	4	5	5	5	4	4	4	4	4	4	3	3	2	3	3	3	3	3	3	3	3	3	3	3	3	5	6	4	6	6	1
2	2	2	1	3	2	3	1	4	4	4	3	5	3	5	5	5	4	5	5	4	4	4	4	4	4	4	3	3	2	3	3	4	4	4	4	4	3	2	2	2	4	5	5	4	5	5	1
1	3	2	1	3	3	3	1	4	4	3	3	3	4	4	4	3	4	4	4	4	4	3	3	3	3	3	3	3	2	3	3	3	3	3	3	3	4	4	4	4	4	5	6	6	5	6	2
1	1	2	1	2	1	4	1	3	3	5	3	4	4	4	3	3	4	5	5	3	4	5	5	5	5	5	3	3	2	3	3	3	3	4	3	3	4	3	3	3	4	5	5	5	5	5	4
2	2	2	1	3	1	3	1	3	3	3	4	3	2	4	4	4	3	3	3	2	3	3	3	3	3	3	3	3	2	3	3	3	3	3	3	3	3	2	3	3	3	6	4	4	6	5	6
2	1	4	1	3	1	2	1	4	3	3	4	3	5	5	3	4	3	5	5	4	4	4	4	4	2	4	3	3	1	1	3	3	3	3	4	5	3	2	3	3	3	7	4	4	6	6	3

1	7	2	1	3	5	3	1	4	5	5	5	5	5	4	3	4	5	5	5	5	5	4	4	4	3	3	3	4	5	5	3	3	3	3	3	3	3	4	5	5	5	4	5	3	4	3	4
2	4	2	1	1	2	3	1	5	5	4	4	4	4	4	3	2	3	4	5	4	3	2	3	4	5	4	3	4	5	4	3	4	5	4	3	4	5	4	3	4	5	6	5	6	7	6	4
2	1	4	1	1	1	1	1	5	4	5	3	4	5	4	3	5	4	5	3	4	5	4	5	3	5	4	3	4	5	4	3	5	4	3	5	4	5	4	3	5	4	7	6	5	4	7	5
2	1	2	1	3	1	1	1	4	4	3	4	5	4	5	3	4	4	4	3	3	2	4	4	4	2	5	3	4	5	4	3	3	3	2	2	2	2	2	3	2	2	5	6	4	4	4	3
2	6	2	2	1	5	2	1	4	4	5	4	5	5	4	3	2	4	4	5	4	5	4	4	5	3	4	3	4	4	5	3	4	5	5	3	3	4	5	5	4	5	7	6	7	7	7	5
1	1	2	1	3	1	1	1	4	2	3	4	2	3	5	5	3	3	5	5	5	5	4	4	4	1	3	3	4	4	5	3	3	3	3	3	3	4	5	4	4	4	4	4	3	4	5	2
1	1	2	1	3	1	3	1	5	5	4	5	5	4	4	5	4	4	5	3	5	4	5	4	4	2	5	3	4	4	4	3	3	4	4	4	3	4	3	3	5	4	4	6	6	6	6	4
2	2	2	1	3	1	1	2	5	4	3	4	4	4	4	5	3	4	4	2	3	4	4	4	5	4	4	3	4	4	4	3	4	4	5	4	5	5	4	3	4	4	6	5	4	5	6	1
2	2	2	1	3	2	4	2	5	4	5	4	3	4	4	4	4	2	3	3	3	3	3	2	2	1	2	3	4	4	4	3	3	3	2	1	1	2	1	1	1	1	4	3	3	4	5	4
2	2	4	1	3	1	1	1	5	4	3	3	4	4	4	3	3	4	4	4	3	4	3	3	3	3	3	3	4	4	4	3	4	4	3	3	3	3	3	4	4	4	4	4	4	5	5	1
1	1	2	1	2	1	1	1	5	4	3	4	2	4	5	5	5	3	3	4	3	4	4	3	3	2	3	3	4	4	4	3	3	3	4	3	3	5	1	2	4	3	6	6	6	5	6	3
1	2	2	1	3	2	3	1	4	4	4	4	5	5	5	5	5	4	4	4	4	4	3	3	3	3	3	3	4	4	4	3	3	3	3	3	3	3	3	3	3	4	3	6	3	4	4	1
2	2	2	1	3	2	2	2	3	4	4	4	4	4	4	4	4	4	4	4	3	4	4	4	4	3	3	3	4	4	4	3	3	3	3	3	4	3	4	3	4	3	4	4	5	6	5	5
2	2	2	1	3	5	2	1	1	2	3	4	3	3	3	3	3	5	3	4	1	3	3	4	4	3	4	3	4	4	4	3	4	4	4	5	5	3	4	4	3	3	6	5	6	6	5	5
2	1	2	1	3	1	1	1	5	5	5	5	5	4	5	4	4	5	5	4	5	5	5	5	4	3	5	3	4	4	3	3	3	5	5	5	5	5	5	4	3	5	5	6	7	6	5	2
1	1	2	1	3	1	4	2	3	5	5	5	3	4	3	3	4	4	4	4	4	3	3	4	3	4	3	3	4	4	3	3	3	3	3	3	3	4	4	3	3	3	5	4	5	4	5	3
1	3	4	1	3	3	3	2	4	3	4	3	4	2	5	2	2	3	3	2	4	4	3	3	3	3	3	3	4	4	3	3	3	3	3	3	3	4	4	3	3	4	5	5	5	5	5	3
2	1	2	1	1	1	1	1	4	4	3	5	4	3	5	5	4	3	5	4	3	5	3	4	4	3	5	3	4	3	5	3	5	4	4	5	3	5	4	4	3	5	5	4	6	4	5	3
2	1	2	1	3	1	1	1	3	4	2	5	5	4	5	4	3	3	3	3	2	2	2	3	3	4	3	3	4	3	5	3	2	3	3	4	4	2	3	3	3	3	5	4	5	4	4	2
1	2	4	1	3	5	2	1	4	4	3	3	3	2	3	4	4	3	4	4	4	4	3	5	4	4	5	3	4	3	4	3	4	3	4	5	3	5	4	5	4	4	6	5	5	3	6	4
1	1	2	1	3	1	1	1	4	3	3	4	3	3	3	3	4	4	4	3	3	4	3	3	3	4	4	3	4	3	4	3	3	4	4	4	4	4	4	4	3	3	5	4	5	4	4	6
1	2	2	1	2	1	2	1	3	3	3	3	4	5	4	3	4	4	4	4	4	4	4	4	4	5	4	3	4	3	4	3	4	4	4	3	3	3	3	4	4	3	5	4	5	5	5	4
1	2	2	1	3	3	2	1	3	3	3	4	4	3	3	3	3	3	3	3	3	3	4	4	3	3	3	3	4	3	4	3	4	4	5	4	3	4	4	4	3	2	3	3	3	4	4	1
1	2	2	1	3	1	3	1	4	4	4	4	4	4	4	4	4	4	4	4	4	4	3	3	3	4	3	3	4	3	3	3	3	4	3	3	2	2	2	3	3	3	4	5	4	4	4	4
1	2	4	1	3	2	1	1	4	4	4	3	4	3	4	3	4	4	3	3	4	4	4	5	4	3	4	3	4	3	3	3	4	3	3	4	3	4	4	5	3	3	6	5	5	5	4	4
2	2	2	1	3	1	1	1	5	3	3	3	4	3	5	5	4	4	5	3	3	3	3	5	3	4	3	3	4	3	3	3	3	4	4	3	3	3	3	4	3	4	5	4	4	4	4	3
1	2	2	1	3	1	1	1	5	5	4	4	3	4	5	5	5	5	5	4	3	5	3	3	3	3	3	3	5	5	5	3	3	3	4	4	4	4	2	2	1	1	5	7	7	7	7	3
1	5	2	1	3	2	3	1	4	3	4	2	4	3	2	2	3	4	5	5	5	5	5	5	5	3	4	3	5	5	5	3	3	4	4	3	4	5	4	3	5	2	5	5	5	5	4	5
2	1	2	1	1	1	1	1	4	4	3	5	4	5	4	3	3	3	5	3	4	5	4	4	4	3	3	3	5	3	4	3	5	3	4	3	3	3	5	5	4	3	6	5	3	5	4	2
2	1	2	1	2	1	4	1	5	4	5	4	3	3	4	4	4	4	2	4	3	3	4	4	4	4	4	3	2	4	4	4	4	4	4	4	4	3	2	2	3	3	5	5	6	5	5	4
1	6	4	2	3	6	3	1	4	3	4	4	4	4	2	2	2	4	4	4	2	2	4	4	4	1	2	3	2	4	4	4	2	2	2	2	2	4	4	4	4	4	6	2	6	6	5	5
1	5	2	1	3	3	1	1	4	4	4	4	5	5	5	4	5	5	5	5	5	5	3	3	4	3	3	3	2	4	3	4	2	3	3	3	3	2	2	2	2	4	4	5	4	4	6	4
1	2	2	1	2	1	3	1	3	4	3	3	4	3	4	2	2	5	5	3	3	3	2	3	4	2	2	3	2	3	5	4	4	2	2	4	4	4	4	4	3	3	4	4	3	3	6	3
2	2	2	1	3	1	3	1	4	4	3	5	3	4	4	4	4	3	2	2	3	1	2	2	2	3	2	3	2	3	3	4	4	2	4	3	3	3	3	4	4	3	7	5	5	5	5	2
1	2	2	1	3	1	4	1	4	4	4	4	4	4	3	3	3	4	4	4	4	4	4	4	4	4	4	3	2	2	2	4	4	4	5	3	3	4	4	5	4	4	7	5	5	7	7	6
2	2	2	1	3	1	1	1	3	4	4	4	2	3	4	2	3	3	2	3	2	3	3	3	3	2	3	3	3	5	5	4	2	2	3	2	2	2	1	2	2	3	4	5	3	4	4	2
2	7	2	1	3	3	1	1	5	3	5	3	3	4	3	2	3	4	2	3	2	3	3	2	2	1	1	3	3	5	5	4	1	1	1	1	1	4	5	5	5	5	1	1	1	1	1	4
2	2	2	1	1	1	2	1	2	2	2	2	3	3	3	3	4	4	4	4	3	5	3	2	2	1	1	3	3	5	5	4	1	1	1	1	1	1	4	4	4	4	2	7	2	3	3	1
1	2	2	1	2	2	3	1	5	5	5	5	4	4	4	5	4	4	4	4	4	5	3	3	3	3	3	3	3	5	5	4	4	3	3	3	2	2	1	1	1	5	5	7	4	7	6	1
2	2	2	1	3	2	3	1	4	4	4	3	4	4	4	4	4	4	4	4	4	4	4	4	4	3	4	3	3	5	5	4	4	4	4	3	3	3	4	4	4	4	5	6	6	6	6	4
2	4	2	2	2	1	3	1	3	3	3	3	3	3	2	2	4	3	4	3	3	3	5	5	5	3	4	3	3	5	5	4	3	2	3	4	5	4	2	3	3	1	6	6	5	6	5	1
2	1	2	1	1	1	1	1	5	5	4	4	4	3	4	4	3	4	4	3	4	3	4	4	4	3	4	3	3	5	4	4	3	3	4	4	3	4	4	4	3	4	6	5	5	4	6	2
1	2	2	1	3	1	1	1	4	5	4	4	5	5	4	3	3	4	4	4	3	3	4	4	4	4	5	3	3	5	4	4	4	4	4	4	4	3	3	3	3	3	6	6	6	6	6	4
2	2	2	1	1	2	3	1	5	4	4	3	4	4	5	5	4	5	3	5	4	4	5	4	3	5	4	3	3	5	4	4	4	5	3	5	4	5	4	5	3	4	7	6	5	6	7	5
1	2	1	1	1	1	3	2	3	4	4	4	3	3	3	2	3	5	5	5	5	4	3	3	3	3	3	3	3	5	4	4	4	4	4	4	3	4	3	3	3	5	3	4	4	4	4	1
1	1	4	1	3	2	1	1	4	4	3	3	5	5	3	4	4	4	3	3	5	4	4	4	5	4	4	3	3	5	3	4	4	4	5	5	5	5	5	5	5	5	7	6	7	5	5	4
2	3	2	1	1	1	3	1	3	3	4	3	5	4	3	3	3	4	4	3	3	3	4	3	4	4	5	3	3	5	3	4	3	3	5	3	4	3	4	3	3	1	6	4	3	6	5	3
1	2	2	1	4	2	3	1	4	4	4	4	4	4	4	4	2	4	4	5	5	5	4	5	5	5	5	3	3	5	2	4	3	3	3	3	3	5	5	5	5	5	7	5	4	7	7	6
2	1	2	1	3	1	1	1	3	2	3	3	4	4	3	3	3	5	3	4	3	5	3	3	3	1	1	3	3	4	5	4	2	2	1	2	2	1	1	4	2	2	4	5	1	4	4	1
2	4	2	1	3	2	3	1	5	5	5	3	5	5	5	5	5	5	4	4	3	3	5	5	5	5	5	3	3	4	5	4	2	3	3	1	1	5	2	3	2	3	7	7	7	7	7	4
1	2	4	1	3	1	1	1	5	5	5	5	5	5	5	4	4	5	4	5	5	5	4	3	3	3	4	3	3	4	5	4	4	4	4	4	4	4	4	4	5	4	7	6	5	5	6	4
2	3	4	2	3	1	1	1	3	5	5	3	5	2	2	1	1	4	5	4	3	4	5	5	5	3	3	3	3	4	5	4	4	5	5	3	3	5	5	5	5	5	7	7	6	7	7	5
1	1	2	1	3	1	1	1	5	4	5	4	5	4	4	3	4	5	4	4	3	3	4	3	3	3	3	3	3	4	5	4	3	4	4	3	3	2	2	2	2	3	4	6	3	4	4	2
1	2	2	1	3	1	1	1	5	5	4	4	3	3	4	4	4	4	4	5	3	4	4	4	4	5	5	3	3	4	4	4	4	4	4	4	4	2	2	2	3	3	6	5	5	5	5	3
1	2	2	1	3	1	1	1	4	5	4	4	3	3	4	3	3	4	4	5	3	4	4	4	4	4	4	3	3	4	4	4	4	4	4	4	3	2	2	3	3	3	5	5	4	5	5	3
1	1	1	1	3	1	4	1	5	4	4	3	4	4	4	4	4	4	5	4	4	4	4	4	4	4	4	3	3	4	4	4	4	4	5	3	3	4	3	3	3	3	6	6	6	6	6	5
2	1	2	1	3	1	3	1	4	3	5	4	4	4	3	4	4	4	3	4	4	4	4	4	5	4	4	4	3	4	4	4	5	5	5	5	5	3	3	5	4	4	5	4	6	5	6	3
2	4	2	2	2	2	3	1	4	4	3	4	3	4	3	4	3	4	4	4	4	4	4	3	3	3	3	3	3	4	3	4	3	3	3	3	3	3	3	3	3	3	4	6	5	4	5	3
2	1	4	1	1	1	2	1	4	4	4	5	4	5	5	4	4	3	4	5	4	4	3	5	4	4	3	3	3	4	3	4	4	3	4	4	3	5	4	4	4	4	4	6	6	6	6	2
2	1	2	1	3	1	2	1	4	4	3	4	3	4	4	4	4	3	2	2	2	3	4	4	4	4	4	3	3	4	3	4	4	4	4	4	4	4	2	2	3	3	6	6	6	6	6	1
2	5	2	1	3	2	3	1	4	4	4	3	2	4	4	2	2	4	2	4	3	2	4	4	4	2	4	3	3	4	3	4	2	4	4	4	4	3	5	3	3	4	6	6	5	5	6	2
2	2	2	1	3	1	3	1	4	4	4	4	4	4	4	4	4	3	3	3	3	3	5	5	5	5	5	3	3	4	3	4	4	4	4	4	4	4	5	5	5	5	7	7	7	7	7	4
1	4	2	2	3	3	3	2	3	4	2	3	5	4	3	4	3	2	5	4	3	4	3	4	2	5	4	3	3	4	3	4	3	5	3	4	4	2	1	2	4	4	5	5	6	6	6	3
2	7	2	2	2	4	3	1	4	3	5	4	3	4	4	3	2	4	4	4	3	4	4	4	4	4	4	3	3	4	3	4	4	4	4	3	4	4	3	4	4	4	6	5	6	6	6	6
2	2	2	1	2	2	3	1	3	3	4	4	5	3	3	3	4	4	4	4	2	4	4	3	4	3	3	3	3	4	3	4	4	3	3	3	4	3	3	3	3	4	5	5	4	4	5	3
2	2	2	1	3	1	4	1	3	3	3	3	3	3	4	4	3	3	3	4	3	4	2	3	4	3	3	3	3	4	3	4	3	3	3	3	3	3	2	3	4	3	4	4	5	4	4	3
1	1	2	1	3	2	1	1	3	3	2	3	4	4	3	3	3	4	4	4	4	4	3	3	4	4	4	3	3	4	3	4	3	3	3	3	3	4	4	2	3	3	5	5	4	4	3	2
1	2	2	1	3	1	2	2	4	3	2	3	4	3	2	3	5	5	4	3	4	4	4	3	3	3	4	3	3	3	5	4	5	5	4	4	4	4	5	5	4	5	4	6	5	7	7	3
2	2	2	1	3	2	1	1	4	5	4	3	4	4	3	3	4	4	4	3	4	4	5	4	5	2	2	3	3	3	4	4	4	4	4	4	4	4	5	4	4	4	6	6	7	6	6	4
1	2	1	1	2	1	3	1	4	4	4	5	4	4	3	4	4	3	4	4	3	4	3	3	3	3	3	3	3	3	4	4	4	3	4	4	3	3	2	3	3	4	4	6	5	4	4	4
2	2	2	1	3	1	1	1	3	4	4	4	5	5	4	3	3	4	4	4	4	4	4	4	4	4	4	3	3	3	4	4	4	4	4	4	4	3	3	4	3	2	6	6	5	6	6	6
2	1	2	1	3	1	1	1	3	2	5	3	1	4	4	3	2	4	4	3	3	3	4	3	4	4	4	3	3	3	4	4	4	4	4	4	4	3	4	3	4	3	5	5	4	5	5	6
2	5	2	2	4	2	3	1	5	5	4	5	5	5	4	4	3	5	5	5	4	5	4	4	4	4	4	3	3	3	3	4	4	4	4	3	3	4	4	4	4	5	5	6	6	6	6	5
1	7	2	2	2	3	3	1	5	5	5	5	4	5	4	4	4	4	4	4	4	4	5	5	5	5	5	3	3	3	3	4	4	4	5	4	4	4	5	5	5	5	7	6	6	7	7	4
1	2	2	1	3	1	2	1	4	5	4	3	4	3	3	3	2	5	4	3	4	2	2	2	3	3	3	3	3	3	3	4	3	3	3	3	4	3	4	3	3	3	4	5	5	6	5	1
1	2	2	1	3	1	4	1	5	4	5	4	5	5	5	3	5	3	4	5	4	4	4	4	3	4	3	3	3	3	3	4	5	4	3	3	3	3	5	4	5	5	7	6	5	7	6	3
1	3	2	1	3	2	2	1	5	4	3	3	3	4	3	2	4	4	4	3	3	4	5	4	4	4	4	3	3	3	3	4	2	2	3	3	3	4	5	4	4	4	6	6	6	6	6	5
2	1	2	1	1	1	4	2	4	4	3	3	5	5	4	4	4	3	5	4	4	3	4	4	4	2	4	3	3	3	3	4	4	4	4	4	4	3	3	3	3	2	5	5	3	4	6	1
2	7	2	2	3	3	3	1	4	4	4	4	4	4	4	4	3	4	4	4	4	4	4	4	4	4	4	3	3	3	3	4	4	4	4	4	4	3	3	3	3	3	4	4	5	5	5	4
1	8	2	2	1	1	2	1	4	4	4	4	4	4	4	3	3	4	4	4	4	4	4	4	4	4	4	3	3	3	3	4	4	4	4	4	4	3	3	3	3	3	5	5	5	5	5	4
1	2	4	1	2	2	2	1	4	4	3	3	2	3	3	4	3	4	3	3	3	3	4	3	4	3	3	3	3	3	3	4	3	3	3	4	3	3	3	3	3	2	6	6	5	5	4	2
1	3	2	1	3	3	3	1	4	4	3	4	2	3	4	4	3	4	4	4	3	4	4	4	4	2	2	3	3	3	3	4	5	5	5	3	3	4	4	4	4	4	6	6	6	5	6	5
1	3	2	1	3	4	3	1	3	4	4	4	5	4	4	2	2	4	4	4	3	3	4	4	4	2	3	3	3	3	3	4	4	4	3	2	2	3	4	2	3	4	4	5	3	3	4	1
2	2	2	1	1	1	3	1	4	3	3	3	4	3	4	2	3	3	3	3	3	3	3	2	3	2	2	3	3	3	3	4	3	3	3	4	4	2	2	2	2	3	4	4	4	4	3	2
1	1	2	1	1	1	1	1	4	3	3	4	2	3	3	3	3	4	4	4	4	4	4	4	4	4	3	3	3	3	3	4	3	3	3	2	2	3	2	2	3	2	6	6	5	6	6	4
1	1	2	1	3	1	2	1	4	3	3	4	2	2	3	3	3	3	3	5	3	4	5	5	5	3	4	3	3	3	3	4	3	3	4	3	3	3	3	3	3	3	6	5	5	6	6	6
2	8	2	2	2	1	1	1	3	3	4	3	4	3	3	3	3	3	3	3	3	3	4	4	4	4	4	3	3	3	3	4	3	3	3	2	2	3	3	3	3	3	6	3	3	6	6	2
1	2	2	1	1	2	3	1	3	3	3	3	3	3	3	3	3	3	3	3	3	3	4	3	4	3	3	3	3	3	3	4	3	4	3	3	4	3	4	4	3	4	5	5	4	4	5	1
2	4	2	1	1	3	3	1	3	3	3	3	3	3	4	3	3	3	3	4	4	3	3	3	3	3	3	3	3	3	3	4	3	3	3	3	3	3	3	3	3	3	4	4	4	4	4	4
1	2	4	1	3	2	1	1	3	3	3	3	4	4	3	3	3	4	2	4	4	4	3	3	3	3	3	3	3	3	3	4	3	3	3	3	4	4	4	4	4	4	3	4	4	3	4	2
2	1	2	1	3	1	1	2	5	4	4	4	5	4	4	5	4	3	4	5	5	5	5	5	5	5	5	3	3	2	5	4	5	5	5	4	4	4	2	4	4	3	5	3	4	3	5	4
1	9	2	2	3	2	3	1	4	5	4	3	4	4	5	5	5	5	5	5	5	5	5	5	5	4	5	3	3	2	4	4	4	4	4	2	3	4	5	5	5	5	5	4	5	7	5	3
2	2	2	1	3	2	3	1	3	4	3	4	3	3	4	2	2	3	4	4	3	3	5	4	4	2	3	3	3	2	4	4	4	4	4	4	3	3	3	3	4	3	4	6	5	5	5	4
2	9	2	1	4	4	2	1	5	5	5	5	5	5	5	5	5	5	5	5	3	3	4	4	4	5	4	3	3	2	3	4	4	4	4	1	1	5	5	5	5	5	7	5	4	7	7	4
1	2	2	1	2	1	1	1	3	3	3	3	3	4	2	4	4	3	3	3	3	5	2	2	2	2	2	3	3	2	3	4	3	3	3	4	3	3	3	4	3	4	3	4	5	4	5	1
1	2	4	1	3	1	1	1	5	5	4	5	5	5	5	5	5	4	3	3	3	3	2	2	1	1	3	3	4	5	5	4	2	1	1	3	1	4	5	5	5	5	4	5	3	3	3	1
2	2	2	1	3	1	1	1	4	4	5	3	4	3	5	4	3	3	4	4	5	4	4	3	4	3	4	3	4	5	5	4	3	4	3	4	4	3	4	3	4	3	5	5	4	4	6	4
1	2	2	1	1	1	1	1	4	4	4	5	4	4	5	4	4	4	3	3	4	4	5	4	5	2	3	3	4	5	5	4	3	4	5	4	3	3	5	4	5	4	6	6	6	5	6	6
2	1	2	1	3	3	3	1	5	4	5	4	3	4	5	4	3	5	5	5	3	5	4	4	4	5	5	3	4	5	4	4	4	4	5	4	4	4	3	2	2	3	7	7	4	6	7	2
2	8	2	2	1	3	3	1	5	3	2	4	5	3	3	4	4	5	4	5	4	5	2	2	3	2	3	3	4	5	4	4	3	3	2	2	4	5	4	4	3	2	3	5	5	6	4	3
2	1	2	1	1	1	4	1	4	3	4	2	2	4	5	3	5	5	5	5	4	5	4	4	4	5	3	3	4	5	4	4	2	2	5	4	4	5	5	4	5	5	7	7	6	7	7	5
1	2	2	1	3	1	4	1	3	4	5	3	4	4	4	4	4	2	2	3	2	3	4	4	4	4	4	3	4	5	3	4	3	4	4	3	3	2	3	3	4	4	6	7	4	5	6	2
2	3	2	1	2	2	3	1	4	3	4	5	4	3	2	3	4	3	2	5	3	4	3	4	5	4	4	3	4	5	3	4	4	5	3	3	4	3	2	3	4	3	5	5	4	6	5	4
2	2	2	1	2	2	3	2	5	5	5	5	5	5	5	5	5	4	4	2	2	3	4	4	4	3	3	3	4	4	4	4	4	5	5	4	4	2	4	4	3	3	3	3	4	4	4	1
2	2	2	1	2	1	1	1	4	5	4	4	3	3	2	2	3	4	4	5	5	5	3	3	3	3	3	3	3	4	4	4	4	4	4	3	3	2	3	2	2	3	5	5	5	6	5	3
2	8	2	2	2	3	3	1	4	4	5	3	4	4	4	4	4	4	4	4	3	4	5	3	4	4	4	3	4	4	4	4	3	3	4	3	3	4	4	4	3	4	5	4	5	6	6	6
1	3	4	1	3	5	1	1	3	4	3	4	3	4	3	3	4	4	3	3	3	4	4	4	4	3	3	3	4	4	4	4	3	3	3	3	3	3	4	3	4	3	4	4	5	4	5	4
1	1	2	1	2	1	4	1	3	3	3	3	3	3	3	3	3	3	3	3	3	3	3	3	3	3	3	3	4	4	4	4	4	4	4	4	5	4	3	4	3	4	5	5	4	4	5	2
1	7	2	2	2	1	3	1	3	3	3	3	3	3	3	3	3	3	3	3	3	3	2	3	3	3	4	3	4	4	3	4	3	3	3	3	3	3	3	3	3	4	4	3	4	5	4	3
1	2	2	1	2	1	1	1	5	5	5	4	4	3	5	4	4	5	3	4	5	4	5	5	4	4	5	3	4	3	5	4	4	4	5	4	3	5	5	5	5	4	5	6	6	6	6	1
2	1	2	1	3	1	1	1	4	4	5	5	4	5	5	4	4	4	3	4	4	4	4	4	4	4	4	3	4	3	4	4	4	4	4	3	3	4	2	3	4	2	6	6	6	7	6	5
1	2	2	1	2	2	3	1	4	4	4	3	2	3	2	4	4	3	5	5	5	3	4	2	4	3	5	3	4	3	4	4	2	3	2	4	3	5	5	3	5	5	7	7	5	7	7	4
2	1	2	1	2	1	1	1	4	3	5	3	4	4	5	4	4	4	3	4	4	4	4	4	4	3	4	3	4	3	4	4	3	3	4	3	3	1	2	1	2	2	4	6	2	5	5	4
1	2	2	1	1	1	4	1	4	3	4	4	5	4	4	3	4	5	5	4	4	4	5	5	4	4	2	3	4	3	4	4	3	4	4	4	3	5	4	4	5	4	5	6	6	5	5	2
1	2	2	1	2	6	5	1	3	3	3	3	3	3	4	3	3	3	4	3	3	4	3	4	3	3	3	3	4	3	4	4	4	4	3	3	4	4	3	3	2	4	6	3	4	5	4	6
2	3	2	1	3	2	2	1	4	4	5	5	5	4	5	3	3	4	4	4	4	5	5	5	5	5	5	3	4	3	3	4	3	4	3	3	3	3	4	3	3	3	5	5	5	6	6	4
1	2	1	1	3	2	3	1	4	4	4	4	4	4	4	3	3	3	4	3	3	4	3	3	3	4	4	3	4	3	3	4	3	3	3	4	4	3	3	4	4	3	5	5	6	6	5	4
1	2	2	1	2	1	3	1	3	3	3	3	3	3	3	3	3	3	3	3	4	3	3	3	4	3	3	3	4	3	3	4	3	3	4	3	3	3	4	3	4	3	5	4	5	3	5	3
2	2	2	1	1	1	3	1	5	5	3	3	4	3	3	4	3	3	3	3	3	3	4	4	5	4	4	3	4	2	4	4	4	2	2	2	1	2	2	3	2	2	2	4	4	6	5	4

1	2	2	1	1	2	3	1	5	4	3	3	4	2	5	4	5	2	2	5	5	4	4	3	4	3	5	3	5	5	4	4	4	3	4	3	5	4	3	5	5	3	6	5	7	4	7	4
1	2	2	1	3	3	3	1	4	4	3	3	5	5	5	5	5	5	4	3	5	5	4	3	4	3	5	3	5	5	4	4	4	4	4	4	3	5	4	3	3	4	4	7	5	5	6	3
1	2	3	1	3	1	2	1	5	4	4	4	5	5	4	4	4	4	5	5	4	4	4	4	5	4	5	3	5	4	4	4	4	4	5	4	4	5	3	3	3	4	4	4	4	3	4	1
1	1	3	1	1	1	1	1	5	4	4	3	5	5	5	5	5	1	1	1	1	1	1	1	1	5	3	3	5	4	4	4	1	1	1	1	1	5	5	5	5	5	1	1	1	1	1	1
2	2	2	1	1	1	3	2	4	3	5	3	4	3	4	5	3	5	3	4	4	4	4	3	5	3	5	3	5	3	5	4	4	5	3	3	5	4	4	5	4	5	5	7	5	6	5	2
2	1	4	1	3	2	3	1	4	5	3	4	2	5	5	5	4	5	5	5	4	3	5	5	5	5	5	3	2	5	4	5	3	3	5	5	5	5	4	5	3	3	4	5	6	5	7	2
2	2	2	1	1	1	3	2	2	1	2	2	5	5	4	3	2	5	4	5	3	4	4	5	5	4	3	3	2	2	3	5	5	4	4	5	5	4	4	4	3	4	6	6	6	7	7	3
1	2	4	1	1	1	1	1	4	4	3	4	4	3	3	3	2	4	3	4	3	2	3	3	2	4	4	3	3	5	3	5	4	4	4	3	2	5	4	4	3	4	4	6	5	3	5	3
1	9	2	2	3	6	3	1	5	4	5	5	5	5	5	5	5	5	5	5	4	5	5	5	5	5	5	3	3	5	5	5	5	5	5	5	5	5	5	5	5	5	7	7	7	7	7	6
2	6	2	2	2	3	1	1	4	3	5	1	4	5	5	4	4	5	5	5	4	4	4	4	5	3	5	3	3	5	5	5	5	5	5	3	3	3	4	5	3	5	7	7	4	7	5	6
1	3	2	1	3	2	3	1	4	3	4	3	4	4	4	4	4	5	3	3	3	3	2	2	2	5	3	3	3	4	3	5	2	2	2	2	2	3	2	3	4	4	5	5	5	5	5	2
1	1	2	1	3	1	1	1	3	5	5	4	5	4	5	5	5	5	3	3	2	4	5	4	4	5	5	3	3	4	5	5	5	3	5	3	3	5	5	5	2	4	7	5	6	7	6	6
2	3	2	2	3	3	3	1	4	4	5	4	5	5	5	5	5	3	3	4	3	4	5	5	5	5	5	3	3	4	5	5	4	5	5	5	5	3	4	3	4	5	7	7	6	7	7	6
2	4	2	1	3	3	3	1	3	3	3	4	5	5	4	3	3	4	5	3	4	3	5	5	5	5	5	3	3	4	5	5	4	3	5	2	2	3	4	4	5	5	6	7	3	6	6	5
1	2	2	1	3	1	3	1	5	5	4	5	4	5	5	5	4	4	3	3	3	4	5	5	5	5	5	3	3	4	4	5	5	5	4	3	3	3	4	4	4	4	6	5	7	5	6	4
1	2	3	1	1	1	1	1	5	5	5	4	5	5	5	5	5	5	5	5	5	5	2	2	2	2	2	3	3	3	5	5	1	1	1	1	1	1	2	1	2	2	1	3	3	2	3	2
2	1	2	1	2	2	3	1	5	4	3	3	5	5	5	3	4	3	4	5	5	4	3	2	2	2	2	3	3	3	5	5	2	2	1	2	1	1	2	2	2	5	7	7	4	6	5	1
2	1	2	1	1	1	3	1	3	3	3	3	4	4	5	3	3	3	4	5	2	2	4	4	4	3	3	3	3	3	3	5	2	2	2	2	2	1	1	1	3	3	2	4	2	4	4	2
1	2	2	1	1	1	3	1	5	5	5	5	3	1	3	2	3	3	3	5	3	3	5	5	5	5	5	3	3	3	3	5	5	4	5	5	5	5	3	5	5	3	7	5	5	5	5	6
2	2	4	1	3	2	3	1	5	5	5	5	5	5	2	2	1	5	3	4	3	4	5	5	5	5	5	3	3	3	3	5	5	5	5	5	5	5	4	4	4	4	7	7	7	7	7	1
2	5	2	2	3	4	2	1	3	5	4	4	3	3	4	2	3	5	4	5	2	4	5	5	5	5	5	3	3	3	3	5	4	5	4	5	5	4	2	3	2	2	6	5	4	6	7	5
2	2	2	1	1	1	3	2	4	3	3	3	3	3	3	3	3	3	3	3	3	3	3	3	3	4	3	3	3	3	3	5	3	3	3	4	3	3	3	3	3	3	4	4	4	4	5	4
2	3	2	1	2	2	2	1	4	3	3	3	4	5	3	4	4	5	5	3	3	4	3	3	3	1	3	3	3	2	3	5	1	1	1	1	1	2	4	3	3	5	4	4	6	4	4	1
1	2	3	1	3	1	3	1	5	5	5	4	5	5	4	4	5	5	5	5	4	5	5	5	5	5	5	3	3	1	5	5	5	5	5	5	5	5	5	5	5	5	7	7	7	7	7	4
2	1	2	1	3	4	2	1	3	3	3	3	3	3	3	3	3	3	3	3	3	3	3	3	3	3	3	3	4	5	5	5	5	4	5	4	4	5	5	5	5	5	7	6	7	6	6	1
2	7	2	2	1	1	3	1	5	5	5	3	5	5	3	4	4	4	4	4	4	4	5	5	5	5	5	3	4	5	1	5	5	5	5	3	3	3	5	3	4	4	7	7	5	5	5	3
1	2	2	1	3	2	2	1	5	3	4	3	3	5	5	1	3	1	1	3	4	1	4	4	4	3	3	3	4	4	2	5	3	3	3	1	1	1	1	1	3	3	4	5	4	4	4	3
1	2	2	1	2	2	3	1	1	1	2	3	3	3	3	4	3	3	2	3	3	4	4	4	2	3	3	3	4	4	2	5	3	3	1	3	3	3	2	3	3	4	3	4	3	3	4	1
2	2	2	1	3	1	1	1	4	3	4	5	5	4	5	5	4	5	4	5	5	4	5	5	5	5	5	3	4	4	5	5	5	4	4	5	4	5	4	4	4	4	6	6	5	7	6	5
1	2	2	1	3	1	1	1	5	5	5	4	4	4	5	5	4	5	4	5	4	3	4	4	3	4	3	3	4	4	4	5	5	5	4	4	3	4	5	5	5	5	6	5	5	5	5	1
1	6	2	2	3	2	3	1	4	5	5	4	5	5	5	4	3	4	4	4	3	3	5	5	5	5	5	3	4	4	4	5	4	5	5	5	5	3	3	4	5	4	7	7	7	6	6	5
1	2	2	1	3	2	3	1	4	5	5	4	4	5	4	4	5	5	4	4	3	4	5	5	4	5	5	3	4	4	4	5	5	4	3	4	4	4	5	4	4	5	6	7	7	5	6	4
2	3	2	1	4	2	4	1	4	4	5	3	4	5	4	5	4	4	5	5	5	4	3	5	5	4	4	3	4	4	4	5	5	4	5	5	5	5	5	5	5	4	6	5	7	7	7	2
1	1	2	1	2	1	1	1	3	4	4	4	4	4	3	4	4	3	4	4	3	3	4	4	3	3	3	3	4	4	4	5	5	3	4	4	4	3	4	4	4	4	4	4	5	6	4	4
2	6	2	2	1	2	2	2	4	3	5	5	5	5	4	5	5	4	4	5	5	4	3	3	3	2	3	3	4	4	4	5	4	4	5	3	3	3	3	2	4	4	4	5	4	4	5	4
2	5	2	2	3	2	3	1	4	3	5	5	5	5	4	5	5	4	4	5	5	4	3	3	3	2	3	3	4	4	4	5	4	4	4	5	3	3	3	2	4	4	4	5	4	4	5	4
2	1	2	1	3	1	1	1	3	5	5	5	5	5	5	5	5	4	5	5	3	5	5	5	5	4	4	3	4	4	3	5	5	5	5	5	5	3	2	3	3	3	5	5	6	4	5	6
2	1	2	1	1	1	1	2	4	3	5	4	3	5	5	4	3	3	4	5	5	4	5	3	4	5	4	3	4	4	3	5	5	4	3	3	5	4	4	5	3	3	6	6	5	5	5	1
1	1	4	1	3	2	4	1	5	4	4	4	5	4	5	4	5	5	3	4	5	5	5	5	5	4	5	3	5	5	5	5	5	5	5	5	3	4	4	5	4	5	5	6	6	7	7	6
1	1	2	1	3	1	1	1	5	5	4	3	3	3	3	3	3	4	2	3	3	4	3	3	3	1	3	4	2	5	4	2	1	3	3	4	3	4	3	3	2	3	3	5	4	3	3	3
2	1	2	1	3	1	1	1	5	5	5	5	5	4	4	2	2	3	4	4	4	4	2	2	2	3	2	4	2	4	4	2	2	2	2	2	2	2	1	1	1	3	5	6	2	5	5	1
2	1	2	1	2	1	4	1	4	3	3	4	3	2	4	3	3	3	4	3	2	4	4	2	3	4	4	4	2	4	3	2	2	3	3	3	4	3	3	3	2	2	6	6	2	6	5	2
1	2	2	1	3	3	3	1	4	4	5	3	4	4	4	4	4	4	4	2	2	3	4	2	4	1	1	4	3	5	4	2	2	3	4	3	3	4	4	4	5	5	6	6	2	6	6	4
2	2	2	1	3	2	2	1	4	4	5	3	4	5	2	5	3	4	4	4	3	4	5	4	5	5	3	4	3	4	5	2	4	5	3	4	4	3	2	3	4	4	6	7	5	6	7	5
1	2	2	1	3	1	1	1	4	4	5	2	3	3	2	2	1	3	2	4	3	2	4	5	4	2	3	4	3	4	4	2	2	2	3	2	3	2	3	2	3	3	2	3	3	4	5	1
1	2	4	1	3	1	2	1	5	3	5	3	5	5	4	2	2	5	5	5	4	4	4	2	4	2	2	4	3	4	4	2	2	3	2	3	3	4	4	3	4	3	5	3	4	5	5	1
2	3	2	1	3	2	3	1	3	3	5	3	4	5	4	2	4	4	4	4	3	4	3	3	3	2	2	4	3	4	4	2	2	2	2	1	1	3	3	3	3	4	3	2	2	2	2	2
1	3	4	1	3	1	1	1	5	5	5	5	5	5	4	5	5	3	4	4	4	4	5	5	5	4	5	4	3	4	3	2	2	5	5	4	5	5	5	5	5	5	7	7	7	7	7	1
2	2	2	1	2	1	1	1	4	3	5	4	4	3	3	2	3	4	4	5	4	4	3	3	3	5	3	4	3	3	4	2	3	2	2	2	2	3	2	2	2	3	5	5	3	4	4	2
2	2	2	1	3	1	4	1	3	3	3	3	2	3	3	3	3	4	4	5	4	5	3	4	3	2	2	4	3	3	4	2	3	3	3	2	2	2	2	3	2	3	6	7	5	6	6	3
1	2	4	1	1	1	1	1	3	4	2	5	2	5	3	2	4	1	5	3	3	2	4	2	3	5	1	4	3	2	5	2	5	2	5	4	3	3	2	5	3	1	6	4	3	7	5	1
1	2	2	1	3	2	1	1	5	2	4	2	4	5	5	5	5	5	5	5	3	5	3	3	3	3	3	4	3	2	5	2	2	4	3	3	3	4	2	2	1	3	4	6	6	5	5	3
1	1	4	1	1	1	1	1	5	4	5	3	4	5	4	3	4	3	3	3	4	4	2	2	2	1	2	4	4	5	5	2	2	3	2	3	3	2	2	1	2	2	3	5	2	1	2	1
2	2	2	1	3	2	3	1	4	4	4	3	3	4	3	3	3	5	5	5	4	4	3	3	4	3	3	4	4	5	5	2	3	3	3	3	3	2	2	1	3	2	4	7	3	3	4	1
2	2	2	1	3	2	3	1	4	4	5	3	4	4	3	3	3	4	4	3	4	3	2	2	2	1	2	4	4	5	5	2	2	3	2	2	2	3	2	2	3	4	2	6	2	2	2	2
2	2	4	1	4	5	3	1	4	4	3	2	5	5	5	4	4	4	3	1	2	2	1	1	1	1	1	4	4	5	5	2	2	2	1	2	2	1	3	1	2	2	1	5	2	1	1	1
2	2	4	1	2	1	1	1	4	4	4	4	3	4	4	3	4	4	5	5	3	4	3	4	4	1	2	4	4	5	4	2	2	2	2	2	2	5	5	5	5	4	3	4	2	3	3	1
2	1	2	1	3	1	1	1	3	4	4	4	4	4	3	3	2	4	4	4	3	4	3	3	4	2	1	4	4	5	4	2	1	1	2	2	2	3	3	2	3	4	4	5	2	3	4	2
2	3	2	2	4	1	4	1	2	4	5	3	4	4	5	2	2	4	4	5	5	5	2	2	3	2	3	4	4	5	4	2	2	2	2	2	2	2	2	2	2	2	4	4	3	4	4	1
2	6	1	2	5	6	3	1	3	3	4	3	4	4	5	4	4	4	4	4	5	5	3	3	3	2	3	4	4	5	4	2	2	2	2	3	3	1	3	2	3	4	2	3	3	2	2	1
1	2	4	1	3	1	1	1	3	2	4	3	4	4	5	3	3	5	4	4	3	4	1	1	2	1	1	4	4	5	4	2	1	1	1	3	2	2	1	3	1	5	1	4	1	1	1	1
1	2	2	1	3	1	1	1	3	2	4	2	4	4	2	2	2	3	2	2	3	2	3	2	3	1	1	4	4	5	3	2	2	2	2	2	2	2	3	2	4	4	2	3	2	2	2	4
2	2	2	1	3	2	3	1	4	4	3	3	2	5	5	3	4	3	2	3	3	3	4	3	2	2	2	4	4	4	5	2	3	2	3	2	2	2	1	2	3	3	5	6	3	5	6	1
2	2	2	1	3	1	1	1	5	5	4	5	3	2	3	2	3	3	2	3	5	4	3	4	4	5	5	4	4	4	4	2	4	5	4	4	5	5	5	4	5	3	5	5	7	6	6	3
1	2	2	1	3	1	3	1	4	5	3	4	3	4	4	4	4	4	5	5	5	5	2	2	2	2	1	4	4	4	4	2	2	2	2	2	2	2	3	4	4	4	3	4	4	3	3	2
1	2	2	1	1	1	1	1	4	4	5	5	5	5	5	5	4	5	5	4	4	4	3	3	4	2	3	4	4	4	4	2	2	2	2	2	2	2	2	1	2	2	4	7	4	4	4	2
1	1	2	1	2	2	2	1	4	4	4	4	4	4	4	2	2	4	4	4	4	5	4	4	5	2	2	4	4	4	4	2	4	4	4	4	4	4	3	5	3	2	5	5	5	6	6	4
1	2	2	1	3	1	1	1	4	4	4	3	5	5	2	2	1	5	4	4	2	4	3	4	3	2	2	4	4	4	4	2	2	2	1	2	2	2	2	3	1	4	3	6	3	4	5	1
2	2	2	1	3	1	1	2	3	4	4	4	4	4	5	4	4	3	3	4	3	3	4	3	3	2	2	4	4	4	4	2	2	2	2	4	3	2	2	2	2	2	4	6	3	4	4	1
2	4	2	1	3	6	3	1	4	3	4	3	4	5	2	1	2	3	3	3	3	3	3	3	2	2	3	4	4	4	4	2	2	2	2	2	2	4	4	4	4	5	6	5	2	3	4	4
2	1	2	1	3	1	4	1	4	3	3	3	4	3	3	3	3	4	4	4	3	4	4	4	4	3	3	4	4	4	4	2	2	2	2	2	2	3	2	3	2	3	5	6	4	6	6	3
1	1	2	1	2	1	1	1	4	2	4	3	4	4	5	5	4	3	4	4	4	3	4	2	3	4	5	4	4	4	4	2	3	3	4	3	4	3	5	4	4	4	5	6	6	6	5	5
2	6	2	2	3	1	1	1	3	2	4	2	4	4	3	3	2	4	4	4	3	4	3	3	3	2	2	4	4	4	4	2	2	2	2	2	2	3	3	3	3	3	5	4	3	4	4	4
2	2	2	1	3	1	4	1	4	3	5	4	4	4	4	5	3	2	3	3	3	3	3	3	3	2	3	4	4	4	2	2	3	3	2	2	3	5	2	3	3	3	5	5	5	5	5	2
2	2	2	1	3	1	4	1	3	3	3	3	3	3	3	3	3	3	3	3	2	3	2	2	4	2	2	4	4	3	4	2	3	2	2	3	3	2	3	3	3	2	4	6	4	4	4	1
1	2	2	1	3	1	1	1	5	5	4	5	4	4	4	4	3	4	4	4	3	3	2	2	2	3	3	4	4	3	3	2	2	2	3	4	3	2	2	2	2	2	4	3	3	3	3	1
1	2	2	1	3	2	3	1	1	1	1	2	5	5	4	2	2	2	2	4	2	2	4	2	4	1	4	4	4	2	4	2	2	2	2	2	2	2	2	4	2	4	2	6	2	4	4	3
2	3	4	2	3	2	4	1	4	5	4	3	3	4	2	2	3	5	5	5	4	5	3	3	4	2	3	4	4	2	3	2	2	3	3	3	4	2	2	2	3	4	5	2	1	5	6	1
1	2	2	1	3	1	3	1	2	4	4	3	5	5	5	4	4	4	4	5	4	5	3	3	4	2	2	4	5	5	5	2	2	2	3	2	2	5	5	5	5	5	5	4	6	5	6	4
2	3	2	1	3	3	3	1	5	4	4	4	5	4	3	4	2	5	4	3	3	4	2	3	2	2	2	4	5	4	5	2	2	2	2	3	3	4	3	4	4	3	3	5	3	3	2	2
1	2	4	1	3	2	1	1	5	4	3	4	5	5	5	3	3	3	3	4	4	4	4	3	4	4	5	4	5	4	3	2	1	1	1	1	1	3	5	3	4	5	6	4	4	4	5	1
2	2	2	1	3	1	1	2	5	4	5	1	4	5	4	4	3	4	5	3	2	5	4	4	4	5	2	4	2	5	4	3	4	5	5	1	1	1	1	2	1	2	6	7	1	7	5	1
1	2	2	1	3	1	1	2	4	4	4	4	3	3	4	4	4	4	4	4	4	4	4	4	4	4	4	4	2	2	4	3	4	4	4	3	3	4	4	4	4	4	6	6	6	6	6	2
1	2	2	1	1	1	3	1	4	3	4	5	2	4	5	5	2	4	5	3	5	4	5	4	3	5	5	4	3	5	5	3	2	3	2	1	1	2	1	2	1	3	6	4	3	4	6	2
2	2	2	1	3	1	4	2	4	4	4	4	4	4	4	4	4	4	4	4	4	4	4	4	4	4	4	4	3	5	4	3	5	5	5	4	4	4	4	4	4	4	5	6	6	6	5	4
2	6	2	2	1	1	4	1	4	4	3	5	4	3	5	4	5	3	4	5	4	4	3	2	4	3	2	4	3	5	4	3	5	4	4	5	3	5	4	4	5	4	6	5	7	4	6	4
1	1	2	1	3	1	2	1	3	3	4	3	3	3	4	2	2	3	4	4	3	3	2	2	2	2	2	4	3	5	3	3	3	3	3	3	3	3	5	3	4	5	2	4	3	4	4	2
1	2	4	1	3	1	1	1	5	5	5	4	3	4	3	3	4	3	2	3	4	3	4	4	5	5	5	4	3	4	5	3	4	5	5	3	3	2	3	4	3	2	6	7	7	7	7	1
2	2	2	1	2	1	3	1	4	4	3	3	5	3	4	3	4	4	4	3	3	4	4	5	3	5	4	4	3	4	5	3	4	4	3	4	5	4	4	3	3	4	5	4	6	7	5	3
2	1	2	1	1	1	1	1	4	4	4	3	4	5	4	4	4	3	3	4	3	3	4	4	4	4	4	4	3	4	5	3	3	4	4	4	5	5	4	4	5	4	7	6	6	6	6	4
2	1	2	1	3	1	1	1	5	5	4	3	4	3	3	3	4	3	3	4	3	3	4	3	4	2	2	4	3	4	4	3	3	3	3	2	4	2	4	3	3	3	6	3	5	3	6	6
1	2	2	1	3	2	1	1	5	4	2	5	4	5	4	4	4	2	4	5	3	3	2	5	5	4	3	4	3	4	4	3	5	4	4	4	4	5	3	2	5	5	6	5	6	7	6	4
1	4	2	1	3	3	3	1	5	4	4	5	3	4	3	3	3	3	2	2	3	4	4	4	5	3	4	4	3	4	4	3	3	4	3	4	3	3	4	3	3	4	5	5	4	4	5	3
1	2	4	2	3	2	2	1	5	4	5	3	5	5	4	4	4	3	4	3	3	3	4	4	4	1	3	4	3	4	4	3	3	4	3	3	3	3	3	3	3	4	4	6	4	5	5	1
1	4	2	1	3	2	2	1	4	4	4	4	4	4	4	4	4	4	4	4	4	4	3	3	3	3	3	4	3	4	4	3	3	3	3	3	3	4	4	4	4	4	4	4	4	4	4	4
2	3	2	1	2	2	3	1	4	4	3	4	3	5	4	3	4	5	4	3	4	3	3	3	3	2	3	4	3	4	4	3	4	3	4	3	5	4	3	3	4	3	5	6	5	4	5	4
1	1	2	1	1	1	1	1	3	4	3	4	4	4	4	4	4	4	3	3	3	3	3	3	3	3	3	4	3	4	4	3	3	3	3	3	3	3	3	3	3	3	4	4	4	4	4	3
2	7	2	2	1	2	2	1	4	3	4	4	5	4	4	3	3	4	4	5	4	5	3	4	4	3	3	4	3	4	4	3	2	3	3	3	3	2	2	3	2	3	5	6	4	5	4	2
1	3	2	1	1	3	3	1	4	3	5	4	3	3	2	3	4	3	3	4	3	5	4	3	4	2	3	4	3	4	4	3	4	4	3	4	3	4	4	3	3	4	5	4	5	5	6	4
1	1	2	1	3	3	3	2	2	1	1	1	4	4	4	4	3	4	5	4	4	5	3	4	3	5	5	4	3	4	4	3	5	4	4	3	5	4	4	5	4	4	6	6	7	6	5	4
2	1	2	1	3	1	1	1	5	5	5	5	5	5	5	5	5	4	3	3	4	4	4	3	4	4	4	4	3	4	3	3	3	5	5	5	5	5	5	5	5	5	5	5	5	5	5	4
2	1	2	1	2	1	3	1	3	4	3	4	3	3	3	3	4	3	4	3	3	3	3	3	3	3	3	4	3	4	3	3	4	2	5	3	3	4	3	4	3	3	5	4	4	4	4	3
1	2	2	1	1	2	3	1	3	4	3	3	3	3	3	3	4	3	4	4	3	4	4	3	3	3	3	4	3	4	3	3	3	3	3	3	3	3	3	3	3	3	4	4	4	5	4	4
2	1	2	1	1	1	3	1	5	4	4	4	4	5	5	5	3	3	3	3	3	3	3	3	3	2	2	4	3	3	5	3	2	2	2	2	2	1	1	1	2	3	3	5	2	2	2	1
2	2	2	1	3	1	4	1	4	4	4	4	4	4	5	5	5	4	3	4	4	5	5	5	5	5	5	4	3	3	5	3	5	5	5	3	3	4	4	5	5	5	7	5	6	6	7	5
1	2	2	1	2	1	3	1	4	4	4	3	2	2	4	3	3	4	4	4	4	5	5	4	5	4	3	4	3	3	4	3	3	3	3	4	4	3	3	3	3	3	4	4	4	5	5	3
2	1	2	1	1	1	4	1	3	4	3	3	4	3	3	3	2	4	4	4	3	3	4	4	3	4	3	4	3	3	4	3	3	2	3	3	4	3	2	3	2	3	4	3	4	3	5	3
2	2	2	1	3	6	2	1	4	3	3	3	4	3	3	3	4	3	3	3	4	4	3	5	3	4	3	4	3	3	4	3	3	4	3	4	3	4	3	3	3	4	4	4	4	4	4	4
1	2	1	1	3	1	1	1	4	3	4	4	4	4	4	4	3	3	3	4	4	3	3	3	4	3	4	4	3	3	4	3	4	3	3	3	3	4	3	3	3	3	5	4	5	5	5	4
2	7	2	2	3	3	2	1	4	4	5	2	5	5	4	5	3	5	5	5	3	4	5	4	4	3	3	4	3	3	3	3	1	1	2	3	2	3	3	4	5	3	5	2	3	5	6	6
2	2	2	1	3	1	1	1	4	4	3	5	4	3	4	4	3	4	3	3	4	4	3	5	4	4	4	4	3	3	3	3	3	4	3	3	4	3	3	3	3	4	4	4	5	5	5	2
2	1	2	1	2	1	4	1	4	4	3	3	4	3	3	4	3	3	4	3	4	3	3	3	4	3	3	4	3	3	3	3	3	3	3	4	3	3	4	3	3	3	4	4	4	4	4	2
1	2	2	1	2	1	3	1	3	4	3	3	4	4	4	3	3	4	4	4	4	4	4	3	3	3	3	4	3	3	3	3	4	3	3	3	3	4	3	3	3	3	4	5	6	5	5	3
1	2	2	1	3	1	1	1	3	4	3	3	4	4	4	3	3	4	3	3	3	3	3	3	3	3	3	4	3	3	3	3	3	3	3	3	3	3	3	3	3	3	4	4	4	4	4	1
1	2	2	1	1	2	3	1	3	3	3	3	3	3	3	3	3	3	3	3	3	3	4	3	3	3	3	4	3	3	3	3	3	3	3	4	3	3	3	4	3	4	5	5	5	5	5	4
2	3	2	1	3	2	3	1	5	4	4	5	4	5	5	5	5	5	4	4	4	3	2	1	1	2	1	4	3	2	5	3	3	2	5	2	5	3	5	5	5	5	6	6	6	7	6	1
2	1	2	1	2	2	3	1	4	4	3	4	1	1	3	4	4	5	5	5	5	5	3	3	3	4	5	4	3	2	5	3	2	3	4	4	4	5	5	4	3	5	5	5	6	6	6	4
2	1	4	1	1	1	2	1	5	4	3	3	5	5	4	2	3	5	5	5	4	5	4	3	4	3	3	4	3	2	4	3	4	4	5	3	3	4	2	3	4	3	4	6	5	6	7	4
2	2	3	1	2	2	3	1	3	4	3	4	3	3	3	4	2	4	3	4	2	4	3	4	3	3	5	4	3	2	4	3	4	2	4	3	3	3	5	3	3	2	5	5	4	4	4	2

1	2	2	1	3	1	1	1	4	5	4	4	4	5	5	2	2	4	4	4	4	4	4	4	4	2	4	4	4	5	5	3	2	2	2	2	3	3	2	2	2	5	6	6	5	5	5	3
1	2	1	1	3	2	3	2	4	5	5	3	4	3	4	1	4	4	4	4	3	4	4	4	4	4	4	4	4	5	5	3	4	3	3	3	3	2	1	2	2	5	6	6	5	5	5	2
1	2	2	1	2	1	3	1	4	5	3	4	5	3	5	3	2	4	5	5	3	4	4	4	4	2	4	4	4	5	5	3	3	3	3	3	3	4	2	3	4	5	5	6	5	5	5	1
2	2	2	1	2	1	3	1	4	5	3	3	4	4	5	2	4	3	3	3	3	3	4	4	4	2	3	4	4	5	5	3	3	3	3	2	2	2	1	1	1	2	4	6	3	4	4	2
2	2	2	1	3	1	1	1	4	5	4	3	5	5	3	4	5	4	4	4	4	3	4	4	5	3	2	4	4	5	5	3	4	5	4	3	3	2	4	4	3	4	6	6	4	6	6	3
2	2	2	1	3	1	3	1	5	4	5	4	5	5	4	4	3	4	5	4	4	4	3	4	3	2	2	4	4	5	5	3	3	2	2	3	3	1	2	2	2	2	4	6	3	4	3	1
2	2	2	1	3	3	3	1	4	4	4	3	4	5	5	3	4	5	4	3	4	3	4	3	4	3	4	4	4	5	5	3	3	3	3	5	3	4	3	2	3	4	6	5	2	6	6	4
2	1	2	1	3	1	1	1	4	4	3	2	5	5	3	2	3	3	2	1	3	2	3	3	4	3	2	4	4	5	5	3	3	4	4	3	3	2	2	1	2	1	5	5	3	5	5	1
2	3	4	3	3	2	3	1	4	4	4	3	5	4	3	2	2	4	5	4	5	4	5	5	5	3	3	4	4	5	5	3	3	3	3	4	4	4	4	3	4	5	5	6	5	6	6	4
1	2	2	1	2	1	3	1	3	4	3	3	4	4	4	4	5	3	3	3	3	3	3	3	2	1	2	4	4	5	5	3	2	2	2	2	2	2	1	1	2	2	4	5	3	4	4	2
1	1	4	1	1	1	1	1	3	4	5	3	5	5	4	5	4	3	2	1	3	2	2	2	2	2	2	4	4	5	5	3	3	2	3	3	3	1	1	1	1	2	5	4	2	2	4	1
2	1	2	1	2	1	2	1	4	3	5	4	4	5	3	3	3	4	5	5	5	4	3	3	3	3	3	4	4	5	5	3	3	3	3	3	3	4	1	2	2	4	4	4	4	4	4	1
1	2	4	1	3	2	3	1	4	3	5	3	5	5	5	4	3	4	5	4	4	5	4	4	3	3	4	4	4	5	5	3	4	3	3	2	2	4	5	4	5	4	6	7	4	7	7	4
1	7	2	2	1	2	2	1	4	3	5	3	4	4	3	3	3	5	4	5	5	5	4	3	4	2	3	4	4	5	5	3	2	2	2	3	3	3	2	3	2	2	4	5	4	3	3	2
1	1	2	1	1	1	1	1	5	5	5	4	4	4	4	5	2	4	4	4	4	5	4	5	4	3	3	4	4	5	4	3	3	3	4	3	3	4	2	2	2	3	5	6	5	6	6	4
1	1	2	1	2	1	1	1	5	5	4	4	4	5	5	4	4	4	3	3	3	5	4	3	3	2	3	4	4	5	4	3	2	4	3	4	4	2	3	3	3	2	5	4	5	4	4	3
1	2	4	1	2	1	1	1	5	5	4	4	5	5	4	3	3	4	4	4	3	4	5	5	5	4	4	4	4	5	4	3	3	4	3	3	3	4	4	4	4	4	5	6	6	6	6	3
1	3	2	1	3	1	4	1	3	5	5	3	5	5	4	3	3	4	3	3	3	3	3	2	3	2	3	4	4	5	4	3	3	3	2	4	2	2	3	2	3	3	3	5	3	3	2	1
2	3	1	1	5	1	2	1	4	4	2	3	4	4	4	3	4	4	4	4	4	4	5	4	4	4	4	4	4	5	4	3	3	4	4	4	4	2	2	2	3	3	5	6	4	5	5	1
2	2	2	1	2	2	3	1	3	4	5	4	4	3	4	5	4	3	2	2	3	3	4	4	4	3	3	4	4	5	4	3	2	3	3	3	3	2	3	2	3	4	6	5	6	6	6	3
2	1	4	1	2	1	1	1	3	4	3	3	5	4	5	4	3	4	5	4	4	5	3	3	4	3	3	4	4	5	4	3	3	2	2	3	3	3	3	3	4	3	5	7	5	7	5	3
2	2	2	1	3	2	3	1	5	3	4	4	4	5	4	3	3	4	3	3	4	4	5	3	5	4	3	4	4	5	4	3	3	3	4	3	3	3	2	3	3	3	5	5	5	5	5	4
1	3	2	1	4	3	3	2	4	3	4	3	4	4	2	2	2	2	2	2	2	2	2	2	3	1	2	4	4	5	4	3	3	3	3	2	2	2	3	2	3	4	2	2	2	3	3	1
2	8	2	2	5	4	3	1	4	3	5	4	5	5	4	2	3	5	4	5	4	4	5	3	5	4	5	4	4	5	4	3	4	5	5	4	4	4	4	2	4	4	6	7	6	6	7	6
1	5	2	1	3	2	3	1	3	3	4	3	4	4	3	2	2	3	4	4	3	4	4	4	4	3	4	4	4	5	4	3	4	4	4	4	4	3	4	3	3	4	6	6	5	5	6	4
1	2	2	1	3	2	2	1	3	4	4	3	5	5	4	2	2	3	3	4	3	3	3	3	3	3	3	4	4	5	3	3	3	3	3	3	3	3	3	3	4	4	5	5	4	5	5	3
2	1	2	1	3	1	4	1	4	3	3	3	5	4	2	3	2	2	5	4	4	4	2	2	4	3	3	4	4	5	2	3	5	4	4	4	4	3	2	3	3	4	6	7	5	6	6	2
2	2	2	1	3	2	3	1	2	1	4	2	5	5	5	3	4	2	2	1	2	3	4	3	3	1	3	4	4	4	5	3	2	2	2	1	1	1	2	5	4	3	4	5	3	2	4	1
2	2	2	2	3	2	3	1	4	5	4	4	3	4	4	4	4	4	4	5	4	4	4	4	4	3	3	4	4	4	5	3	2	3	3	2	2	2	2	2	2	2	5	5	4	5	5	3
2	2	2	1	3	1	1	1	5	5	4	3	5	5	4	3	3	4	4	4	4	3	3	3	3	3	3	4	4	4	5	3	5	3	3	3	3	2	2	2	2	4	4	7	2	4	4	1
1	1	4	1	2	1	1	1	5	5	4	3	5	5	3	2	2	3	3	4	4	2	2	4	2	1	3	4	4	4	5	3	3	3	3	4	4	4	2	4	1	1	1	3	3	3	5	1
2	2	2	1	3	2	3	2	4	4	4	4	4	4	3	4	3	5	5	5	4	5	4	3	4	1	3	4	4	4	5	3	3	3	3	3	3	2	2	2	2	4	4	5	4	4	5	4
2	1	2	1	1	1	2	1	4	4	5	5	4	4	3	3	4	5	5	5	5	5	4	4	4	4	4	4	4	4	5	3	3	3	3	3	3	3	3	3	4	3	4	5	4	4	5	3
1	2	2	1	2	1	4	1	4	4	5	4	4	3	5	5	4	5	4	3	4	5	4	3	4	5	4	4	4	4	5	3	5	4	5	4	4	3	5	5	4	5	7	6	6	5	6	2
1	9	2	2	2	6	3	1	4	3	4	3	4	4	3	2	3	4	5	4	4	4	3	3	3	1	2	4	4	4	5	3	2	2	2	1	1	2	2	3	4	4	4	3	4	3	3	4
2	2	2	1	3	1	3	1	5	5	3	3	3	2	4	4	4	3	2	5	3	3	4	4	4	3	3	4	4	4	4	3	3	3	4	4	4	2	2	2	2	2	3	5	4	4	4	1
1	7	2	2	4	4	3	1	5	5	5	4	3	4	4	4	3	5	5	5	5	4	1	1	1	1	3	4	4	4	4	3	3	3	3	3	3	2	4	4	3	4	4	4	4	3	3	2
1	2	2	1	3	2	3	1	5	5	5	5	5	5	5	4	5	4	5	5	5	5	3	4	4	2	3	4	4	4	4	3	3	3	3	2	2	4	4	3	4	4	3	4	4	4	4	3
2	7	2	2	4	4	3	1	5	5	5	5	5	5	5	5	4	5	5	4	4	5	5	5	4	3	3	4	4	4	4	3	3	3	3	3	3	4	5	5	5	5	6	6	5	7	6	5
2	2	2	1	3	2	3	1	4	5	5	5	4	4	4	4	4	4	4	4	3	4	3	3	3	3	3	4	4	4	4	3	3	3	3	3	3	3	3	3	4	4	5	5	5	5	5	2
2	1	2	1	2	1	3	1	4	5	3	3	2	4	5	4	2	5	1	1	2	3	5	5	5	5	5	4	4	4	4	3	4	3	4	5	3	3	3	4	4	4	5	5	6	5	5	3
1	8	2	2	3	6	3	1	4	5	4	4	5	5	5	5	4	4	3	3	4	4	5	4	5	3	4	4	4	4	4	3	3	3	4	4	4	4	4	4	4	4	6	6	5	6	6	6
1	1	1	1	3	1	1	1	5	4	3	3	5	4	5	4	3	3	2	2	3	2	5	5	5	3	2	4	4	4	4	3	3	3	3	3	3	2	2	2	1	2	4	3	3	3	3	1
1	1	2	1	1	3	3	2	4	4	4	2	3	3	5	4	3	1	3	2	5	5	4	4	4	5	5	4	4	4	4	3	3	5	3	3	4	3	3	5	2	4	5	5	4	4	4	4
2	1	2	1	2	1	3	1	4	4	4	3	5	4	5	5	5	4	5	5	5	5	3	3	3	4	4	4	4	4	4	3	3	4	4	3	4	4	4	4	4	5	6	6	6	5	5	3
2	5	2	2	2	2	3	1	4	4	5	4	4	4	4	2	2	4	4	4	4	4	4	4	4	2	3	4	4	4	4	3	3	3	3	2	2	3	3	4	4	4	4	6	4	4	3	3
1	2	2	1	3	1	1	1	4	4	4	4	5	5	4	2	2	3	3	3	3	3	3	3	3	3	3	4	4	4	4	3	3	3	3	3	3	4	3	3	3	4	4	4	3	4	4	2
1	7	1	2	4	6	3	1	4	4	4	4	4	4	4	3	3	3	3	3	3	3	3	3	3	2	3	4	4	4	4	3	3	3	3	3	3	3	3	3	3	3	4	5	4	4	4	3
2	1	2	1	3	1	3	1	4	4	5	3	4	4	5	4	4	4	3	4	4	4	4	4	4	3	4	4	4	4	4	3	3	3	3	3	3	3	2	3	4	4	5	6	6	5	6	2
1	3	3	1	3	2	3	1	4	4	4	3	4	4	4	3	4	2	2	3	3	3	3	2	4	3	2	4	4	4	4	3	3	3	3	3	3	3	2	2	3	4	4	5	4	4	4	1
2	4	4	2	4	6	3	1	4	4	5	3	4	4	5	5	4	4	5	4	4	3	5	5	5	5	3	4	4	4	4	3	3	4	5	2	2	3	1	1	1	5	7	7	7	7	7	4
2	2	2	1	3	2	2	1	4	4	4	4	3	3	3	3	3	3	4	4	4	4	4	3	3	3	3	4	4	4	4	3	3	3	3	3	3	3	3	3	3	3	4	4	4	3	4	1
1	1	2	1	3	1	1	1	3	4	4	4	5	4	4	4	4	3	4	4	4	5	3	3	3	3	3	4	4	4	4	3	4	4	3	3	5	4	4	4	4	4	5	5	4	5	5	2
1	3	4	1	3	2	3	1	3	4	4	3	4	4	4	4	3	4	4	3	3	4	3	3	3	2	3	4	4	4	4	3	3	3	3	3	3	3	3	2	3	3	3	4	4	3	4	1
2	2	4	1	3	1	1	2	5	3	5	2	2	4	5	3	3	5	4	4	5	5	4	4	4	1	4	4	4	4	4	3	4	4	4	3	3	4	4	3	4	3	5	7	4	5	5	6
2	1	2	1	3	1	3	2	4	3	3	3	2	2	2	2	3	4	4	5	5	4	3	3	4	3	3	4	4	4	4	3	3	3	3	3	3	3	2	2	2	5	5	5	5	5	4	4
2	4	2	1	3	3	3	1	4	3	4	3	4	5	3	3	3	4	4	3	4	5	3	3	3	2	3	4	4	4	4	3	2	2	2	2	2	3	4	4	4	3	5	4	4	4	4	3
1	2	2	1	3	3	3	1	4	3	4	4	4	4	5	4	4	4	5	4	4	4	3	3	3	2	2	4	4	4	4	3	3	3	3	3	3	4	5	4	5	4	4	6	4	4	4	3
1	1	1	1	1	1	1	1	4	3	3	3	4	4	5	4	4	4	4	4	4	3	4	3	3	2	2	4	4	4	4	3	3	3	3	3	3	2	2	2	1	3	3	4	2	4	4	1
2	1	2	1	3	1	1	1	4	3	3	4	4	3	5	5	4	3	3	3	3	3	4	4	4	3	3	4	4	4	4	3	3	3	4	3	3	2	1	1	2	2	4	6	4	4	4	1
2	2	2	1	3	1	1	1	4	3	4	4	3	4	4	3	3	3	4	4	3	4	3	3	3	3	3	4	4	4	4	3	3	3	3	3	3	3	3	2	3	3	3	4	4	4	4	2
2	2	2	1	1	1	4	1	4	3	3	4	5	3	4	4	4	2	5	3	4	4	4	4	4	3	3	4	4	4	4	3	3	3	4	3	4	2	2	2	2	2	4	4	3	4	4	1
1	2	4	1	3	1	2	1	4	3	5	4	2	3	2	3	1	4	5	3	4	4	4	2	4	4	3	4	4	4	4	3	4	3	5	3	4	4	2	1	1	3	4	4	3	5	5	2
2	1	2	1	3	1	1	2	3	3	4	3	4	4	4	3	3	4	4	4	4	4	3	3	4	2	3	4	4	4	4	3	3	3	3	3	3	3	3	3	3	3	4	6	4	5	4	1
2	2	2	1	3	1	3	1	3	3	4	3	4	4	4	3	3	4	4	4	4	4	3	3	3	3	3	4	4	4	4	3	3	3	3	3	3	2	2	2	2	4	5	4	4	5	5	1
2	2	2	1	3	1	1	1	3	3	4	4	5	5	5	2	4	5	5	5	5	5	4	4	4	4	4	4	4	4	4	3	3	3	3	3	3	2	1	1	1	1	5	6	5	4	4	2
2	2	2	1	3	1	1	1	4	2	4	3	4	4	4	3	3	4	4	3	3	3	3	2	3	2	3	4	4	4	4	3	3	3	3	3	3	2	2	4	4	4	4	5	4	5	5	4
2	5	2	2	2	1	4	1	2	2	2	2	2	2	2	2	2	4	4	4	4	4	3	3	3	3	3	4	4	4	4	3	3	3	3	3	3	5	5	5	5	5	5	5	5	5	5	5
2	2	2	1	3	1	3	1	5	4	5	2	4	2	3	5	5	5	3	4	5	4	4	3	2	3	5	4	4	4	3	3	4	3	5	2	4	4	2	2	3	5	5	5	4	6	6	2
1	9	2	2	1	2	2	2	4	4	3	4	3	4	4	3	3	3	4	4	3	3	5	4	4	3	4	4	4	4	3	3	3	5	3	5	4	4	3	3	4	4	4	5	5	4	4	4
1	2	2	1	3	2	3	1	4	4	5	4	5	5	5	4	4	5	4	4	4	5	4	4	3	3	4	4	4	4	3	3	3	4	5	4	4	4	3	4	5	4	5	5	6	5	5	3
1	2	2	1	3	1	4	1	4	4	3	4	3	4	4	4	3	2	3	2	2	2	3	2	3	2	3	4	4	4	3	3	3	2	3	3	3	3	4	4	3	4	5	4	5	5	4	2
1	2	2	1	3	1	1	2	3	4	4	4	4	4	4	4	4	4	4	4	3	3	3	3	3	3	2	4	4	4	3	3	2	2	2	2	2	3	3	4	3	4	4	6	3	4	4	3
2	3	4	1	3	1	1	1	4	3	4	4	3	3	3	4	3	5	5	5	5	5	5	5	5	5	4	4	4	4	3	3	4	3	4	4	3	3	4	3	4	3	6	6	6	6	7	1
1	1	2	1	1	1	4	1	5	5	4	3	3	5	2	4	3	3	3	4	3	5	2	3	3	3	3	4	4	3	5	3	3	3	3	3	3	2	2	2	3	3	4	7	4	5	5	4
2	2	2	1	3	1	1	1	4	3	4	4	5	4	4	4	4	4	4	5	4	4	4	4	4	2	3	4	4	3	5	3	3	3	3	3	3	3	3	3	3	5	5	7	5	5	5	3
1	4	2	2	4	2	3	1	5	4	4	4	4	4	3	3	3	4	4	4	3	4	3	3	3	1	2	4	4	3	4	3	4	4	4	4	4	3	3	3	2	4	3	4	3	3	3	3
1	2	4	1	3	1	2	1	5	4	5	3	4	3	3	4	4	3	4	4	4	3	4	3	3	4	3	4	4	3	4	3	4	3	3	3	3	4	4	3	4	3	4	4	3	4	4	3
1	5	2	1	1	1	4	2	4	4	3	4	4	3	3	3	3	4	4	3	4	3	4	4	4	3	3	4	4	3	4	3	4	4	3	4	4	3	4	4	4	3	5	6	5	5	4	6
2	2	2	1	3	1	3	1	4	4	4	4	3	3	4	4	4	3	3	3	3	3	4	4	4	3	4	4	4	3	4	3	3	4	3	3	3	3	3	2	3	2	6	6	5	5	5	4
1	1	2	1	3	1	1	1	4	4	3	4	4	4	3	4	3	3	4	3	4	3	3	4	4	4	3	4	4	3	4	3	3	4	4	4	3	3	4	3	4	3	5	4	4	4	5	1
2	2	2	1	3	1	1	1	5	3	4	3	4	4	4	2	4	4	4	4	3	4	4	3	4	3	4	4	4	3	4	3	3	3	4	3	3	2	4	2	4	2	6	6	3	6	6	3
1	9	2	2	2	2	2	2	4	3	4	3	5	5	4	3	3	5	4	4	4	4	4	3	4	3	3	4	4	3	4	3	3	3	4	3	3	4	3	4	4	4	5	6	4	5	5	4
2	1	2	1	3	1	1	1	4	3	5	3	3	4	5	2	2	5	4	3	3	4	3	3	3	1	2	4	4	3	4	3	3	3	3	2	2	4	4	2	4	3	5	6	3	6	6	2
2	7	2	2	2	1	2	1	4	3	4	3	5	4	4	3	3	5	4	4	4	5	4	3	4	3	3	4	4	3	4	3	3	3	4	4	3	3	4	3	4	4	5	6	4	5	5	4
1	1	2	1	1	1	1	1	3	3	4	5	4	4	4	5	4	3	3	3	4	3	5	4	4	4	4	4	4	3	4	3	3	5	4	4	4	4	4	3	4	3	4	3	3	5	5	3
1	7	2	2	1	2	2	1	3	3	3	3	3	3	3	3	3	3	4	4	3	4	4	3	4	3	3	4	4	3	4	3	3	3	3	2	2	3	2	3	2	3	5	5	5	5	5	3
1	6	2	2	3	4	2	1	5	5	4	4	5	5	5	4	4	5	4	3	3	3	4	4	4	4	3	4	4	3	3	3	3	3	3	4	4	4	4	4	3	4	5	5	5	6	4	4
1	1	1	1	3	1	1	1	5	4	5	3	2	3	3	2	3	3	4	4	3	4	4	5	4	4	4	4	4	3	3	3	3	3	3	4	3	3	3	3	3	3	4	4	5	4	4	2
2	2	2	1	3	1	1	1	5	4	4	4	4	5	4	4	3	4	2	4	3	4	4	3	4	2	2	4	4	3	3	3	4	4	4	4	4	4	4	3	4	3	5	5	4	6	6	5
2	1	2	1	1	1	3	1	3	4	3	3	3	3	3	4	4	4	5	4	3	5	3	3	3	3	3	4	4	3	3	3	5	3	3	3	3	3	3	3	3	3	4	4	4	5	4	2
2	2	2	1	3	2	3	1	4	3	4	4	3	4	3	3	4	4	3	3	4	3	3	3	3	3	4	4	4	3	3	3	3	3	3	3	4	3	3	3	3	3	4	4	4	4	4	3
2	1	2	1	3	1	1	1	4	3	5	4	5	4	5	5	3	4	4	5	4	4	5	5	5	2	4	4	4	3	3	3	4	4	3	2	2	4	2	2	2	2	5	5	4	5	4	4
1	1	4	1	3	1	1	1	4	3	4	3	4	3	3	2	5	5	4	4	4	4	4	4	3	3	3	4	4	3	3	3	4	4	3	3	3	3	3	4	4	4	5	5	5	5	6	1
2	1	2	1	1	1	1	1	4	3	3	2	4	3	4	4	2	4	3	4	3	4	3	2	3	2	3	4	4	2	5	3	2	4	2	3	3	4	1	3	2	4	6	4	3	5	4	3
2	4	2	1	3	4	3	1	5	5	5	4	4	4	4	4	2	5	5	4	1	4	4	4	4	2	4	4	4	2	4	3	3	3	3	3	3	1	1	2	1	5	7	7	7	7	7	4
1	7	2	2	2	2	2	1	5	4	4	2	4	4	4	3	3	4	4	4	4	4	4	4	4	3	4	4	4	2	4	3	3	3	3	3	3	3	2	3	3	2	4	5	5	5	5	1
1	2	1	1	3	1	2	1	4	3	5	4	5	5	2	5	5	4	5	4	5	5	4	3	4	2	2	4	4	2	4	3	3	3	4	5	5	4	4	2	1	5	6	7	6	5	5	1
2	2	4	2	4	1	2	1	3	3	2	2	2	5	4	4	2	5	3	3	3	5	5	4	4	4	5	4	4	2	3	3	3	3	4	4	4	4	4	3	3	3	4	4	5	6	4	3
2	1	1	1	2	1	2	2	4	5	5	5	5	5	5	4	3	2	2	3	2	3	4	5	3	2	4	4	5	5	5	3	2	3	3	3	3	1	2	2	2	2	2	6	4	5	6	2
1	1	1	1	1	1	1	1	4	4	5	3	5	5	3	3	5	5	5	5	4	4	4	4	4	4	4	4	5	5	5	3	3	3	3	3	3	4	3	3	3	2	5	7	4	6	6	3
1	2	4	1	3	2	3	1	3	4	5	3	5	5	5	4	4	3	4	5	4	2	4	4	4	3	5	4	5	5	5	3	3	3	4	3	3	4	5	4	5	4	6	7	5	6	6	5
2	2	2	1	3	2	3	1	4	3	5	3	4	4	4	2	3	4	3	4	4	4	4	4	4	5	5	4	5	5	5	3	3	3	3	4	4	3	3	3	3	4	5	6	5	6	6	1
1	2	2	1	1	4	3	2	4	4	4	4	4	5	4	5	5	5	5	5	5	4	5	5	5	5	5	4	5	5	4	3	5	5	5	5	5	4	5	4	4	5	6	6	6	6	6	2
2	1	2	1	3	1	1	1	4	2	3	4	4	4	4	5	5	4	4	4	4	4	3	4	4	3	4	4	5	5	4	3	4	3	4	4	4	3	2	4	4	4	5	6	6	4	6	2
2	3	2	1	4	3	3	1	5	4	5	4	4	4	5	3	4	5	5	5	3	5	5	4	4	3	3	4	5	5	3	3	4	4	4	3	3	2	2	3	2	4	6	5	6	6	6	3
2	1	2	1	2	2	3	2	5	4	3	2	5	4	4	4	4	2	2	2	4	4	4	3	4	1	2	4	5	4	5	3	4	5	4	3	4	3	3	2	3	4	5	6	5	5	5	4
2	2	2	1	3	1	1	1	3	2	2	4	5	5	4	4	5	5	5	5	5	5	3	2	3	3	2	4	5	4	5	3	3	3	3	3	3	5	2	2	3	5	4	6	3	5	5	3
1	2	2	1	2	2	2	1	1	1	1	1	4	4	2	3	3	5	5	5	5	5	4	4	4	4	4	4	5	4	5	3	4	4	4	4	5	3	3	2	3	5	6	7	5	6	5	4
2	2	2	1	1	1	3	1	4	4	3	3	4	3	4	3	3	4	4	4	3	3	4	4	4	3	3	4	5	4	4	3	3	3	3	3	3	5	3	3	4	3	6	7	4	6	6	2
2	4	2	1	1	2	2	1	4	4	4	3	5	3	4	5	4	4	5	5	4	4	5	4	4	4	4	4	5	3	3	3	3	3	3	3	3	4	3	4	4	4	5	5	4	5	4	4
2	1	2	1	2	1	3	1	2	5	4	4	4	3	3	4	4	3	3	5	4	5	5	5	5	5	5	4	5	3	2	3	3	4	3	4	3	3	5	5	5	3	7	3	7	7	7	5
1	2	2	1	3	1	1	1	4	5	4	5	4	5	5	4	4	4	4	3	3	4	3	5	4	5	4	4	3	5	4	4	3	5	4	4	4	5	4	4	4	3	7	5	5	5	6	4
1	2	2	1	1	1	1	2	4	5	3	3	5	5	5	5	3	3	2	3	3	3	4	4	4	4	5	4	3	5	3	4	3	4	3	4	4	4	4	4	4	4	5	5	5	4	4	6
2	2	2	1	3	1	1	1	4	5	4	4	5	4	4	4	5	3	4	4	4	3	4	4	3	4	4	4	3	4	4	4	3	3	5	4	4	4	4	3	4	4	5	6	4	5	5	2
1	2	2	1	3	1	1	1	5	4	4	5	4	5	5	4	3	5	4	4	5	5	4	5	5	4	2	4	3	4	4	4	4	4	4	3	3	4	4	5	5	4	6	7	5	7	7	5
1	1	2	1	2	1	1	1	5	4	4	3	5	4	5	4	4	5	5	5	4	4	4	4	4	4	4	4	3	4	4	4	4	3	3	4	4	4	4	4	4	3	5	6	7	6	6	4
1	3	2	1	1	1	3	1	4	4	3	4	4	3	3	4	4	4	3	4	4	3	4	3	3	4	4	4	3	4	4	4	3	4	3	4	3	3	3	3	4	4	5	4	5	5	5	3
2	2	2	1	3	1	1	1	4	4	4	3	4	4	4	4	4	3	4	4	3	4	4	3	4	3	4	4	3	4	4	4	4	4	4	4	4	4	4	4	4	4	5	5	5	6	5	1

1	3	1	2	3	3	3	1	4	4	4	3	4	4	4	4	4	3	4	4	3	4	4	4	4	4	4	4	3	4	4	4	4	4	3	4	4	4	4	3	4	4	6	4	6	6	5	4
2	2	2	1	2	2	3	2	5	5	4	5	4	5	3	4	4	4	4	3	5	4	5	4	4	5	3	4	3	4	3	4	3	5	5	5	3	4	4	3	4	5	6	7	5	5	4	3
2	2	2	1	3	1	4	1	5	5	3	3	2	4	3	2	2	3	3	2	1	2	5	5	5	5	4	4	3	4	3	4	5	4	5	3	3	3	2	2	2	5	4	4	4	6	5	1
2	5	2	2	1	2	3	1	5	4	4	3	4	5	4	3	4	3	5	4	4	4	3	5	4	3	4	4	3	4	3	4	3	4	4	3	4	3	4	4	3	5	5	4	6	5	6	4
2	2	2	1	2	2	3	1	4	4	3	3	4	4	3	4	3	4	3	4	3	4	3	4	3	4	3	4	3	4	3	4	3	4	3	4	3	3	3	3	4	3	4	4	5	5	5	4
1	2	2	1	2	1	2	1	3	4	4	3	3	3	4	3	4	3	4	3	4	3	4	3	4	3	4	4	3	4	3	4	4	4	4	3	4	4	4	3	5	4	6	4	6	5	5	2
1	2	4	1	3	3	4	1	3	4	4	3	3	4	3	4	3	4	3	3	3	5	3	4	3	3	3	4	3	4	3	4	3	3	5	4	5	5	5	5	4	5	5	6	6	6	6	4
1	2	1	1	2	4	2	1	4	3	2	5	2	5	4	1	5	4	4	3	3	4	3	5	2	4	2	4	3	4	2	4	3	4	3	4	4	4	4	4	4	4	3	6	5	6	6	3
2	1	2	1	1	1	4	1	3	3	3	3	4	3	3	3	3	4	4	4	4	4	4	4	4	3	3	4	3	3	5	4	4	4	4	3	3	3	3	3	3	4	6	6	5	6	6	4
1	2	2	1	3	1	4	1	4	5	4	4	4	4	3	3	4	5	4	5	4	4	4	4	4	4	5	4	3	3	4	4	4	3	4	3	4	4	4	4	3	3	6	5	4	4	6	3
1	1	2	1	2	1	1	1	4	4	4	3	5	4	4	3	4	4	3	4	3	3	2	2	3	3	4	4	3	3	3	4	3	4	3	3	3	4	3	4	4	4	4	5	5	4	5	3
2	1	2	1	1	1	3	1	3	4	3	3	3	3	3	3	3	4	3	3	3	3	3	3	3	3	3	4	3	3	3	4	3	4	3	3	3	3	3	3	3	3	4	4	4	4	4	2
2	2	2	1	3	1	1	2	2	1	2	2	2	3	3	2	1	3	4	5	4	4	2	2	3	2	1	4	3	3	3	4	3	3	5	5	4	3	2	2	3	1	6	7	5	6	5	3
2	1	2	1	1	1	3	1	4	5	3	4	3	2	4	4	3	2	3	4	3	4	5	5	4	4	4	4	3	2	4	4	3	3	4	4	4	2	3	5	4	3	6	6	6	6	6	4
1	2	2	1	3	1	3	1	5	4	4	4	2	3	2	2	2	2	2	2	2	2	4	4	4	4	4	4	3	2	4	4	4	4	3	3	4	2	3	3	3	4	5	5	6	6	6	4
2	6	2	2	3	3	2	2	5	5	4	5	5	5	4	4	4	5	5	4	4	4	5	5	5	4	4	4	3	2	3	4	3	3	3	3	3	3	3	4	3	2	6	6	7	6	7	6
1	2	2	1	3	1	2	1	5	5	5	5	5	5	5	5	5	5	5	5	5	5	5	5	5	5	5	4	4	5	5	4	2	2	2	2	2	2	2	2	2	2	5	7	5	6	5	1
2	4	2	1	3	3	3	1	5	5	5	4	4	5	5	5	5	5	5	4	5	5	4	5	5	4	4	4	4	5	5	4	4	4	3	3	4	4	4	4	4	4	6	5	6	5	5	4
2	8	2	2	3	3	3	1	5	5	5	5	4	5	4	4	3	5	5	5	2	5	4	4	4	2	4	4	4	5	5	4	4	4	4	4	4	4	5	4	5	5	5	7	4	5	5	3
2	2	2	1	3	1	3	1	5	4	4	4	4	4	4	4	4	4	4	5	5	4	4	4	4	4	4	4	4	5	5	4	4	4	4	4	4	4	4	4	4	4	4	5	4	4	4	2
2	2	1	1	2	1	4	1	5	4	4	4	4	5	3	4	4	4	4	3	5	4	5	4	5	4	4	4	4	5	5	4	5	5	4	5	5	5	5	5	4	4	6	6	6	3	3	3
2	2	2	1	2	1	3	1	5	4	5	4	5	5	5	4	4	5	5	4	4	4	3	3	4	2	4	4	4	5	5	4	4	4	4	3	3	2	2	2	1	1	4	4	3	3	3	3
1	4	2	2	1	2	3	1	5	4	4	4	4	4	5	4	4	4	4	5	4	5	5	5	5	5	4	4	4	5	5	4	3	4	4	3	3	4	4	3	4	4	6	6	6	6	6	4
2	4	2	2	2	2	3	1	5	4	5	4	5	5	5	4	5	5	4	4	5	5	5	5	5	5	4	4	4	5	5	4	4	4	4	4	4	4	4	4	4	4	7	7	6	6	6	5
1	2	1	1	1	1	4	1	5	4	5	5	4	4	5	3	3	4	4	3	4	3	5	4	5	3	4	4	4	5	5	4	4	4	5	3	3	3	2	3	2	2	5	5	4	5	6	1
1	4	2	1	3	2	3	1	4	4	5	5	5	5	5	5	5	4	4	4	4	4	3	3	3	3	3	4	4	5	5	4	3	3	3	3	3	5	5	5	5	4	5	7	4	6	7	4
1	2	2	1	3	4	3	1	4	4	5	5	5	5	5	3	4	4	4	3	4	4	4	4	4	3	4	4	4	5	5	4	3	4	4	3	3	2	2	3	3	1	3	6	4	4	4	3
1	1	2	1	3	2	3	1	4	4	3	3	5	5	4	2	2	4	4	5	3	4	4	4	4	3	3	4	4	5	5	4	4	3	4	4	4	4	4	4	4	5	5	6	4	5	5	4
2	2	2	1	3	2	3	1	4	4	3	5	5	3	5	4	4	3	3	3	4	4	4	3	2	2	5	4	4	5	5	4	1	4	4	3	3	2	2	2	4	3	6	7	6	7	6	6
1	2	1	1	3	1	1	1	3	4	4	3	4	4	4	3	3	5	5	5	5	5	5	5	5	5	4	4	5	5	4	5	5	5	4	5	5	5	5	5	5	5	7	7	7	7	7	2
2	2	2	1	3	1	1	1	3	4	5	3	4	3	4	4	4	3	2	4	3	4	4	3	4	4	4	4	4	5	5	4	4	4	4	3	3	2	2	2	2	2	4	5	5	5	5	5
2	8	2	2	1	2	3	1	4	3	5	4	2	4	5	3	4	4	5	4	4	5	5	5	5	4	5	4	4	5	5	4	5	5	5	4	4	4	2	3	2	4	7	7	5	7	7	6
2	2	2	1	3	1	4	1	5	5	4	4	4	4	5	5	5	4	5	5	4	4	4	4	4	4	4	4	4	5	4	4	4	4	4	4	4	2	3	4	3	4	6	6	6	6	5	3
1	9	2	2	3	6	3	1	5	5	5	5	4	4	5	3	5	5	5	5	5	5	5	5	5	5	5	4	4	5	4	4	4	4	4	3	3	5	5	4	4	4	6	5	5	6	5	5
2	2	2	1	3	1	1	1	4	5	5	4	5	4	4	4	4	3	4	4	4	4	5	4	4	5	5	4	4	5	4	4	5	5	5	4	4	2	2	2	1	2	4	6	5	7	7	6
2	1	2	1	1	1	3	1	4	5	4	5	3	5	4	4	4	5	5	4	4	5	3	4	4	5	5	4	4	5	4	4	5	4	5	4	5	4	4	5	4	4	5	5	6	6	5	6
1	1	2	1	3	1	1	1	4	5	4	4	4	4	4	5	5	5	3	5	4	3	3	4	4	5	5	4	4	5	4	4	5	5	4	3	4	5	4	5	5	5	5	6	4	6	5	4
2	1	2	1	1	1	3	1	4	5	5	4	4	4	5	3	5	4	4	4	4	4	4	5	4	5	5	4	4	5	4	4	3	2	2	2	2	4	4	3	3	4	4	5	4	4	5	4
2	1	2	1	3	1	1	1	5	4	5	4	4	3	4	4	3	5	5	4	4	5	4	4	4	3	4	4	4	5	4	4	4	4	4	3	3	2	2	3	2	3	5	5	4	5	5	4
2	1	2	1	3	3	3	1	5	4	5	3	4	5	5	4	4	5	5	4	3	4	5	5	5	5	4	4	4	5	4	4	3	3	4	4	4	3	3	3	3	4	6	6	5	6	6	3
1	8	2	2	3	4	3	1	4	4	4	4	5	5	5	4	4	4	4	5	4	4	4	4	4	4	4	4	4	5	4	4	4	4	4	4	4	4	4	4	4	4	6	6	6	6	6	4
2	2	2	1	3	2	3	1	4	4	4	2	4	4	4	1	4	2	3	2	2	3	4	4	4	2	4	4	4	5	4	4	5	4	4	4	4	2	2	2	2	4	6	5	6	6	6	6
2	6	2	2	1	2	2	1	4	3	4	3	3	3	3	3	4	4	4	5	4	4	4	4	5	4	5	4	4	5	4	4	3	3	4	4	4	4	4	3	4	3	6	5	5	5	6	4
2	3	2	1	3	2	3	1	4	3	5	4	4	4	3	1	1	4	3	4	5	3	5	5	5	5	5	4	4	5	4	4	2	4	5	2	3	2	2	3	4	5	6	6	6	6	6	6
2	2	2	1	3	1	1	2	2	1	2	2	4	3	4	2	3	4	3	4	4	4	4	4	4	4	4	4	4	5	4	4	5	4	4	4	3	3	3	4	3	4	4	5	5	4	5	4
2	4	2	1	3	3	3	1	5	5	5	5	5	5	4	3	4	5	4	4	3	4	4	4	4	3	4	4	4	5	3	4	3	3	3	4	4	4	4	4	4	4	3	6	3	4	5	3
2	1	2	1	3	1	1	1	4	2	4	4	4	4	4	4	5	4	5	5	5	5	5	4	3	3	3	4	4	5	2	4	5	5	3	3	4	2	4	4	4	3	6	5	5	5	4	4
2	1	2	1	3	1	1	1	5	3	3	2	2	3	5	2	2	4	4	5	4	4	2	1	3	1	1	4	4	4	5	4	1	2	1	1	1	4	3	4	4	5	4	6	3	3	2	1
2	2	2	1	3	1	1	1	4	3	4	4	3	4	4	3	4	4	2	2	2	2	1	1	1	1	1	4	4	4	5	4	1	1	1	1	1	1	1	1	1	2	2	7	3	2	3	1
1	1	2	1	2	1	2	1	5	5	4	5	5	5	5	4	4	4	5	5	2	5	5	5	5	5	5	4	4	4	5	4	4	5	4	5	4	4	4	3	5	5	7	6	6	7	6	4
1	5	2	2	3	3	3	1	4	5	4	4	5	5	5	4	5	5	4	5	5	5	5	5	5	5	5	4	4	4	5	4	4	4	5	4	4	4	4	5	3	5	6	6	5	5	4	6
1	2	2	1	3	1	1	2	5	4	4	5	5	5	5	4	5	5	5	5	5	5	4	4	4	1	5	4	4	4	5	4	4	5	4	5	4	5	4	5	3	5	6	7	5	7	6	4
1	2	2	1	3	1	1	1	4	4	4	4	4	4	4	4	5	4	4	4	4	4	5	3	4	4	3	4	4	4	5	4	4	4	4	3	3	4	4	4	4	4	4	4	4	5	5	2
1	2	3	1	3	1	1	1	4	3	4	4	4	4	4	4	4	4	4	4	4	4	4	3	4	4	4	4	4	4	5	4	4	4	4	4	4	4	4	4	4	4	5	6	7	5	5	2
2	2	2	1	3	1	1	2	5	5	5	4	5	5	5	5	5	4	5	5	4	5	4	4	4	4	4	4	4	4	4	4	4	4	4	4	4	4	4	4	4	4	6	6	6	6	6	4
1	3	2	1	3	2	3	1	5	5	4	4	2	4	4	4	4	4	4	4	3	3	3	4	4	4	4	4	4	4	4	4	3	3	4	3	4	4	4	4	4	4	6	6	4	6	6	5
2	2	4	1	2	4	2	1	5	5	4	4	5	5	5	5	5	4	3	5	5	4	5	5	5	5	5	4	4	4	4	4	4	4	4	4	4	3	3	3	3	3	3	5	5	5	5	3
2	2	2	1	2	1	3	1	5	5	5	5	5	4	4	4	5	5	4	5	5	4	4	5	3	3	4	4	4	4	4	4	4	4	4	4	3	4	4	4	4	4	7	6	6	7	7	4
1	2	2	1	2	1	4	1	5	5	5	5	5	5	4	2	3	4	4	4	4	4	5	5	5	5	5	4	4	4	4	4	5	5	5	3	3	4	4	4	4	4	7	7	7	7	7	4
1	2	2	1	3	2	3	1	5	5	5	5	5	5	5	5	4	4	4	4	4	4	4	4	4	4	4	4	4	4	4	4	4	4	4	4	4	4	4	4	4	4	5	5	5	5	5	2
1	2	2	1	3	2	3	1	5	5	5	5	5	5	5	5	4	4	4	4	4	4	4	4	4	4	4	4	4	4	4	4	4	4	4	4	4	4	4	4	4	3	4	4	4	4	4	3
1	1	2	1	3	1	1	1	5	5	4	5	4	5	3	5	3	4	4	4	4	4	3	3	5	4	5	4	4	4	4	4	3	5	4	4	4	5	4	5	5	5	5	5	6	5	5	1
1	2	2	1	3	2	3	1	5	5	5	5	5	5	5	5	5	5	5	5	4	4	4	4	4	4	4	4	4	4	4	4	4	4	4	4	4	4	4	4	4	4	5	5	5	5	5	3
1	3	2	1	3	2	1	1	4	5	4	5	5	5	5	4	4	5	4	4	5	4	4	5	4	4	4	4	4	4	4	4	4	4	4	4	4	4	4	4	4	5	6	6	6	6	6	3
2	1	2	1	3	1	1	1	4	5	5	4	4	4	4	4	3	3	3	3	3	3	5	5	5	5	5	4	4	4	4	4	4	5	5	5	5	5	5	5	5	4	7	7	7	7	7	4
1	2	2	1	3	2	3	1	4	5	4	4	4	4	5	4	4	4	3	4	4	3	3	3	4	4	4	4	4	4	4	4	4	4	4	4	3	3	3	3	3	3	7	6	6	6	6	3
2	3	2	1	5	5	2	1	5	4	3	3	4	3	3	4	4	4	5	4	3	3	4	4	4	4	3	4	4	4	4	4	4	4	3	3	4	3	5	4	4	4	6	6	5	5	5	3
1	2	2	1	2	1	2	1	5	4	4	4	4	4	3	5	5	4	4	4	4	4	4	4	4	3	4	4	4	4	4	4	3	5	4	5	3	3	5	4	5	4	6	6	6	7	7	6
2	2	2	1	3	1	3	1	5	4	3	3	4	4	4	4	3	4	4	5	5	5	4	4	4	3	3	4	4	4	4	4	4	4	4	4	4	3	3	3	4	3	5	5	5	5	5	3
1	3	2	1	3	1	2	1	5	4	4	4	5	5	5	5	3	4	5	5	5	5	5	4	4	4	4	4	4	4	4	4	3	3	4	4	4	4	5	4	3	3	5	6	6	6	6	6
1	1	2	1	3	1	2	1	5	4	4	4	5	4	4	3	5	4	4	4	4	4	5	3	5	4	4	4	4	4	4	4	4	4	4	3	4	4	4	4	4	4	7	6	6	6	6	4
2	1	4	1	1	1	1	1	5	4	4	4	5	5	4	4	4	5	5	5	5	5	5	4	5	1	4	4	4	4	4	4	5	5	5	3	3	1	1	1	4	4	4	7	4	5	4	1
1	2	4	1	3	2	3	1	5	4	5	4	5	5	5	3	4	4	4	3	3	4	4	4	4	3	3	4	4	4	4	4	4	3	3	2	2	2	2	2	2	2	3	4	2	3	3	1
1	1	2	1	3	1	1	1	5	4	4	4	4	4	4	4	4	4	4	4	4	4	4	4	4	4	4	4	4	4	4	4	5	5	3	4	4	3	4	4	3	6	6	6	6	6	6
1	2	4	1	3	1	1	1	5	4	4	4	4	4	4	4	4	4	4	5	4	5	5	4	5	3	4	4	4	4	4	4	3	4	5	4	4	4	4	4	4	5	6	6	6	7	6	4
2	4	4	2	3	3	3	2	4	4	4	4	4	4	4	4	4	4	4	4	4	4	4	4	4	4	4	4	4	4	4	4	4	4	4	4	4	4	4	4	4	4	5	5	5	5	5	4
1	3	2	1	3	2	2	2	4	4	4	4	4	4	4	4	4	4	4	4	4	4	4	4	4	4	4	4	4	4	4	4	4	4	3	3	3	4	4	3	3	4	5	4	4	5	5	3
2	1	2	1	3	1	1	2	4	4	4	3	5	4	5	4	4	4	5	5	5	5	4	4	4	4	4	4	4	4	4	4	4	4	4	4	4	3	2	3	2	4	5	6	5	5	5	1
2	2	2	1	3	2	3	1	4	4	4	4	4	4	4	4	4	3	4	4	4	4	4	4	4	4	4	4	4	4	4	4	5	4	4	4	4	2	2	3	3	3	6	6	6	6	6	3
1	2	2	1	3	1	2	1	4	4	4	4	4	4	4	4	4	4	4	5	4	4	4	4	4	4	5	4	4	4	4	4	4	4	4	4	4	4	4	4	4	4	6	6	6	7	6	3
2	1	2	1	3	2	3	1	4	4	3	3	3	4	4	3	4	4	4	4	4	4	5	5	5	5	5	4	4	4	4	4	4	4	5	4	4	4	5	3	5	4	7	7	6	6	6	6
2	3	2	1	3	2	3	1	4	4	3	4	4	4	4	2	2	4	4	4	4	4	4	4	4	3	3	4	4	4	4	4	4	4	3	3	3	4	3	4	4	4	5	5	5	5	5	4
2	2	2	1	3	2	1	1	4	4	4	4	4	4	4	4	4	4	4	4	4	4	4	4	4	4	4	4	4	4	4	4	4	4	4	4	4	4	5	4	5	4	6	6	6	6	6	3
1	2	2	1	3	2	3	1	4	4	4	4	5	5	5	5	5	5	4	4	4	4	4	4	4	4	4	4	4	4	4	4	4	4	4	4	4	4	4	4	4	4	6	6	6	6	6	1
1	2	2	1	2	1	1	1	4	4	4	4	4	4	4	4	4	4	4	4	4	4	4	4	4	4	4	4	4	4	4	4	4	4	4	4	4	4	4	4	4	4	7	7	5	6	6	4
2	2	2	1	3	2	3	1	4	4	4	5	4	4	5	4	4	4	4	4	3	4	4	4	4	5	4	4	4	4	4	4	5	4	5	4	4	3	2	3	3	3	6	6	6	6	6	3
2	2	2	1	3	1	4	1	4	4	4	4	4	4	4	4	4	4	4	4	4	4	4	4	4	4	4	4	4	4	4	4	4	4	4	4	4	4	4	4	4	4	6	6	5	6	6	3
1	2	2	1	3	1	1	1	4	4	4	4	4	4	4	4	4	3	4	4	4	4	4	4	4	4	4	4	4	4	4	4	4	4	4	4	4	4	4	4	4	4	6	6	6	6	5	2
1	1	4	1	1	1	2	1	4	4	3	4	5	4	5	4	5	4	5	4	5	4	4	3	5	3	4	4	4	4	4	4	5	4	4	3	5	4	4	3	4	4	5	5	6	5	4	1
1	1	4	1	2	2	1	1	4	4	4	5	5	4	4	4	4	5	4	4	3	3	4	4	4	4	4	4	4	4	4	4	4	4	4	4	5	4	4	4	4	4	6	6	6	7	7	6
1	3	2	2	3	4	3	1	3	4	4	3	5	4	3	3	2	4	4	4	3	4	4	3	3	3	3	4	4	4	4	4	3	3	4	3	3	3	4	3	3	4	4	4	4	3	3	4
2	4	2	2	3	2	2	1	3	4	3	3	4	4	2	2	3	3	3	4	3	3	2	2	2	3	3	4	4	4	4	4	4	4	3	3	3	3	3	4	4	4	4	2	2	3	3	3
2	4	2	2	3	3	3	1	4	3	4	3	5	4	3	2	2	3	4	3	3	3	4	4	4	4	4	4	4	4	4	4	3	4	3	3	3	4	2	5	5	5	6	5	5	6	6	4
2	1	2	1	3	1	2	1	4	3	3	3	3	3	5	4	4	4	4	4	4	4	4	4	4	4	4	4	4	4	4	4	4	4	4	4	4	4	4	4	4	4	5	5	5	5	5	4
1	2	2	1	3	1	1	1	4	3	4	3	2	4	4	2	2	2	3	3	3	4	4	4	2	2	2	4	4	4	4	4	4	4	4	4	4	3	4	3	4	3	5	4	4	5	5	4
1	2	2	1	3	1	4	1	4	3	3	4	3	3	4	4	4	3	3	4	4	5	5	5	5	5	5	4	4	4	4	4	4	4	4	4	4	4	4	5	4	4	6	5	6	6	5	2
1	3	2	1	2	2	2	1	4	3	5	4	3	4	4	3	3	3	3	3	3	4	4	4	4	3	3	4	4	4	4	4	4	4	4	4	4	3	3	3	3	4	5	5	4	5	5	4
2	2	2	1	3	1	1	1	4	3	4	4	3	3	4	4	3	2	2	3	3	3	3	3	3	4	4	4	4	4	4	4	4	4	4	3	3	2	2	2	2	2	4	4	4	5	4	1
2	2	4	1	3	1	3	1	3	3	4	2	2	5	4	2	2	2	3	2	2	1	3	1	2	1	2	4	4	4	4	4	3	3	3	3	3	2	2	3	2	3	2	5	2	2	2	1
1	6	2	1	3	1	1	1	2	3	3	2	2	4	4	2	2	2	4	4	4	4	4	4	4	4	4	4	4	4	4	4	4	4	4	4	4	4	4	4	4	4	5	5	6	5	6	3
1	1	2	3	5	1	5	1	4	2	3	3	4	3	2	5	3	3	4	3	4	4	4	4	4	3	4	4	4	4	4	4	4	4	4	4	2	3	3	4	3	3	6	5	5	5	6	1
1	5	2	2	3	3	3	1	5	5	4	4	4	3	5	4	4	3	4	3	5	4	4	5	4	4	4	4	4	4	3	4	3	3	3	4	4	3	4	3	4	4	5	6	5	6	5	5
2	2	2	1	4	1	1	1	5	5	4	5	5	5	5	4	5	5	5	5	5	4	5	5	5	4	3	4	4	4	3	4	4	4	5	3	3	4	4	4	4	3	5	5	4	5	5	4
2	2	4	1	3	2	4	1	5	5	4	5	4	3	4	3	4	4	4	4	5	5	4	4	4	3	3	4	4	4	3	4	3	4	4	4	4	4	4	4	4	4	5	6	5	5	6	3
2	1	1	1	2	1	2	1	4	5	5	3	5	5	5	5	5	5	5	4	4	5	5	5	5	5	5	4	4	4	3	4	3	3	4	4	4	2	2	2	1	5	7	7	7	7	7	1
2	2	2	1	3	2	3	1	4	4	4	4	4	4	4	4	4	5	4	5	5	4	4	4	4	4	4	4	4	4	3	4	4	4	4	3	3	2	4	4	5	4	5	6	6	6	5	4
2	2	2	1	2	2	3	1	3	4	3	5	4	4	4	3	4	4	3	5	3	5	3	3	4	4	4	4	4	4	3	4	4	4	3	5	3	4	3	4	4	3	5	5	4	4	5	4
2	7	2	2	3	3	3	2	5	3	4	4	4	4	4	4	4	4	5	5	4	4	5	5	5	5	5	4	4	4	3	4	4	4	4	5	5	4	5	5	5	5	6	5	5	5	5	3
1	8	2	2	1	2	3	1	4	3	3	3	3	4	4	4	3	3	2	4	3	5	4	4	4	5	5	4	4	4	3	4	3	4	4	4	4	5	4	3	4	4	5	6	6	6	6	3
2	6	4	3	3	3	2	1	5	5	5	3	5	3	5	4	4	5	5	5	5	5	5	5	5	5	5	4	4	3	5	4	3	3	3	4	4	3	5	4	5	5	7	7	5	7	7	6
1	3	2	1	3	3	3	1	4	4	4	5	4	4	5	4	4	4	4	4	5	4	5	5	5	3	3	4	4	3	5	4	3	3	3	3	3	4	2	4	3	4	5	6	5	6	6	4
1	5	2	2	2	3	3	1	4	3	5	4	3	3	4	3	4	5	3	3	3	4	3	3	3	2	3	4	4	3	5	4	3	4	5	4	3	2	3	3	4	3	5	6	4	5	6	3
2	1	2	1	1	1	1	2	5	4	4	4	5	5	5	3	4	4	4	4	4	5	4	4	3	4	5	4	4	3	4	4	5	4	3	4	3	4	4	4	3	4	5	6	5	6	5	2
1	6	2	1	1	1	2	1	4	4	4	3	4	4	4	4	3	3	3	3	3	3	4	3	4	3	2	4	4	3	4	4	3	3	4	4	3	3	3	5	4	3	4	4	4	4	4	2
1	2	2	1	2	1	1	1	4	4	4	4	3	4	3	3	4	4	4	3	4	3	4	3	4	4	4	4	4	3	4	4	3	4	3	4	4	4	4	3	4	3	5	6	6	5	5	5
2	2	2	1	3	1	1	1	3	4	5	3	4	4	4	2	3	3	2	4	3	2	4	4	4	3	3	4	4	3	4	4	4	4	3	3	3	2	4	3	3	4	5	7	3	4	5	3
1	2	2	1	3	1	3	1	4	3	4	3	5	3	4	3	3	4	4	4	4	4	4	5	4	4	4	4	4	3	4	4	4	3	4	4	4	4	4	4	4	4	6	6	5	4	4	4
2	2	2	1	3	3	1	1	3	3	3	5	4	4	4	3	4	4	4	3	4	3	5	3	4	3	4	4	4	3	4	4	3	4	4	5	4	4	4	4	4	4	5	5	5	5	5	6
2	4	2	1	3	3	3	1	5	5	5	5	4	4	5	4	4	5	4	4	5	4	4	4	4	3	4	4	4	3	3	4	3	3	3	2	2	3	4	4	4	4	5	5	6	5	5	4

2	1	2	1	3	1	3	1	5	5	5	5	5	3	4	3	4	3	3	3	4	3	5	5	3	3	3	4	4	3	3	4	3	3	3	3	3	4	3	4	3	3	5	4	4	5	5	3
2	5	2	2	4	4	3	1	4	5	5	3	5	5	4	3	3	4	4	4	4	4	4	3	4	4	3	4	4	3	3	4	3	3	3	2	3	3	4	4	4	3	5	5	4	4	4	2
1	1	2	1	3	1	1	1	4	4	4	4	4	4	4	4	4	3	3	5	4	4	3	3	3	3	3	4	4	3	3	4	3	3	3	3	3	3	3	3	3	3	4	5	5	5	5	2
2	2	2	1	3	1	1	1	3	4	4	4	4	4	3	3	3	3	3	3	3	3	3	4	3	3	4	4	3	3	4	4	3	3	3	3	3	3	3	3	4	3	5	4	4	4	5	1
1	3	2	1	2	2	3	1	4	3	3	3	2	3	3	3	3	3	3	3	3	4	4	4	4	3	4	4	4	3	3	4	3	3	4	4	4	4	4	4	4	4	5	4	4	5	6	5
1	2	4	1	2	1	1	1	4	3	4	3	4	3	3	4	4	4	3	3	5	4	4	4	3	4	4	4	4	3	3	4	4	3	4	4	5	4	3	3	2	4	5	6	5	6	5	3
2	2	2	1	1	2	3	1	3	3	3	3	3	3	3	3	3	3	3	3	4	3	4	4	3	4	3	4	4	3	3	4	4	4	3	4	4	3	4	4	4	3	4	5	5	5	5	4
1	1	2	1	1	1	3	2	5	2	4	3	5	3	4	3	4	3	5	4	3	4	4	3	4	5	3	4	4	3	3	4	2	4	4	3	5	4	3	4	2	3	6	5	5	5	6	3
1	7	2	2	1	2	3	1	4	3	5	3	5	4	5	5	5	4	4	4	4	5	5	4	5	5	5	4	4	3	2	4	4	4	4	4	4	4	3	3	3	3	6	5	5	5	5	4
2	2	2	1	2	2	3	1	3	3	3	3	4	4	4	4	3	3	4	4	3	3	4	4	4	4	3	4	4	3	2	4	3	3	3	4	3	3	4	3	4	4	5	4	5	5	5	5
1	2	2	1	3	1	1	1	5	5	5	4	5	4	5	5	5	4	5	4	4	5	5	4	5	5	4	4	5	5	5	4	5	4	5	4	5	5	4	5	4	5	6	6	7	7	7	6
1	2	2	1	3	1	1	2	4	5	5	4	4	4	5	5	5	5	4	5	4	5	4	5	4	5	5	4	5	5	5	4	5	5	4	5	5	5	5	5	5	5	5	6	6	6	7	3
2	2	2	1	3	2	3	1	3	4	4	4	4	4	4	5	4	5	5	5	5	5	5	5	5	4	5	4	5	5	5	4	5	4	5	4	4	4	3	3	3	3	6	6	6	6	6	3
1	2	2	1	3	2	3	1	4	5	5	4	4	5	5	4	5	4	5	5	5	5	5	5	4	4	5	4	5	5	4	4	5	4	5	4	5	4	4	5	4	4	7	7	7	7	7	3
1	2	2	1	3	1	1	2	4	4	4	4	4	4	4	4	4	4	4	5	4	5	4	4	4	5	4	4	5	5	4	4	4	5	4	5	4	4	4	4	5	4	7	6	6	6	6	4
1	2	2	1	3	1	2	1	4	4	5	4	5	4	4	5	4	5	4	5	5	4	3	4	4	5	5	4	5	5	4	4	4	4	5	4	5	4	4	4	4	4	6	6	6	6	6	5
2	2	2	1	3	1	1	1	4	4	5	5	4	5	5	5	5	5	5	4	5	5	5	5	4	5	5	4	5	5	4	4	4	3	4	4	3	4	4	5	5	5	5	4	5	5	6	1
2	1	2	1	2	1	2	1	4	5	5	4	4	5	5	4	5	4	5	5	5	5	5	4	4	4	4	4	5	4	5	4	5	4	4	4	4	5	5	5	4	4	6	6	6	6	6	3
1	1	3	1	3	1	1	1	4	4	4	4	4	4	4	4	4	4	4	4	4	5	4	4	4	5	4	5	4	5	4	4	4	4	4	4	4	4	4	4	4	4	5	5	4	4	5	2
2	5	2	2	3	3	2	1	3	4	5	2	4	4	4	2	2	3	2	2	3	1	3	3	4	3	1	4	5	4	5	4	5	4	3	5	5	5	5	5	5	5	6	2	6	4	6	4
1	2	4	1	2	2	1	1	5	5	5	5	5	5	5	5	5	5	5	5	5	5	5	5	5	5	5	4	5	4	4	4	4	4	3	5	4	5	4	5	4	4	6	6	5	5	6	3
2	1	2	1	1	1	1	1	4	5	5	4	3	5	4	4	3	4	5	5	4	4	5	3	5	5	3	4	5	4	4	4	5	4	5	5	4	4	3	4	5	4	5	5	6	6	6	2
1	2	2	1	2	2	3	2	5	4	3	4	4	3	4	5	4	5	4	4	4	5	4	5	4	4	4	4	5	4	4	4	4	4	4	3	4	4	4	4	4	3	6	6	6	6	5	5
1	2	2	1	3	2	3	1	5	4	4	4	4	5	3	4	4	5	4	4	4	4	5	4	4	5	5	4	5	4	4	4	5	4	4	5	3	4	4	5	4	4	6	6	5	6	5	3
1	1	1	1	1	4	1	1	4	4	4	4	4	4	4	4	4	4	4	4	4	4	4	4	4	4	5	4	5	4	4	4	5	5	5	5	5	5	5	5	5	5	7	7	7	7	7	2
2	2	2	1	2	1	3	1	3	4	3	3	3	2	3	2	4	3	3	4	4	3	4	3	4	3	4	4	5	4	4	4	4	4	3	5	3	4	3	4	4	4	4	5	5	5	5	6
1	1	3	4	5	1	5	1	3	3	3	3	3	3	3	3	3	3	3	3	3	3	3	3	3	3	4	4	5	4	4	4	3	3	4	4	4	5	2	3	3	4	5	5	5	5	4	6
2	2	2	1	3	2	4	1	4	4	5	4	5	5	4	4	3	4	3	4	5	5	4	4	4	5	3	4	5	4	3	4	3	4	4	5	5	4	4	3	4	3	5	5	6	6	6	4
2	1	2	1	1	1	1	1	4	4	3	4	4	3	5	5	4	3	4	5	4	3	4	3	3	4	3	4	5	4	3	4	4	3	4	5	5	5	4	3	4	5	5	6	4	6	5	2
2	2	4	1	3	1	3	1	4	4	3	4	3	5	5	3	3	2	2	2	5	4	3	3	2	4	2	4	5	3	3	4	2	1	4	5	5	4	4	4	5	2	6	2	5	6	3	2
2	2	2	1	3	1	1	1	4	4	5	4	3	4	5	3	4	3	4	4	4	5	4	5	5	5	5	4	5	2	3	4	3	4	4	3	4	4	4	5	5	5	5	6	6	6	6	4
2	8	4	2	4	2	3	1	5	4	5	5	5	5	4	4	1	5	5	5	5	5	3	3	3	1	3	4	2	5	5	5	5	5	5	1	1	3	1	1	2	4	7	7	1	7	7	2
2	3	2	1	3	2	3	1	4	4	5	3	5	5	3	4	2	3	3	3	1	3	4	3	4	1	2	4	2	4	1	5	4	4	4	3	3	5	5	5	5	5	7	5	6	7	7	4
1	2	2	1	3	1	1	2	4	5	4	5	4	3	5	4	4	4	5	4	4	5	5	4	5	3	4	4	3	4	4	5	4	4	3	3	3	4	2	4	4	4	4	5	4	5	4	2
2	4	2	2	1	4	2	1	4	5	3	5	3	5	4	4	4	3	5	4	5	4	4	5	4	3	5	4	3	4	3	5	4	5	4	3	4	3	4	3	4	3	5	6	4	6	4	3
2	1	2	1	3	1	3	2	3	4	4	4	3	4	4	4	3	5	4	4	4	4	5	5	5	5	5	4	3	2	3	5	4	4	5	4	4	4	3	4	4	4	7	7	6	7	7	3
2	3	2	1	4	3	3	1	4	4	4	4	2	3	4	3	3	5	5	5	5	5	5	5	5	5	5	4	3	2	2	5	5	5	5	5	5	4	5	3	5	5	7	4	7	7	7	6
2	2	4	1	2	1	1	1	5	4	5	3	5	5	5	5	5	5	5	5	5	5	3	2	4	1	3	4	4	5	5	5	1	1	1	1	1	3	3	2	2	3	4	5	5	5	5	1
1	2	4	1	3	1	1	1	5	5	4	5	5	5	5	3	5	5	5	5	5	5	4	5	3	5	4	4	4	5	5	5	4	5	5	4	4	5	5	4	5	5	7	6	6	6	1	2
2	1	2	1	3	1	1	1	5	5	4	5	2	4	4	2	4	2	4	4	4	5	5	4	4	4	4	4	4	5	5	5	5	5	5	5	5	5	1	4	2	2	7	7	5	6	6	3
1	1	2	1	2	1	1	1	5	4	4	4	3	5	4	4	3	4	4	4	4	4	4	5	5	4	5	4	4	5	5	5	5	5	4	5	3	5	3	3	4	4	5	5	5	6	6	3
2	2	2	1	3	1	3	1	5	3	4	4	5	5	5	5	5	4	4	4	4	4	5	5	5	4	5	4	4	5	5	5	5	5	5	5	5	5	5	5	5	5	7	7	7	7	7	6
2	2	2	1	1	1	3	1	3	3	4	3	3	3	3	3	3	3	3	3	3	3	3	3	3	5	5	4	4	5	5	5	5	3	5	5	5	5	1	4	3	5	7	7	5	7	7	4
2	2	2	1	3	2	3	1	3	3	4	4	4	3	5	4	4	5	4	5	5	5	5	5	5	5	5	4	4	5	5	5	4	4	5	3	4	3	3	3	4	3	6	6	5	6	6	4
1	1	2	1	2	1	4	1	5	4	5	5	4	4	4	3	3	4	4	5	5	5	5	5	5	5	5	4	4	5	4	5	5	4	4	5	5	5	3	4	5	5	7	5	5	5	7	4
1	2	2	1	3	1	3	2	5	5	4	4	5	5	5	5	5	5	4	4	4	4	5	5	5	4	4	4	4	5	3	5	5	5	5	3	4	3	4	4	4	4	5	6	6	6	6	2
1	2	2	1	1	1	1	1	2	2	4	4	4	4	4	2	4	4	4	4	4	4	2	2	2	2	2	4	4	4	3	5	1	1	1	1	1	4	4	4	5	5	2	5	4	2	2	1
2	1	2	1	3	2	1	2	5	5	4	5	5	3	5	4	5	4	4	5	3	5	5	4	3	4	5	4	4	4	5	5	5	5	5	5	5	4	5	5	3	2	7	5	6	5	7	6
2	2	2	1	2	2	3	1	5	5	4	4	5	4	5	5	4	4	5	4	4	4	3	5	5	5	4	4	4	4	5	5	5	4	4	4	4	4	5	4	4	4	5	5	6	4	4	4
1	8	2	2	2	1	5	1	5	5	4	4	5	5	5	4	4	4	5	5	4	5	5	5	5	5	5	4	4	4	5	5	5	5	5	5	4	4	4	4	4	4	6	7	6	6	6	4
2	8	2	2	3	4	3	1	5	5	5	5	4	4	4	4	4	4	4	5	5	4	4	4	5	5	5	4	4	4	5	5	4	4	4	4	5	5	4	4	4	4	6	6	6	5	5	5
2	1	2	1	3	1	4	1	5	4	5	5	5	5	5	4	5	5	4	5	4	4	5	5	5	5	5	4	4	4	5	5	5	5	5	5	5	4	4	4	5	4	7	6	7	7	7	3
2	1	2	1	2	1	4	1	4	3	4	3	3	3	4	3	3	3	4	4	5	4	4	4	5	5	2	4	4	4	5	5	3	3	4	2	2	4	2	3	2	3	7	6	4	6	4	3
2	2	2	1	3	2	2	1	5	5	5	5	5	5	5	5	5	4	4	4	4	4	5	5	5	5	5	4	4	4	4	5	5	5	5	5	4	4	4	4	4	4	7	7	7	7	7	4
1	1	2	1	3	1	1	1	4	5	4	5	5	4	5	4	4	5	5	4	4	5	5	4	5	5	5	4	4	4	4	5	5	4	5	3	5	5	4	5	4	4	7	6	7	7	7	5
2	1	2	1	3	1	1	1	4	4	3	3	3	4	5	4	3	5	5	5	4	5	5	5	5	5	5	4	4	4	4	5	5	5	5	5	5	5	4	5	5	5	5	5	6	7	6	1
2	2	2	1	2	2	2	1	3	3	4	3	4	5	4	3	3	5	3	5	3	3	5	4	5	5	4	4	4	4	4	5	5	4	5	4	5	5	5	5	5	5	7	5	5	5	6	6
1	1	2	1	2	1	4	2	5	4	4	5	5	5	4	4	3	3	4	4	3	5	3	3	2	2	3	4	4	3	4	5	4	4	4	3	3	3	3	3	3	3	3	3	3	3	4	2
2	2	2	1	3	1	1	1	4	4	3	4	3	4	4	4	4	4	3	4	3	3	5	5	5	5	5	4	4	3	4	5	4	4	4	4	4	3	3	3	3	5	6	6	6	6	6	4
2	2	2	1	3	2	3	1	4	5	4	5	4	3	4	4	4	5	4	3	4	4	5	5	5	5	5	4	4	3	3	5	2	2	5	5	5	4	4	3	4	4	6	7	6	7	7	5
1	1	4	1	3	1	1	1	4	5	4	3	5	4	5	5	5	5	5	5	4	4	5	5	4	5	5	4	4	3	1	5	4	5	4	5	4	4	4	5	5	4	7	6	6	6	5	1
1	1	2	1	1	1	3	1	5	4	5	5	4	5	5	4	3	1	3	4	2	4	2	2	3	3	3	4	5	5	5	5	1	4	3	2	2	4	4	2	2	5	4	4	4	4	3	2
2	5	2	2	2	2	3	1	3	3	3	3	3	4	2	4	3	2	3	4	2	3	1	1	1	1	1	4	5	5	3	5	1	2	2	1	1	2	2	2	1	2	1	1	1	1	1	1
1	2	1	1	3	2	4	1	5	5	5	4	4	5	5	4	5	4	5	5	5	4	5	5	5	5	5	4	5	5	5	5	4	5	4	5	5	4	5	5	4	5	6	6	7	6	7	3
1	2	4	1	3	1	1	1	4	5	5	3	5	5	3	5	4	4	5	5	4	5	5	5	5	5	5	4	5	5	5	5	1	3	5	3	3	5	5	5	5	5	7	4	5	7	7	6
2	3	2	2	4	3	2	2	5	4	4	5	4	4	4	5	5	4	5	5	4	4	5	5	5	4	4	4	5	5	5	5	4	4	3	3	3	4	3	3	4	4	6	6	6	7	6	2
1	1	1	1	3	5	1	1	5	4	3	5	3	5	3	4	4	4	3	5	4	5	3	5	4	5	3	4	5	5	5	5	3	5	5	4	4	5	4	3	4	5	5	6	6	6	6	2
2	1	2	1	2	1	1	1	4	4	4	4	4	4	4	4	4	4	4	4	4	4	4	3	4	3	3	4	5	5	5	5	5	5	5	5	5	4	4	4	4	2	5	4	5	5	5	3
1	2	2	1	3	2	1	1	4	4	4	5	5	4	4	5	4	5	5	5	5	5	4	5	5	5	5	4	5	5	5	5	5	5	5	5	5	5	5	5	5	5	7	7	7	7	7	3
2	2	4	1	3	1	1	1	5	2	5	5	5	5	5	5	5	5	5	4	4	5	4	5	4	4	5	4	5	5	5	5	5	5	5	5	5	5	5	5	4	5	7	1	5	5	5	1
2	1	2	1	3	1	4	1	5	2	5	3	5	5	5	3	4	5	3	3	4	2	5	5	5	5	5	4	5	5	5	5	5	5	5	3	3	2	5	5	5	4	7	2	6	7	7	6
2	1	2	1	1	1	3	1	4	5	4	5	4	4	4	5	4	4	4	5	4	5	4	4	5	5	4	4	5	5	4	5	5	4	4	4	4	4	4	4	4	3	6	6	5	5	5	2
1	4	4	2	3	3	3	1	3	4	5	3	5	5	5	5	5	4	4	4	4	3	3	3	3	1	3	4	5	4	3	5	1	1	1	3	3	2	1	1	1	1	2	7	4	3	3	1
2	2	2	1	3	1	1	1	5	5	4	3	5	5	5	4	4	5	5	4	3	4	4	4	5	4	5	4	5	4	5	5	4	5	5	5	5	5	4	4	3	3	4	7	6	5	6	2
2	2	2	1	3	3	1	1	5	5	3	3	5	5	4	4	5	4	4	4	4	4	4	5	4	3	4	4	5	4	5	5	5	5	5	5	4	1	3	3	3	2	5	7	4	6	6	6
2	2	2	1	3	2	1	1	5	4	4	5	4	5	5	5	5	5	4	4	5	4	4	5	5	4	5	4	5	4	5	5	4	4	5	5	4	5	4	4	5	5	5	6	5	6	6	2
1	4	2	2	5	5	2	1	5	5	5	5	5	5	5	5	5	5	5	5	5	5	5	5	5	5	5	4	5	3	4	5	5	5	5	5	4	4	5	5	5	4	4	6	7	7	6	6
2	2	4	1	3	1	1	2	5	4	3	5	4	5	3	4	4	5	3	5	3	5	4	4	5	3	5	4	5	2	5	5	5	5	4	5	5	5	4	4	5	4	5	6	4	7	5	2
1	2	2	1	2	2	3	1	4	4	3	4	5	4	4	3	4	4	3	4	4	3	5	5	5	5	5	4	5	2	3	5	5	5	5	5	5	5	4	4	5	5	7	6	6	7	7	6
2	2	4	1	1	2	3	1	5	4	5	3	5	5	5	5	5	3	5	3	5	3	5	4	5	5	5	5	5	5	2	2	2	2	3	3	1	1	1	1	1	1	1	5	1	5	4	1
1	3	1	1	3	3	3	1	5	3	5	5	5	5	5	3	3	4	5	4	3	5	3	3	3	3	3	5	5	5	5	2	2	3	3	1	1	5	3	5	3	5	5	3	3	4	4	1
1	3	4	1	3	2	2	1	4	3	5	3	4	4	4	3	2	5	4	4	2	5	1	1	2	1	1	5	5	5	5	2	2	1	1	1	1	4	3	2	2	5	1	4	2	2	2	1
2	1	2	1	2	1	4	2	3	3	3	4	5	5	3	4	5	3	3	4	3	3	3	3	3	3	3	5	5	5	5	2	2	1	1	1	1	1	1	1	2	2	3	4	3	3	3	1
1	2	3	1	1	1	1	1	5	5	4	3	4	5	5	2	2	5	5	3	4	4	3	3	5	2	4	5	5	5	4	2	4	3	3	2	2	2	1	1	3	5	4	6	4	4	3	1
1	2	1	1	2	1	1	1	5	5	5	2	4	5	1	1	1	3	2	2	2	2	2	3	2	1	1	5	5	5	4	2	1	1	1	3	3	1	1	1	2	5	1	4	3	2	2	1
2	2	2	1	3	1	3	1	5	4	4	3	4	5	4	2	4	3	2	4	3	2	3	3	3	4	4	5	5	5	4	2	2	3	2	3	3	2	2	2	3	1	5	5	5	6	6	4
1	1	2	1	2	1	4	1	4	4	4	4	5	4	5	3	5	4	3	5	3	4	1	2	1	1	1	5	5	5	3	2	2	3	2	2	2	2	1	1	5	5	1	1	2	1	1	1
2	2	2	1	3	1	1	1	5	3	4	3	5	5	5	3	5	5	4	4	4	5	4	2	4	1	1	5	5	4	5	2	3	4	5	4	4	2	5	4	4	3	7	7	5	7	7	4
2	2	2	1	1	1	3	2	5	4	3	5	4	4	5	3	4	5	4	5	2	2	1	4	4	2	3	5	5	3	4	2	3	4	3	3	2	1	3	2	3	3	3	5	2	4	6	2
1	2	2	1	3	1	3	1	3	4	3	5	1	1	5	5	5	1	1	5	5	5	2	5	5	1	5	5	5	3	2	2	5	5	2	2	5	5	2	4	5	5	7	7	7	6	7	3
2	1	2	1	1	1	4	1	4	3	2	5	2	4	3	3	2	5	4	3	2	5	2	5	2	3	4	5	4	5	4	3	2	5	2	5	5	3	5	3	3	5	7	3	6	3	7	2
2	1	4	1	1	1	1	2	3	4	5	4	3	4	5	3	5	4	4	3	5	3	4	5	3	5	4	5	4	4	4	3	5	4	3	4	5	4	3	5	4	3	7	5	4	7	6	4
1	2	2	1	1	2	3	1	4	5	4	5	3	5	3	2	5	5	5	5	4	5	3	2	3	4	4	5	4	4	4	3	4	5	3	3	3	2	2	1	4	2	6	5	5	6	7	4
1	4	2	2	4	2	3	2	5	4	4	5	4	3	5	4	5	4	4	3	4	5	3	4	4	4	5	5	4	4	4	3	5	4	3	5	5	4	4	3	4	5	5	7	7	5	7	2
2	2	2	1	3	1	4	1	5	5	5	5	4	4	4	5	3	5	5	3	3	4	3	5	3	5	4	5	4	4	4	3	3	3	4	2	4	3	5	4	3	4	5	6	5	6	6	5
1	2	4	2	3	1	2	2	5	5	4	4	4	3	4	5	4	4	4	5	4	4	4	4	5	4	4	5	4	4	5	3	5	4	4	5	5	4	4	4	4	4	6	6	6	5	6	3
2	2	4	1	3	2	3	1	4	4	3	4	5	5	5	3	4	4	4	3	4	4	3	3	4	2	3	5	4	4	4	3	3	2	2	2	3	4	4	4	4	4	5	6	4	5	5	1
1	5	3	2	1	1	3	2	4	3	4	3	4	3	4	5	5	4	4	3	4	4	4	3	3	5	5	5	4	4	4	3	5	3	5	5	4	5	4	3	3	4	5	4	4	6	6	4
2	5	2	2	3	2	2	2	5	4	2	3	4	3	5	5	4	4	5	3	2	4	3	4	3	5	3	5	4	4	2	3	4	4	5	4	4	4	5	5	4	2	5	6	4	7	6	4
2	2	2	1	3	1	4	1	4	5	5	3	4	5	4	3	3	4	3	2	2	3	2	3	3	2	3	5	4	3	5	3	2	2	3	2	2	3	1	3	4	3	5	6	3	4	4	1
1	2	2	1	2	1	3	1	4	4	4	5	4	4	4	3	3	5	3	3	4	4	4	3	3	3	4	5	4	3	4	3	4	4	3	3	4	3	3	4	3	3	4	4	5	5	5	4
1	4	2	1	3	3	3	1	4	3	4	2	3	3	4	3	4	5	4	3	4	3	4	3	4	3	3	5	4	3	4	3	3	3	4	3	3	3	2	2	3	4	6	5	3	4	6	2
2	2	2	1	3	1	1	1	4	3	4	3	5	2	4	3	5	3	2	2	4	2	4	4	3	3	2	5	4	2	4	3	5	3	4	4	4	2	5	3	5	5	7	7	6	5	4	5
1	1	2	1	2	1	2	1	4	5	4	3	3	4	3	4	4	3	3	5	4	3	5	3	2	5	4	5	5	5	4	3	3	1	5	4	4	3	5	4	4	4	7	4	7	3	6	6
1	2	2	1	3	1	1	1	5	5	5	5	5	5	5	3	4	4	5	5	5	4	4	4	4	2	2	5	5	5	5	3	1	1	1	1	1	2	2	2	2	2	6	6	6	6	6	1
2	1	2	1	3	2	2	1	5	5	5	5	5	5	5	5	5	2	1	1	3	5	5	5	5	3	3	5	5	5	5	3	5	5	5	5	5	5	5	5	5	5	4	7	5	5	5	4
2	2	2	1	3	1	1	1	5	5	4	4	5	5	5	2	3	4	4	4	4	5	3	4	3	2	2	5	5	5	5	3	2	3	2	1	2	3	5	5	4	4	3	6	5	3	3	1
2	1	2	1	1	1	3	1	4	5	4	4	5	4	4	4	5	3	5	4	3	3	2	3	3	3	2	5	5	5	5	3	3	3	2	2	2	4	1	2	1	4	3	5	4	4	5	1
2	2	2	1	2	1	1	1	5	4	5	5	5	5	3	4	4	5	4	4	4	4	5	4	5	5	5	5	5	5	5	3	5	5	5	3	3	4	3	3	3	3	7	7	4	7	7	4
1	3	4	1	3	2	3	1	5	4	3	4	5	5	5	5	5	5	5	5	4	5	4	5	5	5	5	5	5	5	5	3	2	3	3	4	3	3	5	4	4	3	5	4	6	5	5	1
1	1	2	1	3	1	4	1	5	4	5	4	5	5	5	5	3	3	3	4	3	3	4	4	4	2	3	5	5	5	5	3	4	4	4	4	4	2	3	3	3	3	4	6	4	4	4	2
1	3	2	1	2	2	3	1	5	4	5	3	5	5	4	3	3	4	4	3	3	3	4	4	4	4	4	5	5	5	5	3	3	3	3	3	3	4	3	3	3	4	5	6	6	6	5	6
1	2	4	1	2	1	4	1	4	4	4	3	4	4	4	3	4	3	2	3	2	2	3	2	4	1	1	5	5	5	5	3	3	3	3	3	3	4	4	3	4	4	3	4	4	4	4	2
2	1	2	1	3	2	1	1	4	4	4	3	4	5	4	3	3	4	2	3	4	3	2	2	3	2	3	5	5	5	5	3	2	4	4	3	3	4	5	4	3	3	3	6	4	5	5	2
1	2	2	1	3	2	3	1	4	4	4	4	5	5	5	5	5	3	4	3	2	3	4	4	4	4	4	5	5	5	5	3	4	4	3	4	4	1	1	2	2	2	5	5	4	4	4	3
2	2	2	1	1	1	1	1	3	4	3	4	3	3	4	2	2	5	4	3	3	4	1	1	1	1	1	5	5	5	5	3	1	2	1	1	1	2	1	2	2	3	3	6	6	4	5	1
1	2	2	1	2	1	2	1	5	3	4	3	2	3	5	3	4	4	4	4	5	3	3	3	4	3	3	5	5	5	5	3	4	3	4	3	3	3	2	3	2	1	3	4	4	4	4	1
2	3	2	1	1	2	2	1	5	3	5	5	3	3	3	4	4	5	5	5	3	3	2	2	2	1	3	5	5	5	5	3	3	3	3	1	1	1	2	2	1	3	5	4	1	3	4	2
2	2	4	1	3	1	2	2	3	3	3	3	5	5	5	3	3	4	5	5	4	5	3	3	3	3	3	5	5	5	5	3	3	3	3	3	3	3	3	3	3	3	4	4	4	5	4	3

2	7	2	1	1	2	3	1	3	3	3	4	5	4	3	3	4	3	4	3	3	3	3	3	4	4	4	5	5	5	5	3	4	3	3	2	2	2	2	2	2	3	5	7	4	7	7	2
1	8	2	2	2	4	3	1	3	3	5	3	5	5	5	5	5	5	5	4	4	4	5	5	5	5	5	5	5	5	5	3	4	4	4	5	5	5	5	5	5	5	5	5	5	7	7	5
1	5	2	3	3	3	2	1	2	2	2	2	5	3	5	3	2	4	4	5	3	4	2	1	2	2	1	5	5	5	5	3	1	2	1	1	1	1	1	1	1	1	1	3	1	1	1	2
2	6	2	2	2	1	3	1	2	2	2	2	4	4	3	2	2	3	4	4	3	3	4	4	4	3	4	5	5	5	5	3	3	3	3	4	4	4	2	3	2	3	4	4	3	3	3	2
2	2	2	1	2	1	3	1	1	1	2	2	5	5	5	5	5	5	3	5	3	5	5	5	5	2	3	5	5	5	5	3	3	3	3	3	3	5	3	1	5	1	5	7	4	4	4	3
1	2	2	1	3	1	2	1	5	5	5	5	5	5	5	5	5	5	5	5	5	4	5	5	5	5	5	5	5	5	4	3	4	3	4	4	4	4	4	4	3	4	5	5	5	6	6	3
2	9	2	3	1	2	3	1	5	4	5	3	2	3	2	3	3	5	2	3	2	3	5	4	5	4	2	5	5	5	4	3	3	2	3	3	3	4	5	4	4	3	7	5	5	6	6	5
1	2	2	1	3	1	1	1	5	2	4	4	4	5	4	2	4	3	5	4	4	3	4	4	3	2	4	5	5	5	4	3	3	2	4	1	1	2	2	2	3	1	4	5	3	5	5	1
2	2	2	1	3	1	1	2	4	2	5	2	5	5	4	4	5	2	3	4	5	2	4	3	4	2	4	5	5	5	4	3	4	5	4	3	3	2	4	4	2	4	4	7	3	4	4	1
2	2	4	3	3	1	1	1	5	4	5	3	5	5	4	4	4	3	5	4	5	4	5	5	5	4	5	5	5	4	5	3	3	3	3	3	3	1	2	2	2	4	5	7	5	5	5	1
2	2	2	1	3	2	3	1	3	4	4	3	3	3	5	4	3	3	3	3	3	3	3	3	3	4	3	5	5	4	5	3	3	3	3	3	3	2	2	2	3	3	4	5	3	4	4	1
2	1	2	1	1	1	3	1	3	4	5	2	4	4	3	3	3	4	4	5	5	5	5	5	5	5	5	5	5	4	5	3	3	3	3	3	3	3	4	3	3	4	5	6	6	7	6	1
2	2	2	1	3	2	3	1	1	1	1	1	5	5	5	5	4	3	3	3	3	3	3	3	3	3	3	5	5	4	5	3	3	3	3	3	3	2	4	2	2	3	6	7	4	6	6	1
1	1	2	1	2	1	3	1	5	4	4	5	2	3	5	3	4	4	4	5	3	4	4	4	4	4	4	5	5	4	4	3	4	4	2	2	2	2	3	3	3	3	6	6	4	6	6	1
1	2	2	1	2	1	4	1	5	4	5	4	4	4	3	5	4	5	4	3	4	3	3	4	5	5	4	5	5	4	4	3	4	2	3	3	4	4	3	5	5	5	6	6	5	6	7	3
1	2	4	1	3	1	1	1	3	3	4	3	4	4	4	4	4	3	3	3	4	3	4	4	4	3	3	5	5	4	4	3	3	3	3	4	4	4	2	4	4	4	5	6	6	5	5	1
1	2	2	1	3	1	1	1	5	4	2	5	3	2	4	3	4	4	5	5	3	3	3	4	3	4	4	5	5	4	3	3	2	2	2	3	3	3	2	2	3	3	6	5	4	5	5	1
1	4	2	1	1	6	2	1	4	3	5	4	4	4	2	2	2	4	5	4	3	4	4	4	4	4	4	5	5	3	2	3	3	3	3	3	3	2	4	5	4	3	4	4	4	5	5	5
1	3	2	1	3	2	2	2	2	1	1	3	5	4	2	1	3	3	4	4	3	4	3	4	3	1	1	5	5	1	5	3	1	1	1	3	1	3	5	3	5	5	5	7	1	4	5	1
2	2	2	1	3	2	3	1	4	5	4	4	5	5	5	4	3	4	4	4	4	4	4	4	5	5	5	5	4	1	4	4	5	4	4	4	4	4	5	5	5	4	7	7	6	6	6	6
1	2	4	1	1	1	2	2	5	5	5	5	5	5	5	5	5	5	5	5	5	5	5	5	5	5	5	5	4	4	4	4	3	4	4	5	5	5	5	3	3	4	4	4	4	5	7	4
1	2	2	1	1	1	1	2	5	4	4	4	5	5	5	4	5	5	4	5	4	4	5	5	4	5	5	5	4	4	4	4	5	5	5	5	4	4	4	4	5	5	7	6	5	5	7	6
1	2	4	1	1	1	1	1	4	4	5	4	4	5	5	5	5	3	2	4	2	4	4	4	5	4	5	5	4	4	4	4	3	4	4	3	4	4	4	3	4	4	7	7	7	7	6	5
1	1	2	1	3	1	1	1	5	3	5	2	3	4	2	4	4	3	5	5	2	5	3	3	3	1	2	5	4	4	4	4	3	1	2	4	3	1	3	1	5	4	3	5	2	4	6	4
1	1	2	1	3	1	1	1	4	3	3	5	4	3	4	5	3	2	3	3	3	3	3	4	4	4	3	5	4	4	4	4	2	2	1	3	3	2	3	2	3	4	5	6	3	4	4	1
2	3	2	1	1	2	3	1	3	3	3	3	4	4	5	2	2	3	5	5	4	5	4	4	4	2	5	5	4	4	4	4	2	2	5	3	1	4	3	3	3	3	4	1	3	5	4	4
2	1	2	1	2	1	1	1	2	2	2	2	3	3	4	3	3	4	3	2	4	3	3	4	5	5	3	5	4	4	4	4	3	3	3	3	3	2	3	3	3	3	5	4	4	3	4	1
1	6	4	1	3	2	2	1	5	3	4	3	4	3	4	4	4	3	4	5	4	3	3	4	5	4	4	5	5	4	5	4	4	3	4	3	4	5	4	5	4	4	7	5	6	7	5	2
1	2	2	1	2	1	3	1	3	4	5	3	4	5	3	3	4	5	3	3	3	5	3	3	3	4	5	5	5	4	5	4	4	5	3	4	5	4	3	5	5	4	6	6	5	7	7	3
1	1	2	1	3	2	1	2	3	2	3	4	2	3	5	3	3	1	4	3	2	4	1	4	3	3	2	5	5	5	5	4	3	1	5	3	2	5	3	3	3	2	6	3	4	4	5	6
2	3	3	1	3	2	3	2	4	4	5	3	2	5	2	2	1	4	5	5	4	5	3	4	4	3	3	5	5	5	5	4	1	2	1	2	2	2	5	4	5	5	5	7	4	4	5	2
2	2	4	1	2	1	1	1	5	5	4	3	5	5	5	4	5	4	5	4	3	4	5	5	5	4	3	5	5	5	5	4	2	3	2	3	2	3	2	2	3	5	6	7	5	7	7	1
1	1	2	1	3	1	4	1	3	3	2	5	4	3	4	4	3	2	4	5	3	3	3	5	4	3	5	5	5	5	5	4	5	4	5	2	3	3	3	3	5	4	3	6	4	6	4	3
2	2	2	1	3	2	3	1	4	3	5	4	5	4	5	3	3	5	5	4	4	5	2	2	2	1	3	5	5	5	5	4	2	2	1	2	1	3	3	2	3	4	3	4	3	2	2	2
1	2	2	1	3	1	4	1	3	4	5	3	5	5	5	4	5	4	4	5	4	4	4	4	4	2	2	5	5	5	5	4	2	3	3	3	3	2	2	2	2	3	5	4	4	5	5	4
2	2	2	1	3	1	1	1	3	5	5	3	5	5	5	4	3	4	5	5	4	4	5	4	4	2	2	5	5	5	5	4	3	3	4	3	3	3	2	2	3	1	6	7	4	7	7	3
2	1	2	1	2	1	3	1	4	3	5	4	3	5	5	4	3	3	4	5	4	4	5	4	4	5	5	5	5	5	5	4	3	4	5	2	2	2	1	1	1	4	6	7	4	5	5	1
2	2	4	1	3	2	1	1	4	3	4	3	3	5	5	5	5	5	5	5	5	5	4	3	4	3	3	5	4	5	5	4	3	3	3	3	3	1	1	1	1	4	5	7	4	7	7	1
2	3	2	1	2	2	3	1	3	3	4	4	5	5	4	4	4	5	5	4	5	5	4	4	4	5	4	5	4	5	5	4	4	5	5	4	4	5	3	5	4	5	6	5	6	5	6	6
2	7	2	2	2	3	3	1	5	5	5	5	5	4	4	4	4	5	4	4	5	4	5	5	5	4	4	5	4	5	5	4	5	4	5	5	5	5	5	4	4	4	6	6	6	6	6	4
1	2	2	1	2	1	3	1	5	5	5	5	4	5	4	5	5	5	5	5	5	4	5	4	4	4	5	5	4	4	5	4	5	5	5	5	4	5	4	4	4	5	6	6	7	7	7	4
2	2	2	1	2	1	3	1	5	5	5	3	5	5	5	5	5	4	5	5	4	5	5	5	5	3	5	4	4	5	4	4	4	4	4	4	4	4	5	5	4	5	5	5	4	5	5	4
2	3	4	1	3	1	1	1	5	4	3	5	5	4	4	3	4	3	5	5	5	5	5	4	5	5	3	5	4	4	5	4	4	5	5	5	5	5	5	5	5	5	5	6	7	7	7	5
1	8	2	2	3	5	2	1	4	4	5	5	4	5	5	3	3	5	5	5	4	5	5	4	5	5	5	5	4	4	5	4	4	4	4	5	5	3	5	4	4	4	6	6	6	6	6	5
2	1	2	1	3	1	1	1	3	4	3	4	3	4	4	3	4	3	4	4	4	3	5	3	4	4	5	5	4	4	5	4	5	5	5	5	4	5	5	4	5	5	6	5	5	5	6	6
2	2	4	1	3	1	1	1	4	3	3	4	5	3	5	5	3	4	5	5	4	4	4	5	4	2	4	5	4	4	5	4	4	3	5	3	3	2	1	2	1	2	6	7	4	5	5	2
2	3	2	1	1	1	3	2	5	5	5	5	5	5	5	5	5	5	3	4	4	4	5	5	4	4	4	5	4	4	4	4	4	4	4	4	5	5	5	5	5	5	7	7	7	7	7	6
2	1	2	1	2	1	4	1	5	5	5	4	5	4	4	5	4	4	3	3	3	4	4	3	2	2	2	5	4	4	4	4	5	3	4	3	4	4	5	4	3	5	5	5	4	5	4	3
1	2	2	1	3	6	1	1	3	5	5	3	2	4	4	3	3	4	5	4	4	4	4	4	2	4	4	5	4	4	4	4	4	4	4	4	4	4	3	3	3	3	4	4	6	6	6	2
2	1	2	1	2	1	3	1	4	5	4	5	5	4	3	4	5	5	3	4	4	5	4	3	3	5	4	5	4	4	3	4	5	5	4	3	3	3	5	4	4	4	5	7	4	6	5	4
1	2	2	1	2	2	3	1	5	4	4	4	5	4	5	4	4	4	5	5	5	4	3	4	5	4	4	5	4	4	3	4	5	5	5	4	3	4	3	4	3	4	5	5	7	6	7	3
1	1	4	1	2	1	2	1	5	4	4	4	5	4	5	5	3	1	3	1	3	4	4	3	5	4	2	5	4	4	3	4	3	4	4	3	3	2	2	3	1	5	5	5	5	3	6	1
1	3	2	1	1	2	3	1	3	4	3	4	4	4	3	4	3	4	4	3	4	4	4	4	4	3	3	5	4	4	3	4	4	4	4	3	4	4	4	4	3	4	5	5	5	5	5	2
1	7	2	2	1	2	2	1	4	4	3	5	4	3	2	4	3	5	4	3	2	3	5	4	2	4	3	5	4	3	5	4	4	4	3	3	4	3	5	3	4	3	5	7	6	5	6	6
2	2	2	1	2	2	3	1	4	4	4	3	4	4	4	3	4	4	3	4	3	4	4	4	3	3	3	5	4	3	5	4	4	4	4	5	4	5	3	3	4	3	4	5	6	5	6	6
1	2	2	1	3	1	1	1	5	5	3	5	2	4	3	4	3	5	5	3	3	5	4	4	3	5	5	5	4	3	4	4	3	3	4	3	5	4	4	4	5	3	5	6	5	4	4	6
1	3	4	1	1	1	1	1	5	5	5	5	5	5	4	3	3	5	3	5	4	5	4	4	3	3	4	5	4	3	4	4	3	3	4	4	5	4	3	4	4	3	4	5	4	4	4	1
1	3	4	1	3	1	1	1	5	4	5	4	4	4	5	4	5	4	4	4	4	3	5	4	4	4	3	5	4	3	3	4	4	5	5	5	4	5	4	4	4	5	5	6	6	7	6	2
1	2	2	1	3	2	3	1	3	3	3	3	3	3	2	5	3	5	3	5	5	4	4	5	4	3	5	5	4	3	3	4	4	5	3	3	3	5	4	4	3	4	4	6	6	7	5	6
1	3	4	2	3	4	2	1	2	3	5	2	5	3	1	1	5	3	4	5	4	3	1	1	1	1	1	5	5	5	5	4	1	1	1	1	1	1	3	3	3	3	1	1	1	1	1	1
2	2	2	1	3	1	1	2	3	2	4	1	3	4	2	3	4	1	2	2	3	2	3	2	3	1	1	5	5	5	5	4	1	1	1	3	3	3	3	3	5	3	2	3	2	2	2	1
1	4	2	1	2	3	3	1	5	5	5	5	4	5	4	3	5	4	4	4	4	4	4	5	5	5	5	5	5	5	1	4	2	5	1	3	3	5	5	4	5	4	7	7	5	7	7	3
2	2	2	1	3	1	1	2	5	5	3	4	4	5	4	3	4	5	4	3	4	4	2	2	2	2	2	5	5	5	5	4	2	2	2	2	2	2	2	2	2	3	3	5	3	3	4	1
2	1	2	1	3	1	1	1	5	5	4	4	3	5	5	5	3	3	3	4	4	3	5	5	5	2	2	5	5	5	5	4	3	3	4	2	1	1	1	1	1	3	6	4	2	6	5	1
1	7	1	4	3	5	3	2	5	5	5	3	4	4	5	5	4	5	5	5	5	5	3	4	4	3	4	5	5	5	5	4	4	4	4	3	4	4	2	4	5	5	7	5	5	5	6	4
1	8	2	2	3	3	3	1	5	5	5	5	5	5	5	5	5	4	4	4	4	4	5	4	5	4	5	5	5	5	5	4	4	4	4	3	3	4	5	4	4	5	6	6	6	5	5	5
2	4	2	1	3	4	3	1	5	5	5	3	5	5	5	2	2	5	5	5	5	5	4	4	4	4	4	5	5	5	5	4	4	3	4	1	2	4	4	4	5	5	6	7	6	6	6	4
2	7	2	2	3	2	3	1	5	5	5	4	5	4	1	2	2	5	4	4	4	4	4	4	4	3	3	5	5	5	5	4	4	4	4	3	3	3	5	5	5	5	7	3	3	7	7	5
2	2	2	1	3	1	4	1	5	5	4	4	5	5	5	5	5	5	5	5	4	4	4	4	5	4	4	5	5	5	5	4	4	4	4	4	4	5	4	5	4	4	5	5	5	5	6	1
2	3	2	1	3	2	4	2	4	5	5	4	4	5	5	5	5	4	5	5	5	5	5	5	5	5	5	5	5	5	5	4	5	5	5	5	5	5	5	5	4	5	7	7	7	6	6	4
1	2	2	1	2	2	3	1	5	4	5	3	5	5	5	4	4	4	4	5	5	5	5	5	5	4	5	5	5	5	5	4	5	4	5	3	3	3	4	3	4	5	6	7	4	5	5	6
2	6	2	2	3	6	2	1	4	4	4	4	4	4	4	4	4	5	5	5	5	5	4	4	4	4	4	5	5	5	5	4	4	4	4	4	4	4	4	4	4	4	6	5	6	6	6	3
2	2	2	1	3	2	3	1	4	4	4	4	5	5	4	4	4	4	4	4	4	4	5	5	5	3	4	5	5	5	5	4	3	3	3	3	3	2	2	2	2	2	5	5	4	4	4	1
2	8	2	2	1	2	1	1	4	3	5	3	5	5	5	5	5	4	4	4	4	4	5	5	5	3	4	5	5	5	5	4	4	4	4	4	4	5	5	5	5	4	7	7	5	5	5	5
1	2	2	1	3	2	3	1	4	3	4	3	5	4	4	4	5	4	5	4	4	4	4	4	4	3	4	5	5	5	5	4	4	4	4	4	4	2	2	2	2	2	6	6	5	5	5	4
1	2	4	1	3	2	1	2	4	4	4	4	5	5	4	4	5	4	5	4	4	4	4	3	4	3	5	5	5	5	4	4	4	4	4	4	4	4	4	4	5	4	2	4	4	5	5	2
1	2	2	1	1	2	3	1	4	3	4	5	1	5	5	3	3	5	4	2	5	5	3	5	3	4	5	5	5	5	4	4	4	3	1	1	3	2	3	3	4	3	5	4	4	3	7	3
1	8	1	2	3	4	2	1	5	5	5	3	5	5	5	3	5	4	4	4	4	5	5	5	5	5	5	5	4	5	4	4	5	5	5	5	4	4	4	4	4	4	7	7	7	7	7	4
2	2	1	1	2	2	3	1	5	5	4	3	4	4	4	3	5	5	5	4	4	5	5	5	4	4	5	5	5	4	5	4	4	3	4	4	4	5	4	5	4	4	7	7	6	5	7	5
2	2	2	1	3	1	3	1	4	5	4	4	5	4	4	5	4	5	5	5	5	5	5	5	5	5	4	5	5	4	5	4	5	5	4	5	5	4	5	4	5	5	7	6	7	7	6	6
2	7	2	2	1	1	1	1	4	4	3	3	4	4	3	4	4	4	4	4	3	4	5	5	5	5	5	5	5	4	5	4	5	5	3	4	5	3	3	3	4	4	6	5	6	5	5	3
1	2	2	1	2	1	3	1	4	4	5	5	5	5	5	4	5	4	5	5	4	4	5	5	5	4	5	5	5	4	5	4	5	5	5	4	5	4	5	5	4	5	6	5	7	7	7	6
1	1	2	1	3	1	1	1	5	5	5	4	3	4	4	4	4	4	4	4	4	5	4	4	4	5	4	5	5	4	4	4	4	5	4	3	4	4	4	4	4	4	6	5	5	5	5	3
1	2	2	1	3	1	1	1	4	4	4	5	5	5	5	4	4	4	5	4	4	4	5	4	4	4	4	5	5	4	4	4	4	4	4	4	4	4	5	4	5	4	7	6	6	6	6	5
2	2	2	1	2	2	3	1	5	5	4	4	5	3	5	4	3	3	3	5	5	4	3	4	4	5	3	5	5	3	3	4	3	5	4	3	3	4	5	3	5	4	7	5	7	7	4	3
2	6	2	2	2	2	3	1	4	5	3	4	5	4	3	3	4	5	4	5	3	4	5	3	5	3	4	5	4	5	4	5	4	5	4	5	5	4	4	3	3	4	7	6	5	5	6	5
2	8	2	2	1	1	3	1	4	4	5	4	5	4	5	3	4	4	5	4	3	3	4	4	3	3	4	5	4	5	4	5	2	4	3	4	4	3	2	2	2	1	3	2	2	6	2	3
2	2	2	1	3	2	3	1	3	4	5	5	4	4	4	5	4	3	3	4	3	4	5	4	5	3	3	5	4	5	4	5	3	3	2	3	3	2	3	3	3	4	7	6	6	7	7	2
1	2	2	1	3	1	1	1	4	3	5	3	2	4	3	5	4	3	5	4	3	5	5	4	5	5	4	5	4	5	4	5	4	3	3	5	5	4	5	3	2	4	5	6	5	4	7	5
1	2	2	1	2	1	2	2	5	5	5	5	4	3	4	3	5	5	3	5	4	2	3	4	4	5	5	5	4	5	4	5	4	3	5	4	4	4	3	5	4	4	5	4	6	5	5	6
2	2	2	1	3	1	1	1	4	4	4	4	4	4	4	4	4	4	4	4	4	4	4	4	4	4	5	5	4	5	4	5	4	4	4	4	4	2	2	2	2	4	5	5	5	5	5	1
1	2	1	1	3	1	2	1	4	4	4	3	2	3	4	4	3	4	4	5	3	4	4	5	5	4	5	5	4	5	4	5	5	4	4	4	4	4	4	5	5	4	6	6	6	6	7	5
2	2	2	1	3	1	1	2	3	4	4	5	4	4	5	5	2	4	4	2	4	4	4	4	4	3	4	5	4	5	2	5	5	4	4	5	4	4	4	5	4	5	6	5	5	4	5	1
2	2	2	1	3	1	1	1	4	5	4	5	1	4	5	3	4	5	4	4	4	5	5	4	5	5	5	5	4	5	5	5	4	5	3	4	5	5	5	5	5	5	7	7	7	7	7	5
1	1	2	1	2	1	4	1	4	4	4	4	5	5	4	5	4	4	5	5	4	5	4	5	5	5	4	5	4	5	5	5	4	5	4	5	4	5	5	5	4	5	6	7	6	7	6	4
2	1	2	1	1	1	4	2	4	3	4	4	3	4	4	3	3	4	4	4	4	4	4	4	4	5	4	5	4	5	5	5	4	4	4	3	3	2	2	2	2	3	5	6	6	6	6	4
1	2	4	1	2	1	1	1	5	5	5	4	3	4	5	5	3	4	4	4	5	5	5	3	3	5	5	5	4	5	4	5	5	5	3	5	4	5	5	5	4	4	5	7	6	5	6	4
1	3	3	1	3	4	2	1	5	5	4	5	4	5	5	4	5	5	5	4	4	5	4	5	4	5	4	5	4	5	4	5	4	5	5	5	4	4	5	4	4	3	7	5	7	6	7	1
1	2	2	1	3	1	4	1	4	4	5	4	5	5	4	5	4	5	5	4	5	5	4	5	4	5	4	5	4	5	4	5	4	5	5	4	5	4	4	4	5	5	7	7	6	6	6	4
2	5	4	2	2	4	2	1	4	4	4	4	4	4	4	5	4	5	4	4	4	5	4	4	3	4	3	5	4	5	4	5	3	5	4	5	5	3	4	4	4	4	7	5	4	5	4	3
1	2	4	1	3	1	2	1	5	4	3	2	5	1	5	1	5	4	4	3	3	3	3	3	5	4	5	5	4	4	5	5	5	3	3	2	2	2	2	4	4	2	2	3	3	5	4	4
2	3	2	1	2	2	3	2	4	4	4	4	5	4	4	5	4	3	5	5	4	4	5	5	5	4	5	4	4	5	5	5	4	5	5	5	4	5	5	5	5	4	7	7	7	7	7	6
2	7	2	1	4	3	4	1	2	4	3	1	2	3	3	1	3	4	4	4	4	3	5	5	4	3	4	5	4	4	5	5	3	3	1	1	4	3	4	3	3	3	4	3	3	6	5	3
2	4	2	2	1	2	2	1	1	5	5	2	3	3	5	5	5	3	3	3	3	3	3	3	3	5	5	5	4	4	4	5	5	5	3	3	3	3	3	4	5	5	5	5	4	4	4	6
2	1	1	1	3	4	1	1	5	4	4	4	5	5	5	4	5	5	4	5	5	4	5	4	5	5	4	5	4	4	4	5	4	4	5	4	4	5	5	4	5	3	6	5	7	5	6	3
2	1	2	1	1	1	2	1	5	3	3	5	5	5	4	4	3	5	5	5	4	5	5	5	5	5	5	5	4	4	4	5	4	4	4	4	4	4	3	4	4	4	6	6	6	6	6	3
2	2	2	1	2	3	3	1	3	2	5	3	5	3	1	5	1	5	2	4	3	4	4	3	5	4	3	5	4	2	4	5	2	5	4	3	5	5	3	2	3	4	6	3	4	6	5	4
1	2	1	1	3	1	1	1	5	5	5	5	5	5	5	5	5	3	3	3	3	3	1	1	1	1	1	5	5	5	5	5	1	1	1	1	1	1	1	1	1	5	1	5	1	1	1	1
1	1	2	1	1	1	3	1	3	5	5	5	5	5	5	5	5	5	5	5	5	5	5	5	5	5	5	5	5	5	5	5	1	2	1	5	5	4	3	5	5	5	7	7	7	7	7	3
2	1	2	1	1	1	3	2	5	5	5	5	5	5	5	5	5	5	5	5	5	5	5	5	5	5	5	5	5	5	5	5	5	5	5	5	5	5	5	5	5	5	7	7	7	7	7	6
1	1	1	1	3	1	1	2	5	5	5	4	5	5	5	5	5	5	5	5	5	5	5	5	5	5	5	5	5	5	5	5	5	5	5	5	5	5	5	5	5	5	7	7	7	7	7	4
1	1	2	1	3	1	1	2	5	5	5	4	4	4	5	4	5	3	5	3	5	4	5	5	5	5	5	5	5	5	5	5	5	5	5	5	4	3	5	4	5	5	7	7	6	6	5	4
1	10	2	4	1	1	4	2	5	5	5	5	5	5	5	5	5	5	5	5	5	5	5	5	5	5	5	5	5	5	5	5	5	5	5	5	5	5	5	5	5	5	7	7	7	7	7	3
1	2	2	1	3	1	1	1	5	5	5	5	5	5	5	5	5	5	5	5	5	5	5	5	5	5	5	5	5	5	5	5	5	5	5	5	5	5	5	5	5	5	7	7	7	7	7	6
2	7	2	2	2	3	3	1	5	5	5	5	5	5	5	1	1	4	4	4	4	4	5	5	5	5	5	5	5	5	5	5	1	1	1	1	1	3	5	1	5	5	7	7	7	7	7	4
2	2	2	1	3	1	4	1	5	5	4	2	2	4	5	4	4	5	4	5	5	4	4	4	4	4	5	5	5	5	5	5	5	4	5	2	2	2	2	2	4	4	6	6	5	3	6	2
1	2	2	1	5	6	2	1	5	5	5	5	5	5	5	5	5	5	5	5	5	5	5	5	5	5	5	5	5	5	5	5	5	5	5	5	5	5	5	5	5	5	7	7	7	7	7	4
1	2	2	1	2	1	1	1	5	5	3	5	5	5	5	5	5	4	4	5	5	5	5	5	5	5	5	5	5	5	5	5	5	5	5	5	5	5	5	5	5	5	7	7	6	7	7	2
2	2	2	1	1	1	1	1	5	5	5	5	5	5	5	5	5	5	5	5	5	5	5	5	5	5	5	5	5	5	5	5	5	5	5	5	5	5	5	5	5	5	7	7	7	7	7	2

2	6	2	2	2	6	3	1	5	5	5	5	5	5	5	5	5	5	5	5	5	5	5	5	5	5	5	5	5	5	5	5	5	5	5	5	5	5	5	5	5	5	7	7	7	7	7	6
1	3	2	1	3	2	1	1	5	5	5	5	5	5	5	5	5	5	5	5	5	5	5	5	5	5	5	5	5	5	5	5	5	5	5	5	5	5	5	5	5	5	7	7	7	7	7	6
1	2	2	1	1	1	3	1	5	5	5	5	5	5	5	5	5	5	5	5	5	5	5	5	5	5	5	5	5	5	5	5	5	5	5	5	5	5	5	5	5	5	7	7	7	7	7	6
1	2	2	1	2	3	1	1	5	5	5	5	5	5	5	5	5	5	5	5	5	5	5	5	5	5	5	5	5	5	5	5	5	5	5	5	5	5	5	5	5	5	7	7	7	7	7	4
1	2	2	1	1	1	1	1	5	5	5	5	5	5	5	5	5	5	5	5	5	5	5	5	5	5	5	5	5	5	5	5	5	5	5	5	5	5	5	5	5	5	7	7	7	7	7	4
1	8	2	2	4	4	3	1	5	5	5	5	5	5	5	5	5	5	5	5	5	5	5	5	5	5	5	5	5	5	5	5	5	5	5	5	5	5	5	5	5	5	7	7	7	7	7	6
1	2	2	1	2	2	3	1	5	5	5	5	5	5	5	5	5	5	5	5	5	5	5	5	5	5	5	5	5	5	5	5	5	5	5	5	5	5	5	5	5	5	7	7	7	7	7	6
1	2	2	1	1	1	1	1	5	5	5	5	5	5	5	5	5	5	5	5	5	5	5	5	5	5	5	5	5	5	5	5	5	5	5	5	5	5	5	5	5	5	7	7	7	7	7	1
1	1	3	1	1	1	1	1	5	5	5	5	5	5	5	5	5	5	5	5	3	5	5	5	5	5	5	5	5	5	5	5	5	5	5	5	5	5	5	5	5	5	7	7	7	7	7	6
2	1	2	1	2	1	4	1	5	5	5	5	3	3	5	5	5	4	3	4	4	5	4	5	5	5	5	5	5	5	5	5	5	5	4	5	4	5	4	5	5	5	7	7	6	7	5	3
1	1	2	1	2	1	1	1	5	5	5	5	1	5	5	5	5	5	5	5	5	5	5	5	5	5	5	5	5	5	5	5	5	5	5	5	5	5	5	5	5	5	7	7	7	7	7	5
1	1	2	1	2	1	1	1	5	5	5	5	5	5	5	5	5	5	5	5	5	5	5	5	5	5	5	5	5	5	5	5	5	5	5	5	5	5	5	5	5	5	7	7	7	7	7	3
1	2	4	1	3	6	2	1	5	5	5	5	5	5	5	5	5	5	5	5	5	5	5	5	5	5	5	5	5	5	5	5	5	5	5	5	5	5	5	5	4	5	7	1	1	2	2	6
1	3	2	1	3	2	3	1	5	5	5	5	5	5	5	5	5	5	5	5	5	5	5	5	5	5	5	5	5	5	5	5	5	5	5	5	5	3	3	3	3	3	5	6	5	5	6	2
2	2	2	1	3	1	4	1	5	5	5	5	5	5	5	5	5	5	5	5	5	5	5	5	5	5	5	5	5	5	5	5	5	5	5	5	5	5	5	5	5	5	6	6	5	6	6	2
1	4	2	1	3	3	3	1	5	5	5	5	5	5	5	5	5	5	5	5	5	5	5	5	5	5	5	5	5	5	5	5	5	5	5	5	5	5	5	5	5	5	7	7	7	7	7	6
2	8	2	2	1	2	3	1	5	5	5	5	5	5	5	5	5	5	5	5	5	5	5	5	5	5	5	5	5	5	5	5	5	5	5	5	5	5	5	5	5	5	7	7	7	7	7	6
1	1	4	1	3	1	1	1	5	5	4	5	5	5	4	4	5	5	4	4	5	3	5	4	5	5	5	5	5	5	5	5	5	5	5	5	5	5	5	3	5	4	7	7	6	6	6	2
1	2	1	1	3	1	3	1	5	5	5	2	5	5	5	5	5	5	5	5	5	5	5	5	5	5	5	5	5	5	5	5	5	5	5	5	5	5	5	5	5	5	7	7	7	7	7	4
2	1	2	1	3	6	3	1	5	5	5	5	5	5	5	5	5	5	5	5	5	5	5	5	5	5	5	5	5	5	5	5	5	5	5	5	5	5	5	5	5	5	7	7	7	7	7	6
2	2	4	2	3	1	1	1	5	5	5	5	5	5	5	5	5	5	5	5	5	5	5	5	5	5	5	5	5	5	5	5	5	5	5	5	5	5	5	5	5	5	7	7	7	7	7	2
1	2	2	1	3	1	1	1	5	5	5	4	4	4	4	4	4	3	3	4	3	4	3	3	5	5	5	5	5	5	5	5	5	5	5	3	4	3	1	4	3	4	7	7	6	7	5	3
1	2	2	1	3	1	1	1	5	5	5	5	5	5	5	5	5	5	5	5	5	5	5	5	5	5	5	5	5	5	5	5	5	5	5	5	5	5	5	5	5	5	7	7	7	7	7	3
1	1	4	1	2	6	1	1	5	5	5	5	5	5	5	5	5	5	5	5	5	5	5	5	5	5	5	5	5	5	5	5	5	5	5	5	5	5	5	5	5	5	7	7	7	7	7	6
1	2	2	1	2	1	1	1	5	5	5	5	5	5	5	5	5	5	5	5	5	5	5	5	5	5	5	5	5	5	5	5	5	5	5	5	5	5	5	5	5	5	7	7	7	7	7	2
1	2	2	1	4	2	4	1	5	5	5	5	5	5	5	5	5	5	5	5	5	5	5	5	5	5	5	5	5	5	5	5	5	5	5	5	5	5	5	5	5	5	7	7	7	7	7	6
1	1	3	1	2	1	1	1	5	5	5	5	5	5	5	5	5	5	5	5	5	5	5	5	5	5	5	5	5	5	5	5	5	5	5	5	5	5	5	5	5	5	7	7	7	7	7	6
1	2	2	1	3	1	1	1	5	5	5	5	4	5	5	5	5	5	5	5	5	5	5	5	5	5	5	5	5	5	5	5	5	5	5	5	5	2	1	5	5	5	7	7	7	7	7	6
2	3	2	1	3	2	3	1	5	5	4	4	4	4	4	4	4	5	5	5	5	5	4	4	5	4	5	5	5	5	5	5	5	5	5	5	5	5	5	5	5	5	7	7	7	7	7	6
1	3	2	1	1	2	2	1	5	5	4	5	5	5	5	5	5	5	5	5	5	5	5	5	5	5	5	5	5	5	5	5	5	5	5	5	5	5	5	5	5	5	7	7	7	7	7	5
1	1	2	1	3	1	1	1	3	5	3	3	5	5	5	5	5	5	5	5	5	3	1	1	3	3	3	5	5	5	5	5	3	5	5	3	3	3	1	3	5	5	7	4	7	7	7	6
2	1	2	1	2	1	4	1	3	5	4	4	4	4	3	5	4	4	4	4	5	4	5	5	5	5	5	5	5	5	5	5	5	5	5	5	5	5	5	5	5	5	7	6	7	6	7	4
1	1	2	1	1	2	2	2	4	4	5	5	5	5	5	5	5	5	5	5	5	5	5	5	5	5	5	5	5	5	5	5	5	5	5	5	5	5	5	5	5	5	7	7	7	7	7	3
2	5	2	1	1	1	3	2	4	4	5	4	3	4	4	4	4	4	4	5	4	5	5	5	5	5	5	5	5	5	5	5	5	5	5	5	5	5	5	5	5	5	7	7	7	7	7	6
1	2	2	1	1	1	4	2	4	4	5	5	5	5	5	5	5	4	3	5	5	5	5	5	5	5	5	5	5	5	5	5	5	4	5	5	5	5	5	5	5	5	7	7	6	6	5	4
1	10	2	2	2	4	2	1	4	4	5	4	4	4	4	3	3	5	5	5	5	5	5	5	5	5	5	5	5	5	5	5	5	5	5	5	5	5	2	2	2	3	5	6	5	5	5	4
2	4	2	1	2	2	3	1	4	4	4	5	5	5	5	5	5	5	5	5	5	5	5	5	5	5	5	5	5	5	5	5	5	5	5	5	5	5	5	5	4	5	6	6	6	7	7	6
2	2	2	1	3	1	1	1	3	4	4	5	2	4	3	3	3	4	4	4	5	4	3	2	3	3	3	5	5	5	5	5	5	4	4	5	5	3	4	3	3	1	4	7	4	4	4	5
2	2	2	1	3	1	4	1	4	3	3	3	5	5	5	5	5	5	5	5	5	5	5	5	5	5	5	5	5	5	5	5	5	5	5	5	5	3	5	5	5	5	7	7	7	7	7	4
2	2	4	1	3	1	1	1	4	3	5	4	5	3	3	5	5	3	2	3	3	3	4	4	3	4	3	5	5	5	5	5	4	5	5	3	3	4	2	4	2	3	5	6	4	5	5	4
2	2	2	1	3	2	3	1	1	2	2	1	3	5	5	4	4	5	5	5	5	5	5	5	5	5	5	5	5	5	5	5	5	5	5	5	5	5	5	5	5	5	7	7	7	7	7	6
2	1	2	1	3	1	1	2	4	5	4	4	4	4	4	5	4	5	4	5	4	4	4	4	5	5	4	5	5	5	4	5	5	4	5	5	5	5	5	5	5	5	6	6	6	6	5	4
1	2	4	1	2	1	1	1	4	5	4	1	5	5	5	5	4	4	5	2	5	2	2	4	3	1	4	5	5	4	5	5	2	2	2	4	4	5	5	5	5	1	2	5	3	2	2	3
2	2	2	1	3	1	1	1	3	4	5	4	5	3	3	4	4	2	5	4	3	1	2	1	1	1	2	5	5	4	5	5	1	2	3	1	1	2	3	3	5	3	1	3	2	1	2	1
1	1	2	1	1	1	3	1	5	5	5	5	3	1	5	5	3	5	5	5	5	5	5	5	5	5	5	5	5	4	5	5	4	5	4	5	5	4	5	5	4	4	7	7	7	5	6	3
1	10	2	2	4	3	5	1	4	4	3	4	3	3	4	5	4	2	4	5	5	5	5	4	4	3	5	5	5	4	4	5	3	3	5	4	4	5	3	4	3	4	6	5	6	4	6	3
2	2	4	1	3	1	1	1	4	3	4	5	5	4	3	4	5	5	5	5	5	5	3	4	3	4	3	5	5	3	4	5	4	3	4	5	4	4	5	3	2	3	6	7	5	4	4	1
2	7	2	2	3	2	3	1	4	4	4	4	4	5	5	5	5	5	4	4	4	4	5	5	5	5	4	5	5	2	2	5	5	5	5	5	5	5	5	5	5	5	7	7	7	7	7	5
1	7	4	2	1	3	3	1	5	5	5	5	5	5	5	5	5	5	5	5	5	5	5	5	5	5	5	5	5	1	2	5	5	5	5	5	5	5	5	5	5	5	7	7	7	7	7	5
