# Supplementary material for: Predicting the Willingness and Purchase of Travel Insurance During the COVID-19 Pandemic
Source: Front Public Health. 2022 Jul 4;10:907005. doi: 10.3389/fpubh.2022.907005 (PMC9291636; doi:10.3389/fpubh.2022.907005)
Supplement: Supplementary file 2 [file Data_Sheet_2.pdf]

Appendix 1. Survey Instrument

Code	Items
IL1	The main purpose of insurance is to reduce the financial burden of risk faced by the consumer
IL 2	Insurance is the best risk management tool when the chance of loss is low and the loss severity is high
IL 3	Non-disclosure or misrepresentation of information relating to the subject matter insured may cause to reject the insurance claim
IL 4	Consumers are protected against insurance company bankruptcies by state funds that pay some of the claims of bankrupt insurers
HR1	I may become sick from food and/or water and/or polluted air
HR2	There is a possibility of contracting infectious diseases
HR3	Potential health problems are a concern because of poor healthcare infrastructure
HR4	Potential health problems are a concern because of poor transportation infrastructure
HR5	Potential health problems are a concern because of law enforcement infrastructure
HC1	I consider myself as a health-conscious consumer
HC2	I often think about health-related issues
HC3	I am prepared to do anything that is good to health
HC4	I often dwell on my health
HC5	I think that I take health into account a lot in my life
AT1	I think that buying travel insurance is a good choice
AT2	I think buying travel insurance is valuable
AT3	I think buying travel insurance is a good idea
AT4	I think buying travel insurance should be compulsory
AT5	I think buying travel insurance is essential for everyone
PR1	I am unsure whether I can get desired protection from insurance company
PR2	I am unsure whether I can get desired protection from the insurance policy
PR3	I am afraid that insurance company will create unnecessary problems at the time of claim
PR4	I am unsure whether the hospitals in overseas accept the insurance policy
SN1	My social groups think I should purchase travel insurance
SN2	People who influence my decision think that I should purchase travel insurance
SN3	People whose opinions I value think that I should purchase travel insurance
SN4	People who are close to me think that I should purchase travel insurance
SN5	People will like it if I purchase travel insurance
PB1	I have sufficient knowledge to purchase travel insurance
PB2	I can buy travel insurance without any help from anyone
PB3	I have resourceful source about travel insurance purchasing
PB4	I can buy travel insurance reasonably well on my own
PB5	I can handle any (money, time, information related) difficulties associated with my buying decision
WTI1	I expect to purchase travel insurance in the future
WTI2	I would like to know how a travel insurance plan works overseas
WTI3	I know the value of travel insurance and want to purchase as soon as possible
WTI4	I am likely to purchase travel insurance in the future
WTI5	Given the chance, I predict I will purchase travel insurance in future
PTI	Did you ever purchase travel insurance?

Note: IL: Insurance Literacy; HR: Perceived Health Risk; HC: Health Consciousness; AT: Attitude towards Travel Insurance; PR: Perceived Product Risk; SN: Subjective Norms; PN: Perceived Behavioural Control; WTI: Willingness to Purchase Travel Insurance; INC: Average Monthly Income; PTI: Purchase Travel Insurance

Appendix 2. Discriminant Validity

	IL	HR	HC	AT	PR	SN	PB	WTI	INC	PTI
Fornell-Larcker Criterion										
IL	0.765									
HR	0.480	0.711								
HC	0.372	0.417	0.770							
AT	0.401	0.331	0.397	0.852						
PR	0.181	0.261	0.234	0.136	0.832					
SN	0.330	0.315	0.346	0.684	0.177	0.855				
PB	0.272	0.285	0.348	0.441	0.162	0.549	0.824			
WTI	0.383	0.308	0.420	0.731	0.157	0.682	0.523	0.851		
INC	0.084	0.067	0.096	0.043	-0.006	0.012	0.144	0.046	1.000	
PTI	0.157	0.118	0.178	0.414	0.021	0.420	0.415	0.453	0.206	1.000
Heterotrait-Monotrait Ratio (HTMT)										
IL	-									
HR	0.638	-								
HC	0.471	0.525	-							
AT	0.483	0.395	0.455	-						
PR	0.231	0.324	0.276	0.153	-					
SN	0.394	0.368	0.393	0.755	0.196	-				
PB	0.334	0.342	0.403	0.491	0.181	0.609	-			
WTI	0.464	0.371	0.487	0.802	0.180	0.743	0.571	-		
INC	0.098	0.085	0.107	0.045	0.011	0.017	0.156	0.047	-	
PTI	0.182	0.131	0.193	0.436	0.022	0.440	0.439	0.465	0.206	-

Note: IL: Insurance Literacy; HR: Perceived Health Risk; HC: Health Consciousness; AT: Attitude towards Travel Insurance; PR: Perceived Product Risk; SN: Subjective Norms; PN: Perceived Behavioural Control; WTI: Willingness to Purchase Travel Insurance; INC: Average Monthly Income; PTI: Purchase Travel Insurance

Appendix 3. Loadings and Cross-Loading

Code	IL	HR	HC	AT	PR	SN	PB	WTI	INC	PTI
IL 1	0.813	0.383	0.297	0.309	0.135	0.253	0.180	0.300	0.062	0.085
IL 2	0.821	0.363	0.267	0.330	0.128	0.277	0.215	0.317	0.048	0.125
IL 3	0.713	0.343	0.269	0.286	0.160	0.171	0.186	0.245	0.120	0.145
IL 4	0.706	0.378	0.306	0.301	0.134	0.302	0.250	0.307	0.030	0.129
HR1	0.328	0.646	0.278	0.186	0.143	0.149	0.144	0.145	0.084	0.057
HR2	0.376	0.643	0.267	0.208	0.146	0.149	0.144	0.170	0.113	0.037
HR3	0.356	0.747	0.286	0.248	0.174	0.175	0.140	0.207	0.038	0.076
HR4	0.334	0.753	0.320	0.266	0.189	0.305	0.287	0.277	0.014	0.108
HR5	0.324	0.757	0.328	0.255	0.262	0.309	0.271	0.270	0.014	0.127
HC1	0.316	0.316	0.719	0.274	0.169	0.246	0.272	0.301	0.076	0.139
HC2	0.294	0.326	0.785	0.271	0.184	0.203	0.206	0.274	0.100	0.081
HC3	0.253	0.287	0.783	0.305	0.112	0.242	0.250	0.323	0.046	0.135
HC4	0.289	0.358	0.756	0.339	0.239	0.320	0.297	0.343	0.065	0.133
HC5	0.283	0.314	0.802	0.327	0.187	0.303	0.301	0.362	0.087	0.187
AT1	0.355	0.287	0.331	0.860	0.094	0.551	0.334	0.625	0.051	0.359
AT2	0.361	0.291	0.356	0.884	0.097	0.560	0.357	0.623	0.069	0.350
AT3	0.353	0.286	0.350	0.871	0.124	0.572	0.359	0.647	0.030	0.337
AT4	0.303	0.254	0.300	0.812	0.117	0.626	0.426	0.613	0.001	0.363
AT5	0.336	0.290	0.353	0.832	0.150	0.612	0.405	0.608	0.031	0.357
PR1	0.139	0.197	0.194	0.127	0.883	0.176	0.167	0.163	-0.007	0.041
PR2	0.152	0.250	0.213	0.129	0.898	0.168	0.177	0.135	-0.007	0.006
PR3	0.163	0.204	0.167	0.108	0.775	0.132	0.112	0.099	0.009	0.016
PR4	0.162	0.228	0.205	0.084	0.765	0.099	0.063	0.113	-0.013	-0.003
SN1	0.280	0.247	0.291	0.571	0.162	0.844	0.446	0.552	-0.010	0.353
SN2	0.321	0.297	0.317	0.603	0.156	0.864	0.473	0.606	0.028	0.377
SN3	0.304	0.273	0.303	0.611	0.139	0.862	0.442	0.626	0.007	0.384
SN4	0.256	0.276	0.289	0.570	0.146	0.850	0.487	0.554	0.020	0.336
SN5	0.243	0.251	0.277	0.566	0.152	0.856	0.501	0.571	0.006	0.342
PB1	0.221	0.222	0.324	0.419	0.148	0.524	0.819	0.476	0.092	0.348
PB2	0.220	0.252	0.262	0.345	0.128	0.449	0.857	0.393	0.154	0.349
PB3	0.227	0.238	0.279	0.398	0.116	0.505	0.867	0.458	0.115	0.385
PB4	0.210	0.228	0.264	0.340	0.115	0.427	0.849	0.425	0.104	0.376
PB5	0.244	0.238	0.299	0.296	0.161	0.333	0.719	0.387	0.136	0.238
WTI1	0.319	0.252	0.365	0.669	0.134	0.625	0.495	0.882	0.058	0.429
WTI2	0.304	0.286	0.357	0.465	0.170	0.414	0.250	0.705	-0.004	0.200
WTI3	0.342	0.279	0.353	0.616	0.116	0.614	0.541	0.835	0.071	0.414
WTI4	0.331	0.265	0.372	0.673	0.122	0.603	0.443	0.909	0.039	0.420
WTI5	0.340	0.249	0.354	0.657	0.148	0.608	0.441	0.905	0.019	0.412
INC	0.084	0.067	0.096	0.043	-0.006	0.012	0.144	0.046	1.000	0.206
PTI	0.157	0.118	0.178	0.414	0.021	0.420	0.415	0.453	0.206	1.000

Note: (1) IL: Insurance Literacy; HR: Perceived Health Risk; HC: Health Consciousness; AT: Attitude towards Travel Insurance; PR: Perceived Product Risk; SN: Subjective Norms; PN: Perceived Behavioural Control; WTI: Willingness to Purchase Travel Insurance; INC: Average Monthly Income; PTI: Purchase Travel Insurance; (2) The Italic values in the matrix above are the item loadings and others are cross-loadings
